# 2025 abstracts from the 80th Annual Scientific Meeting of the Canadian Society of Allergy and Clinical Immunology

**DOI:** 10.1186/s13223-026-01031-3

**Published:** 2026-07-09

## Allergic Rhinitis/Asthma

## 1

### Clinical efficacy of biologics in severe pediatric asthma: a systematic review and meta-analysis

#### Chung, Jason^1^, Yu, Andrew^2^, Chung, Zinnia^3^, Nazarali, Samina^1^, Waserman, Susan^4^, Jeimy, Samira^1^

##### ^1^Division of Clinical Immunology and Allergy, Department of Medicine, Western University, London, ON, ^2^University of Alberta Faculty of Medicine & Dentistry, Edmonton, AB, ^3^Michael G. DeGroote School of Medicine, McMaster University, Hamilton, ON, ^4^Division of Clinical Immunology and Allergy, Department of Medicine, McMaster University, Hamilton, ON

*Allergy, Asthma & Clinical Immunology* 2026, **22(2)**: 1

**Background** Asthma is the most common chronic respiratory disease in children, affecting approximately 14% of all children worldwide. The successful treatment of asthma focuses on reducing acute asthma attacks, minimizing symptoms and minimizing the need for rescue treatments. For patients with severe asthma, various biologic treatments have emerged in the last two decades. The objective of this systematic review is to compare the effectiveness of various biologics in severe childhood asthma, as measured by reduction annual exacerbation rates and changes in percent predicted forced expiratory volume in 1-s (FEV1).

**Methods** A systematic literature search was conducted on five databases from 1974 to 2025 for English publications. Randomized and non-randomized studies with pediatric (mean age < 18 years) asthma patients receiving a biologic (benralizumab, dupilumab, omalizumab or mepolizumab) was included.

**Results** Thirty-two studies (n = 6,372) were eligible for meta-analysis. The mean increase in percent predicted FEV1 pre- and post- biologic treatment was 12.29%(95%CI:12.13,12.45) in the dupilumab group and 6.04%(95%CI:3.45,8.63) in the omalizumab group. There was a 3.45%(95%CI:2.02,4.87) increase of percent predicted FEV1 in the placebo group. The mean change in the annual asthma exacerbation rates pre-and post-biologic treatment was -2.94 exacerbations per year (95%CI:-3.89,-2.00) in the omalizumab group, -2.24 exacerbations per year (95%CI:-2.42,-2.07)in the dupilumab group, -1.74 exacerbations per year (95%CI:-2.04,-1.44) in the mepolizumab group and -1.45 exacerbations per year (95%CI-1.61,-1.28) in the placebo group.

**Conclusions** Childhood asthma is a global health issue with significant implications on childhood development and healthcare costs. In the past two decades, multiple biologic therapies have been approved for the treatment of severe childhood asthma. We demonstrate the clinical efficacy of these biologics measured by a meta-analysis. Future research is required to identify biomarkers and tools to personalize management plans for the specific asthma phenotype of the patient.

## 2

### Dupilumab improved chronic rhinosinusitis with nasal polyps (CRSwNP) and asthma outcomes more than omalizumab: EVEREST head-to-head phase 4 trial

#### De Corso, Eugenio^1^, Canonica, G. Walter^2^, Heffler, Enrico^2^, Springer, Michał^3^, Grzegorzek, Tomasz^4^, Viana, Miguel^5^, Horváth, Zsuzsanna^6^, Mullol, Joaquim^7^, Gevaert, Philippe^8^, Michel, Justin^9^, Wagenmann, Martin^10^, AlKhouri, Rami^11^, Zaghloul, Sherif^12^, Zhang, Mei^12^, Corbett, Mark^12^, Nash, Scott^13^, Angello, James T.^12^, Radwan, Amr^14^, Deniz, Yamo^13^, Martin, Antonio^15^, Hellings, Peter W^16^

##### ^1^Otorhinolaryngology – Head and Neck Surgery, A. Gemelli Hospital Foundation IRCCS, Rome, Italy, ^2^Personalized Medicine, Asthma and Allergy, Humanitas Research Hospital, Milan, Italy, ^3^Specialized Non-Public Health Care Facility Alergologia Plus, Allergy Diagnostics and Therapy Center, Poznań, Poland, ^4^Department of Otorhinolaryngology, Angelius Provita Medical Center, Katowice, Poland, ^5^Otorhinolaryngology Department, Hospital Pedro Hispano, Matosinhos, Portugal, ^6^Otorhinolaryngology Department, Szent Imre University Teaching Hospital, Budapest, Hungary, ^7^Rhinology Unit & Smell Clinic, ENT Department, Hospital Clínic Barcelona, FRCB-IDIBAPS, Universitat de Barcelona, CIBERES, Barcelona, Catalonia, Spain, ^8^Ear, Nose and Throat Department, Ghent University Hospital, Ghent, Belgium, ^9^Aix Marseille University, APHM, CNRS, IUSTI, La Conception University Hospital, ENT-HNS Department, Marseille, France, ^10^Department of Otorhinolaryngology, Düsseldorf University Hospital (UKD), Düsseldorf, North Rhine-Westphalia, Germany, ^11^Sanofi, Mississauga, ON, ^12^Sanofi, Morristown, NJ, USA, ^13^Regeneron Pharmaceuticals Inc., Tarrytown, NY, USA, ^14^Regeneron Pharmaceuticals Inc., Uxbridge, United Kingdom, ^15^Sanofi, Cambridge, MA, USA, ^16^University Hospitals Leuven, Leuven, Belgium

*Allergy, Asthma & Clinical Immunology* 2026, **22(2)**: 2

**Background** Dupilumab and omalizumab are efficacious treatments for severe, uncontrolled CRSwNP with coexisting asthma, but head-to-head efficacy and safety studies are lacking. Dupilumab and omalizumab are efficacious treatments for severe, uncontrolled CRSwNP with coexisting asthma, but head-to-head efficacy and safety studies are lacking.

**Methods** EVEREST (NCT04998604) is a multicenter, double-blind phase 4 trial comparing the efficacy and safety of dupilumab and omalizumab. Adults with severe CRSwNP and coexisting asthma were randomized to dupilumab 300 mg every 2 weeks (W) or omalizumab 75–600 mg every 2W or 4W for 24W on background mometasone furoate nasal spray. Primary endpoints were W24 change in nasal polyp score (NPS, 0–8) and University of Pennsylvania Smell Identification Test (UPSIT, 0–40).

**Results** 360 patients were randomized (dupilumab, n = 181; omalizumab, n = 179). Baseline characteristics were comparable between groups. Improvements were significantly greater with dupilumab than omalizumab for all primary and secondary efficacy endpoints. Least squares mean differences [LSMDs] in change from baseline at W24, dupilumab over omalizumab, were –1.60 [95% CI –1.96, –1.25], p < 0.001 for NPS, and + 8.0 [+ 6.3, + 9.7], p < 0.001 for UPSIT. Sino-Nasal Outcome Test (SNOT-22) improvements were substantially better with dupilumab than omalizumab in functional domain score (range 0–5): W24 LSMD –0.5 [95% CI –0.7, –0.3]. Improvements in asthma outcomes were greater with dupilumab than omalizumab: W24 LSMDs, dupilumab over omalizumab, were + 0.15 L [95% CI + 0.05, + 0.26] for pre-bronchodilator forced expiratory volume in 1 s, and–0.48 –0.65, –0.31] for 7-item Asthma Control Questionnaire score (range 0–6). Safety profiles were comparable.

**Conclusions** Dupilumab demonstrated superiority over omalizumab for all outcomes pre-specified in the testing hierarchy in patients with severe CRSwNP and coexisting asthma. This study supports the efficacy of dupilumab in comorbid type 2 respiratory diseases vs an active biologic comparator and supports the known safety profiles of dupilumab and omalizumab.

## 3

### Assessing the safety of nasal allergen challenge in individuals with allergic rhinitis and moderate to severe asthma

#### Davis, Abigail^1, 2^, Garvey, Sarah^2^, Botting, Hannah^2^, Steacy, Lisa M.^2^, Ellis, Anne K.^1, 2^

##### ^1^Department of Medicine, Queen’s University, Kingston, ON, ^2^Allergy Research Unit, Kingston Health Sciences Centre (KHSC) – KGH Site, Kingston, ON

*Allergy, Asthma & Clinical Immunology* 2026, **22(2)**: 3

**Background** Nasal Allergen Challenge (NAC) is a controlled method to induce allergic rhinitis symptoms for mechanistic and interventional studies. While NAC safety has been evaluated in allergic rhinitis with or without asthma [1], its safety in participants with moderate-to-severe asthma has not been assessed using our protocol.

**Methods** Seventeen participants (n = 11 allergic, n = 6 non-allergic) underwent NAC with house dust mite, ragweed, Timothy grass, or birch allergen extract. Assessments of Total Nasal Symptom Score (TNSS; range 0–12; sum of rhinorrhea, nasal congestion, sneezing, and itchy nose), Total Asthma Safety Score (TASS; range 0–12; sum of coughing, wheezing, shortness of breath, and chest tightness), peak expiratory flow rate (PEFR), and forced expiratory volume in 1 s (FEV₁) were measured pre- and up to 24 h post-NAC. TNSS and TASS were analyzed using mixed effects models, while PEFR and FEV₁ were analyzed using two-way ANOVA. Multiple comparisons were corrected using Šídák’s test.

**Results** TNSS was higher in allergic compared to non-allergic participants (mean difference = 2.74, 95% CI: 0.969–4.43, p = 0.005), with significant differences at 15 min, 30 min, and 1-h post-NAC (p < 0.001). TASS was significantly higher in allergic participants (mean difference = 0.684, 95% CI: 0.027–1.34, p = 0.042), however, most participants experienced only mild (n = 7) or no (n = 2) asthma symptoms. One participant reported severe asthma symptoms (TASS = 9 at 15 min) and was treated with salbutamol, without the need for further intervention. No changes were observed in FEV₁ or PEFR between pre- and 24 h post-NAC in allergic participants.

**Conclusions** NAC elicited the anticipated upper and lower airway responses in individuals with allergic rhinitis and moderate-to-severe asthma. These results support the preliminary safety of our NAC protocol in this population when conducted under appropriate medical supervision, which is already standard practice for NAC.

**References**

1. Eguiluz-Gracia I, Testera-Montes A, González M, Pérez-Sánchez N, Ariza A, Salas M, et al. Safety and reproducibility of nasal allergen challenge. Allergy. 2019 Jun;74(6):1125–34.

## 4

### Dupilumab reduces mucus burden on patients with uncontrolled asthma regardless of baseline FeNO or asthma duration: results from the VESTIGE study

#### Pérez de Llano, Luis A.^1^, Dunican, Eleanor M.^2, 3^, Castro, Mario^4^, Papi, Alberto^5^, Bourdin, Arnaud^6^, Comellas, Alejandro P.^7^, Caminati, Marco^8^, Altincatal, Arman^9^, Gall, Rebecca^10^, Awad, Hashem^9^, Bhatt, Surya P.^11^

##### ^1^Hospital Universitario Lucus Augusti, Lugo, Spain, ^2^Department of Medicine, School of Medicine, University College Dublin, Dublin, Ireland, ^3^Education and Research Centre, St. Vincent’s University Hospital, Dublin, Ireland, ^4^Department of Medicine, University of Kansas School of Medicine, Kansas City, KS, USA, ^5^Department of Cardiorespiratory Medicine, Respiratory Medicine Unit, University of Ferrara, S. Anna University Hospital, Ferrara, Italy, ^6^Department of Respiratory Diseases, PhyMedExp, University of Montpellier, INSERM CNRS, Montpellier, France, ^7^University of Iowa, Iowa City, IA, USA, ^8^Verona Integrated University Hospital and University of Verona, Verona, Italy, ^9^Sanofi, Cambridge, MA, USA, ^10^Regeneron Pharmaceuticals Inc., Tarrytown, NY, USA, ^11^Division of Pulmonary, Allergy and Critical Care Medicine, University of Alabama at Birmingham, Birmingham, AL, USA

*Allergy, Asthma & Clinical Immunology* 2026, **22(2)**: 4

**Background** Mucus plugging contributes to airflow obstruction in asthma and is driven by type 2 inflammation mediated by interleukin (IL)-4 and IL-13. Dupilumab, a monoclonal antibody targeting the IL-4/IL-13 receptor, reduces mucus burden. This post hoc analysis of VESTIGE (NCT04400318) evaluated changes in mucus score and volume in patients stratified by (1) baseline fractional exhaled nitric oxide (FeNO) and (2) asthma duration.

**Methods** Adults (21–70 years) with uncontrolled, moderate-to-severe asthma and elevated type 2 biomarkers (blood eosinophils ≥ 300 cells/µL and FeNO ≥ 25 ppb) were randomized to receive dupilumab 300 mg (n = 72) or placebo (n = 37) every 2 weeks for 24 weeks. Mucus plug score (range 0–18) and mucus volume were assessed via multidetector computed tomography. Outcomes were analyzed by baseline FeNO (< 50 [n = 52] vs ≥ 50 ppb [n = 42]) and asthma duration (< 10 [n = 30], ≥ 10– < 20 [n = 29], and ≥ 20 years [n = 35]).

**Results** At Week 24, in patients with baseline FeNO, dupilumab vs placebo significantly reduced mucus score (< 50 ppb: least squares mean difference [95% CI]: -5.1 [-7.10, -3.09]; *P* < 0.001; ≥ 50 ppb: -3.2 [-6.21, -0.24]; *P* < 0.05) and mucus volume (< 50 ppb: -0.12 mL [-0.18, -0.05]; *P* < 0.001; ≥ 50 ppb: -0.07 mL [-0.12, -0.02]; *P* < 0.01). In patients with asthma duration, dupilumab vs placebo significantly reduced mucus score (< 10 years: -6.0 [-9.87, -2.10]; *P* < 0.05; ≥ 10– < 20 years: -3.4 [-6.23, -0.59]; *P* < 0.05; ≥ 20 years: -4.3 [-6.80, -1.78]; *P* < 0.05) and mucus volume (< 10 years: -0.1 [-0.20, -0.04]; *P* < 0.05; ≥ 10– < 20 years: -0.09 [-0.17, -0.001]; *P* = 0.05; ≥ 20 years: -0.1 [-0.17, -0.04]; *P* < 0.05).

**Conclusions** Dupilumab reduced mucus burden across baseline FeNO and asthma duration subgroups, supporting its benefits in patients with uncontrolled type 2 asthma.

## 5

### Impact of dupilumab on fractional exhaled nitric oxide in asthma by baseline inhaled corticosteroid dose and clinical remission status

#### Pavord, Ian D.^1^, Bruselle, Guy G.^2^, Busse, William W.^3^, Altincatal, Arman^4^, Ledanois, Olivier^5^, Gall, Rebecca^6^, Canonica, Walter G.^7, 8^

##### ^1^Respiratory Medicine Unit, Nuffield Department of Medicine, University of Oxford, Oxford, United Kingdom, ^2^Ghent University Hospital, Ghent, Belgium, ^3^University of Wisconsin School of Medicine and Public Health, Madison, WI, USA, ^4^Sanofi, Cambridge, MA, USA, ^5^Sanofi, Paris, France, ^6^Regeneron Pharmaceuticals Inc., Tarrytown, NY, USA, ^7^Humanitas University, Milan, Italy, ^8^IRCCS Humanitas Research Hospital, Milan, Italy

*Allergy, Asthma & Clinical Immunology* 2026, **22(2)**: 5

**Background** Dupilumab, a fully human monoclonal antibody that blocks the interleukin-4/13 receptor, significantly reduced exacerbations and improved lung function in patients with moderate-to-severe asthma in the phase 3 QUEST study (NCT02414854), with greatest benefits in patients with elevated type 2 biomarkers (blood eosinophils ≥ 150 cells/µL or fractional exhaled nitric oxide [FeNO] ≥ 20 ppb). This analysis evaluated the impact of dupilumab on FeNO in patients from QUEST with elevated type 2 biomarkers at baseline, stratified by baseline inhaled corticosteroid (ICS) dose (medium or high) and clinical remission status at Week 52.

**Methods** Patients received add-on dupilumab 200/300 mg or volume-matched placebo every 2 weeks for 52 weeks. Clinical remission at Week 52 was defined as: no exacerbations or oral corticosteroid use during the study period, stable (pre- or post-bronchodilator forced expiratory volume in 1 s decrease < 5% from baseline) or improved lung function, and 5-item Asthma Control Questionnaire score < 1.5. Endpoints assessed were percentage change from baseline in FeNO for dupilumab vs placebo recipients.

**Results** In patients on medium-dose ICS at baseline, significantly greater reductions in FeNO (ppb) were seen by Week 52 for dupilumab vs placebo in both patients with clinical remission (median [Q1, Q3]: -48.14 [-67.44, -25.00] vs -15.48 [-42.28, 25.81]; n = 188 vs 58; *P* < 0.001) and without clinical remission (-32.14 [-55.81, -9.09] vs -0.82 [-32.96, 22.03]; n = 161 vs 120; *P* < 0.001). Similarly, among patients on high-dose ICS, significantly greater reductions in FeNO (ppb) were seen for dupilumab vs placebo for patients both in those with clinical remission (-48.05 [-64.05, -27.68] vs -13.72 [-40.51, 16.95]; n = 156 vs 52; *P* < 0.001) and without clinical remission (-38.89 [-59.18, -15.39] vs -10.13 [-32.38, 28.95]; n = 207 vs 138; *P* < 0.001).

**Conclusions** Treatment with dupilumab vs placebo significantly reduced FeNO regardless of baseline ICS dose or remission status in patients with moderate-to-severe asthma and elevated type 2 biomarkers.

## 6

### Pediatric asthma pre- and post-Covid trends in Alberta, Canada: a retrospective population-based study

#### Heibati, Behzad, Gill, Amanpreet, Moolji, Jalal, Adatia, Adil.

##### University of Alberta, EDMONTON, AB

*Allergy, Asthma & Clinical Immunology* 2026, **22(2)**: 6

**Background** Asthma is the most common chronic condition among children, with a prevalence of 15% globally. The COVID-19 pandemic significantly impacted the healthcare utilization of children with asthma and seasonal trends of respiratory viruses. The impact of the COVID-19 pandemic on seasonal changes in emergency department (ED) presentations and emergency medical services (EMS) utilization for asthma exacerbations is not well studied.

**Methods** We conducted a retrospective study in children (ages 2–17) to examine the seasonality of ED visits for asthma and volume of ED visits arriving via EMS, from 2011 to 2024. The proportion of ED visits occurring within each month for asthma was tabulated according to age and sex from 2011 to 2024. The mean number of ED visits for normalized for population size arriving via EMS by age group and sex was also calculated. Data were collected from the Government of Alberta Interactive Health Data Application.

**Results** There were 61,500 ED presentations and 7,089 admissions for pediatric asthma from 2011–2024. In the pre-pandemic period (2011–2019), September accounted for the largest proportion of ED visits each year (range: 0.132–0.375) but not in 2020 (0.029–0.198). Seasonal patterns re-emerged in 2021 (0.003–0.306), with the September spike returning clearly by 2022 (0.117–0.365). Mean daily EMS visits per million persons was highest in 2022 at 5.076 and lowest in 2020 at 0.703. The highest EMS utilization for asthma was consistently observed in males aged 2–5 across all study years.

**Conclusions** The COVID-19 pandemic disrupted the September spike in pediatric asthma ED utilization in 2020 and 2021. There was a sharp decline in EMS utilization in 2020. EMS utilization rebounded in 2021 and has since remained at levels similar to or higher than pre-pandemic levels.

## 7

### Enhancing safety and access of sublingual immunotherapy: a quality improvement review of home-based grastek restart

#### Huan, Peter W.^1^, Morris, Emily G.^1^, Tang, Xinxin^1^, Nazarali, Samina^1^, Thabit, Lina^1^, Hircock, Caroline M.^2^, Kim, Harold L.^1, 3^

##### ^1^Western University, London, ON, ^2^Harvard T.H Chan School of Public Health, Boston, MA, USA, ^3^McMaster University, Hamilton, ON

*Allergy, Asthma & Clinical Immunology* 2026, **22(2)**: 7

**Background** The initial dose of Sublingual Immunotherapy (SLIT) carries a small risk of systemic allergic reactions. Current guidelines recommend initiation of SLIT in a medically supervised setting with a 30-min observation period. The suspension of non-essential aeroallergen immunotherapy during the COVID-19 pandemic led to the implementation of at-home reinitiation protocols. The objective of this study is to evaluate the safety of at-home reinitiation of Grastek in patients who had previously completed at least one prior season of therapy without significant adverse events.

**Methods** As part of a quality improvement initiative, 494 charts of grass-sensitized patients seen at a single outpatient allergy clinic between 2014 and 2025 were reviewed. Patients aged ≥ 5 years who had completed at least one prior season of Grastek and restarted in the home setting were included. Exclusions were year-round Grastek use, non-adherence (< 4 doses/week), or in-office first-dose administration each season. Adverse events were categorized using the World Allergy Organization (WAO) Updated Grading System based on self-reported symptoms.

**Results** Overall, 67 patients, including 35 females (52.2%) restarted their first dose of Grastek in the home setting. The median age at the start of Grastek therapy was 25 years (range:6–72). No patient reported any Grade 3–5 reactions after the first dose at home, required interventions or activated emergency medical service. Six patients (9.0%) reported the experience of a single system of Grade 1 localized oral discomfort occurring days to weeks after reinitiation. Three patients (4.5%) discontinued therapy despite prior tolerance to Grastek.

**Conclusions** Home-based reinitiation of Grastek in previously tolerant patients was safe, with no reactions meeting WAO anaphylaxis criteria. These findings contribute another real-world datapoint in support of the CSACI-endorsed approaches to home-based SLIT reintroduction implemented during the COVID-19 pandemic. However, larger and multi-center studies are needed to further evaluate safety and inform future guideline development.

## 8

### Improving safety and consistency in SCIT: a multi-site quality improvement initiative

#### Almaini, Mustafa, La, Kevin, Alkabra, Luna, Patag, Allyson, George, Candy.

##### ACIR, Mississauga, ON

*Allergy, Asthma & Clinical Immunology* 2026, **22(2)**: 8

**Background** Subcutaneous immunotherapy (SCIT) describes a form of treatment intended to increase tolerability to allergens and provide symptomatic relief to hypersensitivities such as allergic rhinoconjunctivitis. Although extensive research has shown that SCIT is effective in managing reactions to allergens,there is little data on therapy accessibility and adherence, therefore the goal of this paper is to dive deeper looking into SCIT accessibility, initiation, and adherence. It is hypothesized that the most common reason for SCIT discontinuation is related to adverse reactions, whereas the most significant contributor to treatment rejection is inconvenience.

**Methods** A retrospective review of the charts of all patients eligible for SCIT in a private allergy clinic in Ontario, Canada from January 2022 to June 6, 2025 was performed. Retrospective chart review was performed using Electronic Medical Records.The 331 patients included in the study were divided into 3 groups consisting of those who did not initiate treatment, those who discontinued treatment, and those who are currently active in the treatment. Various outcome measures were analyzed and grouped into 3 categories: treatment accessibility and initiation, treatment adherence, and treatment effectiveness.

**Results** Data analysis showed that of the 331 eligible patients, 81.21% proceeded with SCIT, with 7.43% having insurance coverage (including Ontario Drug Benefit, Trillium, ODSP), compared to 63.57% that had out-of-pocket coverage and the remaining 29.00% with Ontario Health Insurance Plan (OHIP). Lack of follow-up response (55.3%) and cost-related barriers (4.1%) were listed as the primary deterrents for starting treatment.Failure to reach patients despite multiple contact attempts is the leading cause for treatment discontinuation. Additionally, treatment effectiveness was reported through changes in severity grading scale.The majority of these adherent patients were polysensitized.

**Conclusions** This paper emphasizes the value of recognizing and understanding the therapeutic efficacy of SCIT from a clinical point of view and highlights the need to consider socioeconomic factors when prescribing SCIT.

## 9

### Assessing the impact of nasal sampling on TSLP, CMA1 and TPSAB1 gene expression in comorbid allergic rhinitis and asthma before and after controlled allergen exposure

#### Spicer, Sophie^1, 2^, Davis, Abigail^1, 2^, Hossenbaccus, Lubnaa^1, 2^, Ellis, Anne K.^1, 2^

##### ^1^Department of Medicine, Queen’s University, Kingston, ON, ^2^Allergy Research Unit, Kingston Health Sciences Centre (KHSC) – KGH Site, Kingston, ON

*Allergy, Asthma & Clinical Immunology* 2026, **22(2)**: 9

**Background** Nasal brush sampling offers a minimally invasive approach to studying gene expression of key mediators of allergic rhinitis (AR) and asthma including thymic stromal lymphopoietin (*TSLP)*, chymase (*CMA1)*, and tryptase (*TPSAB1),* but few studies have examined post-Nasal Allergen Challenge (NAC) gene expression changes in the comorbid population. Additionally, clinical studies investigating allergic diseases often require the utilization of several sampling techniques, however, little is known about how successive sampling impacts gene expression levels in the samples.

**Methods** This study analyzed nasal brush samples from both nostrils of 11 participants (6 non-allergic, 5 allergic), collected after a nasal lavage in one nostril and a nasal sponge in the other, before and 6-h after NAC. RNA from nasal brush samples was isolated and underwent a DNA digestion. The samples were then reverse transcribed and amplified via quantitative PCR to determine the gene expression of *TSLP*, *CMA1*, and *TPSAB1* relative to the reference gene *ubiquitin C* (*UBC*). Agreement between nasal brush samples following successive sampling was assessed with Bland–Altman analysis, and changes in relative gene expression after NAC were evaluated with a Wilcoxon signed-rank test (threshold of significance of 0.05).

**Results** There were minimal differences in gene expression levels from nasal brush samples regardless of pre-sampling by nasal sponge or lavage (bias = -0.2358, 95% CI: -26.80, 26.33). No statistically significant changes in *TSLP*, *CMA1*, or *TPSAB1* were observed 6-h post-NAC for both allergic and non-allergic participants.

**Conclusions** These results demonstrate preliminary reliability and consistency of successive nasal sampling, suggesting flexibility in nasal sampling protocols and enabling analysis of different components of the allergic response at the same time point. The lack of significant post-NAC differences may reflect limited power and the need for additional timepoints and protein-level analyses, which may be feasible given the minimal changes observed with successive nasal sampling.

## 10

### Real-world dupilumab effectiveness through 18 months in patients with CRSwNP and coexisting asthma: results from the global AROMA registry

#### Buchheit, Kathleen M.^1^, Heffler, Enrico^2^, Fujieda, Shigeharu^3^, Wagenmann, Martin^4^, Vozza, Nicolas^5^, Xia, Changming^6^, deMarchi, Sabina^6^, Corbett, Mark^7^, Radwan, Amr^8^

##### ^1^AERD Center, Division of Allergy and Clinical Immunology, Brigham and Women’s Hospital, Harvard Medical School, Boston, MA, USA, ^2^Personalized Medicine, Asthma and Allergy, Humanitas Research Hospital, Milan, Italy, ^3^Department of Otorhinolaryngology, Head and Neck Surgery, University of Fukui, Fukui, Japan, ^4^Department of Otorhinolaryngology, Düsseldorf University Hospital (UKD), Düsseldorf, North Rhine-Westphalia, Germany, ^5^Sanofi, Toronto, ON, ^6^Regeneron Pharmaceuticals Inc., Tarrytown, NY, USA, ^7^Sanofi, Morristown, NJ, USA, 8. Regeneron Pharmaceuticals Inc., Uxbridge, United Kingdom

*Allergy, Asthma & Clinical Immunology* 2026, **22(2)**: 10

**Background** The symptoms and health-related quality of life burden of chronic rhinosinusitis with nasal polyps (CRSwNP) are increased in patients with coexisting asthma. Dupilumab is efficacious for both CRSwNP and asthma, but real-world evidence in patients with coexisting disease is limited.

**Methods** The global, phase 4 AROMA registry study (NCT04959448) is following adults initiating dupilumab for CRSwNP to collect data on long-term effectiveness of dupilumab through change in patient-reported outcomes and health-related quality of life. AROMA is a real-world study being conducted in the USA, Canada, Germany, Italy, Japan, and the Netherlands. Results to 18 months are reported.

**Results** Of 691 CRSwNP dupilumab-treated patients, 478 (69.2%) had coexisting asthma; median age at asthma diagnosis was 37 years (IQR 24–48). A total of 163 patients reported one or more severe asthma exacerbations in the year before initiating dupilumab (mean 3.1 exacerbations, SD 5.58), and 31 patients reported a mean (SD) 2.4 (3.68) cumulative hospitalization days to treat severe asthma exacerbations. Asthma‑specific outcomes improved progressively: at baseline, 6, 12, and 18 months, mean (SD) ACQ-6 scores (scale 0–6) were 1.4 (1.20), 0.5 (0.65), 0.4 (0.62), and 0.3 (0.46), respectively; mini-AQLQ scores (scale 1–7) were 5.1 (1.29), 6.2 (0.82), 6.3 (0.84), and 6.5 (0.70), respectively; and FeNO (ppb) was 62.2 (66.88), 23.5 (14.58), 23.0 (14.95), and 25.1 (8.26), respectively. CRSwNP outcomes also improved progressively in patients with coexisting asthma: at baseline, 3, 12, and 18 months, mean (SD) nasal congestion scores (range 0–3) were 1.8 (0.85), 0.9 (0.74), 0.7 (0.74), and 0.6 (0.65), respectively; loss of smell scores (range 0–3) were 2.3 (1.01), 1.2 (1.04), 1.0 (1.03), and 0.9 (1.01), respectively; and SNOT-22 scores (range 0–110) were 46.4 (20.67), 21.1 (15.60), 16.0 (12.98), and 15.5 (13.63), respectively.

**Conclusions** Dupilumab demonstrates long-term effectiveness in patients with CRSwNP and coexisting asthma in real-world clinical practice.

#### 11

### The role of CD8 + T cells in severe adult asthma

#### Chen, Zi Shan^1, 2^, Emami Fard, Nahal^1, 3^, Ju, Xiaotian^1^, Huang, Chynna^3^, Satia, Imran^1, 2, 3^, O’Byrne, Paul^1, 2^, Mukherjee, Manali^2, 3^, Ho, Terence^2, 3^, Nair, Parameswaran^2, 3^, Sehmi, Roma^1, 2, 3^

##### ^1^Respiratory Research Group, Department of Medicine, McMaster University, Hamilton, ON, ^2^Honours Health Sciences Program, McMaster University, Hamilton, ON, ^3^Firestone Institute for Respiratory Health, St. Joseph’s Healthcare, Hamilton, ON

*Allergy, Asthma & Clinical Immunology* 2026, **22(2)**: 11

**Background** Asthma exacerbations accelerate lung function decline and disease burden, especially in prednisone-dependent severe asthma (SA) [1]. While Group 2 innate lymphoid cells (ILC2), and to a lesser extent, CD4 + T cells, are established contributors to T2 driven airway inflammation, new evidence suggests that CD8 + cytotoxic T cells (Tc) may also play a role [2,3]. This study enumerated airway levels of total Tc cells and changes in Tc phenotype subsets in SA when stable and at an exacerbation event.

**Methods** Sputum from consenting non-smoking SA patients [1] on high-dose corticosteroids ± T2 biologics was collected when stable (n = 19) and at an exacerbation (n = 16) event (defined as worsening symptoms requiring treatment adjustment). Sputum extracted cells were analyzed by flow cytometry and statistical comparisons were conducted using Mann–Whitney tests with Dunn’s post hoc analyses.

**Results** When stable, CD8 + T cells (Lin + CD45 + FcεR1 + CD8 +) were significantly greater (p < 0.0001) than ILC2s (Lin-CD45 + CD127 + CRTH2 +). We then phenotyped CD8 + T cells as Tc1, Tc2 and Tc17 subgroups within sputum. When stable versus exacerbation visits were compared, there were no significant differences in total CD8 + T cells but significant increases (p < 0.05) were found in Tc1 (IFNγ + CD8 +), Tc2 (CRTH2 + CD8 +) and a trend for increase in Tc17 (RORγt + CD8 + ;C-kit + CD8 +).

**Conclusions** This study shows that in SA, CD8 + T cells are significantly more prevalent than ILC2 at stable. Although CD8 + T cell proportions remained the same, phenotype-specific increases were observed for Tc1 and Tc2 cell populations in SA exacerbations. How these changes relate to triggers of exacerbation, the resultant airway inflammatory profile, the effect of T2 biologics and the factors that drive these changes require further assessments.

**References**

1. Song W-J, Lee J-H, Kang Y, Joung WJ, Chung KF. Future risks in patients with severe asthma. *Allergy Asthma Immunol Res*. 2019;11(6):763. 10.4168/aair.2019.11.6.763
2. Bryant N, Muehling LM. T-cell responses in asthma exacerbations. *Ann Allergy Asthma Immunol*. 2022 Dec;129(6):709–18. 10.1016/j.anai.2022.07.027
3. Hamada H, Garcia-Hernandez M de, Reome JB, Misra SK, Strutt TM, McKinstry KK, et al. TC17, a unique subset of CD8 T cells that can protect against Lethal Influenza Challenge. *J. Immunol.*. 2009 Mar 15;182(6):3469–81. 10.4049/jimmunol.0801814

## Allied Health

## 12

### An evaluation of resources for managing food allergy and food insecurity

#### Harbottle, Zoe^1, 2^, Golding, Michael A.^1, 2^, Wiens, Justina^1, 2^, Snarr, Phillip^1, 2^, Protudjer, Jennifer L.^1, 2, 3^

##### ^1^Department of Pediatrics and Child Health, University of Manitoba, Winnipeg, MB, ^2^Children’s Hospital Research Institute of Manitoba, Winnipeg, MB, ^3^Institute of Environmental Medicine, Karolinska Institutet, Stockholm, Sweden

*Allergy, Asthma & Clinical Immunology* 2026, **22(2)**: 12

**Background** Families managing food allergy face disproportionately higher food costs, compared to families without food allergy, placing them at higher risk of experiencing food insecurity. In light of rising food costs, allergists and allied healthcare professionals are likely to support an increasing number of patients concerned about food security. Yet, there is a lack of information on how healthcare professionals can support these families and appropriate patient-focused resources. Therefore, we aimed to evaluate newly developed resources on food allergy and food insecurity designed for families managing food allergy and for healthcare professionals.

**Methods** As part of an overarching integrated knowledge translation project, four resources were developed: two patient-focused resources and two healthcare professional-specific resources. Between October 2024 and April 2025, caregivers of a child with food allergy and healthcare professionals working with patients with food allergy were recruited via newsletters, food banks, and social media. Participants were asked to evaluate the resources most applicable to them for relevance, comprehension, and overall appearance using Likert scale questions and open-ended questions. Quantitative data was analyzed descriptively using Excel. Open-ended questions were analyzed thematically.

**Results** Overall, 18 caregivers and 11 healthcare professionals, including one allergist and 10 registered dietitians, participated. 94.4% of caregivers found the information easy to understand and applicable to their daily life. 86.4% of healthcare professionals found the resources relevant to their practice. Both caregivers and healthcare professionals reported that the resources should be shorter and more visually appealing.

**Conclusions** Overall, participants indicated that the resources were relevant and provided useful information but need to be refined to ensure effective communication of information. Our findings demonstrate that these resources will be a beneficial tool for both caregivers of children with food allergy and healthcare professionals working in food allergy to support informed decision making and conversations surrounding food insecurity.

## 13

### Examination of pulse wave velocity associations with asthma, spirometry, and methacholine challenge in the Canadian Asthma Primary Prevention Study

#### Houlbrook, Doug^1^, Deluz, Scarlet^1^, Johnson-Audu, Joanna^1^, Chan, Edmond^2^, Becker, Allan B.^1, 3^, McGavock, Jon^1, 4^, Simons, Elinor^1, 3^

##### ^1^Children’s Hospital Research Institute of Manitoba, Winnipeg, MB, ^2^Division of Allergy, Department of Pediatrics, University of British Columbia and British Columbia Children’s Hospital Research Institute, Vancouver, BC, ^3^Section of Allergy & Immunology, Department of Pediatrics & Child Health, University of Manitoba, Winnipeg, MB, ^4^Department of Pediatrics & Child Health, University of Manitoba, Winnipeg, MB

*Allergy, Asthma & Clinical Immunology* 2026, **22(2)**: 13

**Background** Previous asthma diagnosis has been demonstrated to be a risk factor for adult cardiovascular disease, measured using pulse wave velocity (PWV). Childhood and adolescent characteristics have been increasingly recognized as possible predictors of chronic diseases. We examined associations between asthma and PWV in the teen years in The Canadian Asthma Primary Prevention Study (CAPPS), a birth cohort of children at high risk for allergy and asthma.

**Methods** At age 15 years ± 6 months, CAPPS participants underwent a clinical assessment to evaluate for asthma, spirometry, methacholine challenge (MCH) and PWV. We used Mann–Whitney U test to examine PWV (m/s) for children with and without clinical asthma, forced expiratory volume in 1 s (FEV1) above and below 80%, and provocative challenge causing a 20% decrease in FEV1 (PC20) above and below 8 mg/mL.

**Results** Of the 105 participants, 47 females (45%) and 58 males (55%) completed all measures. PWV (median 6.35, interquartile range 5.8–6.7) was comparable for children with (6.4, 5.6–6.8) and without (6.3, 5.9–6.7) a clinical diagnosis of asthma (p = 0.43) and children with an FEV1 at or above 80% (6.3, 5.9–6.7) compared with an FEV1 below 80% (6.3, 5.9–6.4, p = 0.37). PWV was significantly different for children with a PC20 above 8 mg/mL (6.4, 6.1–6.7) compared with a PC20 at or below 8 mg/mL (6.2, 5.6–6.3, p = 0.04).

**Conclusions** Further evaluation of the associations between PWV and asthma, spirometry, and MCH is warranted, including examining the clinical significance of the observed association between PWV and MCH, and possible confounding or interaction by gender, SES, obesity or overweight, atopy, and age of asthma diagnosis.

## 14

### Resident experiences with strategic approaches to the learning environment within small postgraduate residency programs at Canadian research-intensive universities

#### Sparanese, Sydney^1^, Lin-Yang, Rachel C.^2^, Rubin, Tamar S.^3^, Connors, Lori^4^, Hildebrand, Kyla J.^5^

##### ^1^Department of Medicine, Faculty of Medicine, University of British Columbia, Vancouver, BC, ^2^Institute for the Scholarship of Teaching and Learning, University of British Columbia, Vancouver, BC, ^3^Section of Pediatric Clinical Immunology and Allergy, Department of Pediatrics & Child Health, University of Manitoba, Winnipeg, MB, ^4^Department of Medicine, Dalhousie University, Halifax, BC, ^5^Division of Immunology, Department of Pediatrics, University of British Columbia, Vancouver, BC

*Allergy, Asthma & Clinical Immunology* 2026, **22(2)**: 14

**Background** Medical education accreditation standards require residency training programs to have a comprehensive curriculum plan specific to the discipline that encompasses medical expert and non-medical expert physician competencies. To meet this requirement, several Canadian Clinical Immunology and Allergy (CIA) residency programs participate in a distributed academic half-day (DAHD). There is limited literature on evidence-based practices in distributed medical education, particularly with the use of a virtual platform. This study explored three key questions in the virtual learning environment for CIA trainees at Canadian research-intensive universities:

1. What contextual factors influence the learning environment in distributed postgraduate medical education?
2. What are the current best practices in subspecialty distributed medical education?
3. What institutional supports enable participation in a distributed academic half day?

**Methods** The UBC Behavioral Research Ethics Board approved this study. A qualitative study design using appreciative inquiry within an interpretive description framework was applied. Data was gathered through semi-structured focus groups and interviews with resident physicians participating in the DAHD. We used an iterative coding process to identify key themes.

**Results** Three key themes emerged:

1. **High-Yield Didactics from Content Experts** – Residents valued lectures from national experts, particularly in areas lacking local expertise. These sessions offered clinical nuance and encouraged staff participation, enhancing cross-country engagement.
2. **Peer Connectedness Enhances Opportunities** – The distributed format enabled residents to learn about electives and mentorship opportunities at other institutions, expanding access to training and support.
3. **Logistical Challenges** – Scheduling across time zones posed barriers to consistent attendance.

**Conclusions** The distributed learning environment fosters access to high-quality didactics through national content experts, peer-to-peer networking, and early mentorship for resident physicians in small subspecialty training programs. These features enhance the educational experience and support resident development. However, logistical challenges, including scheduling and competing demands on residents, may affect participation and impact the learning experience.

## 15

### The development of Pediatric Allergy Outreach Clinics in northern Manitoba communities

#### St-Vincent, Jo-Anne^1^, North, Kathleen^3^, Filuk, Shauna^1^, Ross, Nancy^1^, Kirkness, Shelley^4^, Johnstone, Lenore^1^, Dawyduk, Brenda^5^, Doucette, Caroline^1^, Rubin, Tamar^2^, Deane, Shannon^2^, Loewen, Keely^2^, Simons, Elinor^2^

##### ^1^The Children’s Allergy and Asthma Education Centre, Winnipeg, MB, ^2^The Children’s Allergy and Asthma Education Centre and Section of Allergy and Immunology, Department of Pediatrics and Child Health, University of Manitoba, Winnipeg, MB, ^3^Keewaatinohk Inniniw Minoayawin (KIM), Winnipeg, MB, ^4^Opaskwayak Cree Nation, The Pas, MB, ^5^Pediatrics Shared Health, Northern Regional Health Authority, Thompson, MB

*Allergy, Asthma & Clinical Immunology* 2026, **22(2)**: 15

**Background** Asthma, food allergies, and eczema have an enormous impact on Manitoba children. Our goal was to build partnerships with key players in the healthcare system of northern and remote communities to better understand their unique characteristics and to improve their access to Pediatric Allergy care.

**Methods** We examined Pediatric Allergy and Education referrals from northern Manitoba communities from 2021–2024. Nurse Educators developed partnerships with staff affiliated with Jordan’s Principle and consulted Manitoba First Nations Tribal Council, Keewaatinohk Inniniw Minoayawin (KIM), nursing stations, health centres, and consulting pediatricians providing service in northern Manitoba. Nurse Educators travelled to northern communities to learn about their needs. Funding sources were explored to cover travel and hotels. Referrals were reviewed to identify specific patient and family needs, local resources, and catchment areas.

**Results** From 2021–2024, Pediatric Allergy referrals from northern Manitoba communities increased from 222 in 2021 to 311 in 2024; Education referrals increased from 144 in 2022 to 169 in 2024. Missed in-person appointments in Winnipeg increased from 25% in 2021 to 42% in 2024 for Pediatric Allergy, and from 21% in 2022 to 35% in 2024 for Education. Pediatric Allergy clinics were offered in the nursing station, health centre, or hospital of 6 communities. Clinics were staffed by a Pediatric Allergist and two Nurse Educators. Children were seen for initial consultation, follow-up care, and/or Education. Jordan’s Principle and local health centre staff played key roles in ensuring that families were able to attend. Educational opportunities were offered to staff. Pediatric Allergy clinics are planned in these communities every 6–12 months.

**Conclusions** Distance from referral centers presents significant barriers to Pediatric Allergy care, and travelling to northern Manitoba communities reduces these barriers and improves patient and family access. Partnerships with the northern communities are crucial in the ongoing development and success of this outreach initiative.

## Food Allergy / Anaphylaxis

## 16

### Eosinophilic esophagitis-like symptoms in preschool oral immunotherapy

#### Kim, Min Jung^1^, Chan, Edmond S.^2, 3^, Erdle, Stephanie C.^2, 3^, Avinashi, Vishal^3, 4^, Yeung, Joanne^2, 3^, Kapur, Sandeep^5^, Cameron, Scott B.^2, 6^, Vander Leek, Timothy K.^7^, McHenry, Mary^5^, Carr, Stuart^7, 8^, Cook, Victoria^2, 6^, Leo, Sara^2, 9^, Soller, Lianne^2, 3^

##### ^1^Department of Pediatrics, Faculty of Medicine, University of Western Ontario, London, ON, ^2^Division of Allergy, Department of Pediatrics, Faculty of Medicine, University of British Columbia, Vancouver, BC, ^3^British Columbia Children’s Hospital, Vancouver, BC, ^4^Division of Gastroenterology, Department of Pediatrics, Faculty of Medicine, University of British Columbia, Vancouver, BC, ^5^Division of Allergy, Department of Pediatrics, Dalhousie University, IWK Health Centre, Halifax, NS, ^6^Community Allergy Clinic, Victoria, BC, ^7^Pediatric Allergy & Asthma, Department of Pediatrics, University of Alberta, Edmonton, AB, ^8^Snö Wellness Clinic, Abu Dhabi, United Arab Emirates, ^9^West Coast Allergy and Immunology Clinic, Vancouver, BC

*Allergy, Asthma & Clinical Immunology* 2026, **22(2)**: 16

**Background** Eosinophilic esophagitis (EoE) has been previously described as a potential complication of oral immunotherapy (OIT), with a reported incidence of 2.7–3.2%.[1,2] The development of EoE-like symptoms is often an indication for pausing or discontinuing OIT; however, there is no clear consensus regarding the management of such patients. Furthermore, prior studies have largely described OIT-induced EoE in older populations. Thus, we sought to describe the incidence of EoE-like symptoms and outcomes for preschool OIT.

**Methods** Patients with new EoE-like symptoms during OIT were identified from a cohort of pediatric patients enrolled in the UBC REB approved Food Allergy Immunotherapy Registry, which collected prospective data from centres across five Canadian cities.

**Results** Of 2188 patients undergoing OIT between 2017 to 2024, 23 (1.1%) developed new EoE-like symptoms during build-up or maintenance, most commonly vomiting and abdominal pain. The median age at initiation of OIT for patients who developed EoE-like symptoms was 46 months [IQR 34, 78]. Only three patients underwent endoscopy for evaluation, all with normal biopsy. Thirteen patients (56.5%) discontinued OIT due to EoE-like symptoms, whereas the remaining ten continued on the same dose (17.4%), went back to a lower dose (17.4%), or took a break from OIT prior to restarting (8.7%). Of those who continued on OIT, two were started on PPI therapy and all ten eventually achieved maintenance. Five patients completed and successfully passed an exit oral food challenge following a period of maintenance, and the remaining five patients are currently awaiting exit OFCs.

**Conclusions** In contrast to prior studies of older children describing OIT-induced EoE, we found low rates of EoE-like symptoms in our preschool population, with no cases of new endoscopically proven EoE during OIT. The few preschoolers on OIT with new onset EoE-like symptoms rarely underwent endoscopy. Furthermore, those who remained on OIT were able to successfully achieve desensitization.

**References**

1. Lucendo AJ, Arias Á, Tenias JM. **Relation between eosinophilic esophagitis and oral immunotherapy for food allergy: a systematic review with meta-analysis**. *Ann Allergy Asthma Immunol*. 2014; **113**:624–629.
2. Dunlop JH, Keet CA, Mudd K, et al. **Long-term follow-up after baked milk introduction**. *J Allergy Clin Immunol Pract*. 2018; **6**:1699–1704.

## 17

### A novel program for food-allergy anxiety in Canada: The thriving families program at BC Children’s Hospital Allergy Clinic

#### Lee, Zerlyn H.^1^, Tong, Jennifer Y.^2, 3^, Burns, Kyle D.^4^, Protudjer, Jennifer L.^5, 6, 7^, Golding, Michael A.^5, 6^, Mak, Raymond^2, 8^, Soller, Lianne^2, 8^, Chan, Edmond S.^2, 8^

##### ^1^General Pediatric Residency Training Program, Department of Pediatrics, Faculty of Medicine, The University of British Columbia, Vancouver, BC, ^2^Division of Allergy, Department of Pediatrics, Faculty of Medicine, The University of British Columbia, Vancouver, BC, ^3^Department of Family Practice, Faculty of Medicine, The University of British Columbia, Vancouver, BC, ^4^Department of Psychiatry, Faculty of Medicine, The University of British Columbia, Vancouver, BC, ^5^Department of Pediatrics and Child Health, Max Rady College of Medicine, Rady Faculty of Health Sciences, University of Manitoba, Winnipeg, MB, ^6^Children’s Hospital Research Institute of Manitoba, Winnipeg, BC, ^7^Department of Food and Human Nutritional Sciences, Faculty of Agricultural and Food Sciences, University of Manitoba, Winnipeg, MB, ^8^BC Children’s Hospital Research Institute, Vancouver, BC

*Allergy, Asthma & Clinical Immunology* 2026, **22(2)**: 17

**Background** Food allergy anxiety (FAA) often contributes negatively to allergy management and outcomes. [1–5] To address FAA, the British Columbia Children’s Hospital Allergy Clinic is piloting the Thriving Families Program (TFP)—a novel, primarily virtual program incorporating Dialectical Behaviour Therapy (DBT) skills within a Cognitive-Behavioral Therapy (CBT)-based intervention delivered by a family physician with psychotherapy training.

**Methods** For this mixed-methods quality improvement project, data was gathered from families referred to TFP from April 2024 to April 2025. Retrospective chart review yielded quantitative data on demographics and encounter types, with content analysis yielding qualitative data on positive outcomes, and mixed data on family concerns and outcomes for discharged families.

**Results** From April 2024 to April 2025, 74 families were seen in the TFP, the majority of whom were urban residents (n = 62). Most patients had multiple food allergies (n = 65), of which the most common allergens were tree nuts (n = 48; 77.4%) and peanut (n = 40; 64.5%). Many families participated in group sessions (n = 35; 47%) in addition to intake or individual sessions. The most common concern was high FAA in the child/youth (n = 60), followed by high parental FAA (n = 55) and challenges related to food allergy immunotherapy (n = 53). Positive outcome themes include decreased FAA, support for diagnostic procedures and food immunotherapy, increased confidence to try new foods and experiences, support with accessing in-person psychology, and interdisciplinary transition to adult care. Of the 17 families discharged, the majority (n = 14) had at least one positive outcome identified; other families had not completed recommended sessions (n = 3).

**Conclusions** Our new in-house psychosocial program for FAA can improve FAA and treatment outcomes. Study limitations include data being limited to physician documentation, resulting in insufficient data for analysis of negative outcomes or quantitative outcomes of families still in care. Outcome themes identified will guide future studies eliciting family feedback and evaluating final outcomes.

**References**

1. Casale TB, Warren C, Gupta S, Schuldt R, Wang R, Iqbal A, Seetasith A, & Gupta R. **The mental health burden of food allergies: Insights from patients and their caregivers from the Food Allergy Research and Education (FARE) patient registry**. *World Allergy Organization Journal* 2024, **17**(4): 100,891. 10.1016/j.waojou.2024.100891.
2. Xie Q, Dai X, Tang X, Chan CHY. **Risk of mental disorders in children and adolescents with atopic dermatitis: A systematic review and meta-analysis.** *Frontiers in Psychology* 2019, **10.** 10.3389/fpsyg.2019.01773.
3. Feng C, Kim J. **Beyond avoidance: The psychosocial impact of food allergies.** *Clinical Reviews in Allergy & Immunology* 2018, **57**(10): 74-82. 10.1007/s12016-018-8708-x.
4. Polloni L, Muraro A, Bonaguro R, Toniolo A, Ballin A, Guarnaccia A, Lazzarotto F. **Psychological needs and support among patients and families undergoing food oral immunotherapy.** *Clinical and Translational Allergy* 2022, **12**(2). 10.1002/clt2.12078.
5. Dahlsgaard KK, Lewis MO, Seprgel, JM. **Cognitive-behavioural intervention for anxiety associated with food allergy in a clinical sample of children.** *Annals of Allergy, Asthma, & Immunology* 2023, **130**(1): 100-105. 10.1016/j.anai.2002.09.021.

## 18

### An update on the CSACI Food Allergy Educator Program after 4 cohorts

#### Protudjer, Jennifer L.^1^, Mack, Douglas P.^2^, Connors, Lori^3^, Lidington, Jasmin^4^, Kim, Harold^5^

##### ^1^University of Manitoba, Winnipeg, MB, ^2^McMaster University, Hamilton, ON, ^3^Dalhousie University, Halifax, NS, ^4^Canadian Society of Allergy and Clinical Immunology, Ottawa, ON, ^5^University of Western Ontario, Kitchener, ON

*Allergy, Asthma & Clinical Immunology* 2026, **22(2)**: 18

**Background** In response to the need for continued professional development training in allergy, the Canadian Society of Allergy and Clinical Immunology (CSACI) introduced a food allergy educator program for physicians and allied health professionals working in allergy in Fall 2023. In this submission, we present an update on the first four cohorts of the foundational course.

**Methods** Starting in Fall 2023, and every Spring and Fall since, we have offered an 8-week foundational course in food allergy, the content of which was delivered by world leaders in food allergy. This foundational course covered topics ranging from food allergy epidemiology, diagnosis and management, to the psychosocial burden of food allergy. At the time of enrollment, participants completed a pre-test; one week following the completion of the course, participants completed a post-test (passing grade 75%). Data were analysed descriptively with comparisons made using a paired t-test (Stata Version 17.0, College Station, TX). As an educational course, the University of Manitoba Research Ethics Board deemed research ethics board approval unnecessary subsequent to review of the course proposal and overview.

**Results** To date, 32 learners have taken the course. The collective mean pre-course test score was 72.5% (range 30.0%-96.0%). The corresponding post-test scores were 84.5% (range 65.0%-97.5%), consistent with a significant improvement in food allergy knowledge from the pre-test (p < 0.0001).

**Conclusions** In the first four cohorts of our needs-informed and evidence-based food allergy educator program, learners’ food allergy knowledge significantly improved. A foundational course will be offered again in Fall 2025. Those who have successfully completed the foundation course may apply to the advanced practice course in Spring 2026.

## 19

### Cashew desensitization in children: comparing clinical benefits and risks of three protocols

#### Rodrigues, Catarina^1^, Demean Loghin, Alino^2^, NoorSaeed, Sundus^3^, Ton That, Alexandre^1^, Najem, Joseph^1^, Kaouache, Mohammed^5^, Al Ali, Adnan^4^, Sigman, Karen^1^, McCusker, Christine^1^, Ben-Shoshan, Moshe^1^

##### ^1^McGill University, Montreal, QC, ^2^Université de Montreal, Montreal, QC, ^3^King Abdulaziz University, Jeddah, Saudi Arabia, ^4^United Arab Emirates University, Abu Dhabi, United Arab Emirates, ^5^Prince Sultan University, Riyadh, Saudi Arabia

*Allergy, Asthma & Clinical Immunology* 2026, **22(2)**: 19

**Background** Tree nut allergy affects more than 1% of Canadians. Cashew is a major culprit of tree nut allergy and anaphylaxis. This prospective cohort study conducted at the Montreal Children’s hospital between 2021–2025 studied the benefits and risks associated with three different oral immunotherapy (OIT) protocols: cashew milk (CM), cashew muffin (CMF) and crushed cashews (CC).

**Methods** Participants included children with a history of an IgE mediated reaction to cashew and a positive skin prick test who consented to OIT. Choice of protocol was determined by the treating allergist. Maintenance dose was 250 mg cashew protein in CC or butter form. Primary analysis compared the risk of anaphylactic reaction (AAR) and allergic reaction (AR) using a proportional odds ordinal logistic regression model in both per-protocol (PP) and intention-to-treat (ITT) groups. Multiple imputation was used for missing outcomes in the ITT cohort.

**Results** 99 patients were recruited (61.6% males, median age 2.5 years (IQR 1.4–3.6)). History of eczema or asthma was present in 68.7% and 22.2% of patients respectively. CC accounted for 64.6% of protocols, followed by CM (22.2%) and CMF (13.1%). In the PP group (n = 69), the CC protocol was associated with 19.7 times higher odds of experiencing a more severe AR and AAR (95%CI_2.2–172.4,_ p = 0.007), and 6.4 times higher odds in the CMF protocol (p = 0.14), both comparing to CM. In the ITT group, 10.6-fold higher odds were observed when comparing CC to CM (95%CI_2.5–44.8_, p = 0.002). Finished protocols were observed in 93.8% of CC protocols, 90.9% of CM and 92.3% of CMF protocols.

**Conclusions** The protocol with the highest percentage of patients led to more severe reactions when compared to cashew milk. Effective end results with significantly lower risk of AR and AAR are observed with cashew milk protocols.

## 20

### The updated cross-Canada anaphylaxis registry (craft): demographics, triggers, co-factors and management

#### Ton That, Alexandre^1^, Joseph, Najem^1^, Abrams, Elissa^2^, Alqurashi, Waleed^3^, Chan, Edmond S.^4^, Chowdhury, Raisa^1^, Chowdhury, Raisa^1^, Ellis, Anne^5^, Gerdts, Jennifer^6^, Hazzazi, Khaled M^7^, Leek, Timothy Vander^8^, Lim, Rod^9^, Perron, Paul-Andre^10^, Protudjer, Jennifer LP^2^, Ben-Shoshan, Moshe^1^

##### ^1^McGill University, Montreal, QC, ^2^Children’s Hospital Research Institute of Manitoba, Winnipeg, MB, ^3^Department of Pediatrics and Emergency Medicine, Ottawa, ON, ^4^Division of Allergy, Department of Pediatrics, Vancouver, BC, ^5^Kingston Health Sciences Centre, Kingston, ON, ^6^Food Allergy Canada, Toronto, ON, ^7^King Faisal Specialist Hospital and Research Centre, Madinah, Saudi Arabia, ^8^University of Alberta, Edmonton, AB, ^9^Children’s Hospital at London Health Science Centre, London, ON, ^10^Bureau du coroner, Québec, QC

*Allergy, Asthma & Clinical Immunology* 2026, **22(2)**: 20

**Background** Anaphylaxis is a severe, potentially life-threatening allergic reaction. Most fatal cases are preventable. Yet, Canadian data on anaphylaxis fatalities remain scarce. This study aimed to describe the causes, contributing factors and management of fatal anaphylaxis in Canada.

**Methods** The Cross-Canada Registry for Anaphylaxis Fatalities (CRAFT) is the first national registry to systematically collect standardized data on fatal anaphylaxis. Variables included sociodemographic, triggers, clinical presentation, comorbidities and management. Data were collected through coroners’ reports, lay allergy associations, medical literature and social media. Anaphylaxis was classified as probable/unlikely anaphylaxis by a board-certified allergist based on the National Institute of Allergy and Infection Diseases criteria. Descriptive statistics were used to examine the data. The study was approved by McGill’s Research Ethics Board.

**Results** Between January 2020 and December 2024, 58 cases were obtained from Quebec (n = 15), Ontario (n = 30), Alberta (n = 2), Saskatchewan (n = 5), British Columbia (n = 4) and Nova Scotia (n = 2). Overall, 81.0%(n = 47) of cases were probable anaphylaxis. Median age was 46.5 years; 52.3% were male. Reaction locations included: home (35.7%), outdoors (23.8%), healthcare settings (19.0%), friend’s residence (11.9%) and others. Triggers included insect stings (55.3%), food ingestion (27.7%), intravenous (10.6%), and others (6.4%), with noted geographical differences. Insect stings were the leading cause across all provinces, whereas all pediatric fatalities were food-related (n = 6), with 50% caused by milk. The leading food trigger for adult was shellfish (33.3%). 59.6% had a reported known allergy. Epinephrine was administered in 56.3% of cases. Comorbidities were present in 61.0%, including asthma (21.9%) and cardiac conditions (17.1%). Alcohol/cannabis were reported in 9.8% and 4.9%, respectively.

**Conclusions** Overall, insect stings caused more anaphylaxis fatalities in Canada than food, contrasting earlier data. However, food was the sole trigger in children. In fatalities, pre-existing comorbidities were noted and about half did not receive epinephrine. These findings, underscore the need for better guidelines, policies and education.

## 21

### Evaluation of safety for home-based and partially virtually supervised oral immunotherapy in low-risk infants and preschoolers

#### Almanaseer, Ala^1^, Chan, Edmond S.^2, 3^, Soller, Lianne^2, 3^, Ye, LinLei^4^, Vander Leek, Timothy K.^5^, Mak, Raymond^2, 3^

##### ^1^Faculty of Medicine, University of British Columbia, Vancouver, BC, Canada., Vancouver, BC, ^2^Division of Allergy, Department of Pediatrics, Faculty of Medicine, University of British Columbia, Vancouver, BC, Canada., Vancouver, BC, ^3^British Columbia Children’s Hospital, Vancouver, BC, Canada, Vancouver, BC, ^4^Department of Pediatrics, University of Alberta, Edmonton, Alberta, Canada, Edmonton, AB, ^5^Division of Pediatric Allergy, Department of Pediatrics, University of Alberta, Edmonton, Alberta, Canada, Edmonton, AB

*Allergy, Asthma & Clinical Immunology* 2026, **22(2)**: 21

**Background** Oral Immunotherapy (OIT) has been shown to reduce anaphylaxis risk in IgE-mediated food allergies. Home-based OIT is an emerging model that allows low-risk patients to independently up-dose at home, reducing the need for clinic-based supervision. This approach may safely increase OIT accessibility, while also reducing waitlists and associated costs. This Quality Improvement study evaluated the safety and impact on resource utilization of two modified OIT models, including a fully home-based approach, and a combination of virtual supervision with independent home-based up-dosing.

**Methods** A retrospective chart review was conducted between April 1, 2024 and March 31, 2025, at BC Children’s Hospital in Vancouver, BC, Canada. Infants and Pre-schoolers who underwent home-based OIT or a combination of virtually supervised and home-based up-dosing were assessed for inclusion (N = 97). Although grade 4 severity was an exclusion criterion, there were no patients with baseline grade 4 reactions requiring exclusion.

**Results** Two patients (2.06%) across the two OIT models had a grade two reaction, with the remainder of patients having grade one or no reactions. Patients did not experience grade three or four reactions during OIT build-up. 39 patients (40%) achieved maintenance within the 12-month period, with an average visit reduction of 58% (4.6 visits compared to a traditional OIT approach of 11 visits).

**Conclusions** OIT delivered through a home-based or partially virtually supervised approach offers low-risk patients a safe alternative to conventional in-person up-dosing. Reducing the need for in-clinic supervision allows clinicians to spend more time at OIT follow-up visits to focus on critical primary prevention counselling, such as reinforcing the need for regular ingestion of other allergenic foods. Benefits of this alternative approach also include a reduction in the number of visits, while maintaining safety and expanding OIT access to families who may not be accommodated due to limited clinic capacity.

## 22

### Accelerating the build-up phase for fish oral immunotherapy in pediatric patients

#### Almanaseer, Ala^1^, Chan, Edmond S.^2, 3^, Mak, Raymond^2, 3^, Erdle, Stephanie^2, 3^, Wong, Tiffany^2, 3^, Soller, Lianne^2, 3^, Le Blanc, Vicky^2, 3^

##### ^1^Faculty of Medicine, University of British Columbia, Vancouver, BC, Canada., Vancouver, BC, ^2^Division of Allergy, Department of Pediatrics, Faculty of Medicine, University of British Columbia, Vancouver, BC, Canada., Vancouver, BC, ^3^British Columbia Children’s Hospital, Vancouver, BC, Canada, Vancouver, BC

*Allergy, Asthma & Clinical Immunology* 2026, **22(2)**: 22

**Background** Oral immunotherapy (OIT) is a safe and effective therapeutic option for peanut and tree nut allergies in preschool children.^1^ Although current literature on fish allergy is limited, some fish allergens are known to elicit reactions at higher thresholds.^2^ Given the estimated prevalence of fish allergy in 1.1% of Canadian children,^3^ incorporating higher eliciting doses of fish allergens may improve accessibility and decrease reliance on scarce and unpalatable fish powder.

**Methods** Children less than 18 years old with skin prick test (SPT) ≥ 3 mm or specific IgE (sIgE) level ≥ 0.35 kU/L, prior IgE-mediated fish reaction, or no ingestion history but sIgE level ≥ 5 kU/L or SPT ≥ 8 mm, underwent a threshold oral food challenge (OFC) to minimum 20 mg of fish protein. This Quality Improvement project was exempt from the UBC Research Ethics Board (REB) review.

**Results** Between January 2023 and April 2025, six children ages one to twelve completed a low-dose OFC to fish protein for OIT initiation. Five patients (83.3%) tolerated a minimum of 20 mg without reaction, and one patient (16.7%) had a mild reaction (facial urticaria). Two patients (33.3%) began their OIT with maintenance dosing following an OFC of minimum 300 mg fish protein. One patient (16.7%) experienced taste aversion, resulting in up-dosing delays. No anaphylactic reactions were reported.

**Conclusions** For pediatric patients undergoing fish OIT, they may begin with a supervised low-dose OFC of 20 mg prior to up-dosing to avoid unpalatable fish powder use and to improve accessibility.

**References**

1. Soller L, Abrams EM, Carr S, et al. First real-world safety analysis of preschool peanut oral immunotherapy. J Allergy Clin Immunol Pract. 2019 Dec;7(8):2759–2767.e5.
2. Houben GF, Baumert JL, Blom WM, Kruizinga AG, Meima MY, Remington BC, et al. Updated population minimal eliciting dose distributions for use in risk assessment of 14 priority food allergens. Food Chem Toxicol. 2020 May;139:111259. 10.1016/j.fct.2020.111259
3. Health Canada. Research related to the prevalence of food allergies and intolerances [Internet]. Ottawa (ON): Government of Canada; 2016 [cited 2025 Jun 11]. Available from: https://www.canada.ca/en/health-canada/services/food-nutrition/food-safety/food-allergies-intolerances/food-allergen-research-program/research-related-prevalence-food-allergies-intolerances.html

## 23

### Psychological impact of oral and sublingual immunotherapy in patients with food allergy: a scoping review

#### Andary, Catherine M.^1^, Zheng, Jiayue^2^, Kan-Hodgson, Michelle^3^, Abid, Humaira^4^, Ostermeyer, Britta^4^, Terpstra, Collin R.^5^, Olagunju, Andrew T.^4, 5, 6^

##### ^1^University of Toronto, Toronto, ON, ^2^University of British Columbia, Vancouver, BC, ^3^Dalhousie University, Halifax, NS, ^4^University of Oklahoma College of Medicine, Oklahoma City, OK, USA, ^5^McMaster University, Hamilton, ON, ^6^The University of Adelaide, Adelaide, SA, Australia

*Allergy, Asthma & Clinical Immunology* 2026, **22(2)**: 23

**Background** IgE-mediated food allergy affects over 3 million Canadians, leading to health and social burdens. Oral and sublingual immunotherapy (OIT and SLIT, respectively) have emerged as desensitization strategies for food allergy, with studies primarily investigating their clinical outcomes and adverse effects. However, the psychological impacts of OIT and SLIT remain underexplored. This scoping review examines psychological outcomes in patients on OIT and SLIT, including overall quality of life, anxiety, and stress.

**Methods** The scoping review follows Arksey and O’Malley’s framework, using search terms developed in collaboration with health science librarians. Databases searched include PsycINFO, Embase, MedLine, PMC, EBM Reviews, Cochrane Reviews, Web of Science, and Scopus. Original peer-reviewed articles published in all languages after 2010 were reviewed. Exclusion criteria included non-peer-reviewed studies and studies not involving food allergy. Data extraction was completed using Covidence in June 2025 by three reviewers blinded at both title and abstract screening and full-text screening. Screening conflicts were resolved by consensus discussion.

**Results** Peer-reviewed literature was searched in June 2025, which resulted in 1141 unique studies. 146 abstracts (12.8%) advanced to full text screening, yielding 62 (42.5%) articles for data extraction. Completion of data extraction is expected by August 2025, further examining the immunotherapy protocols used, patient demographic factors, and their impact on psychological outcomes. Initial analysis has highlighted that, while OIT and SLIT have a positive or negligible effect on quality of life in food allergy patients, patient and caregiver anxiety represent a barrier to immunotherapy completion.

**Conclusions** OIT and SLIT have some success as desensitization therapies for food allergy, yet patient-centered studies concerning psychological outcomes remain limited. Further research into psychological interventions aimed at reducing the burden experienced by patients and their families is needed. Ultimately, understanding the psychological impact of OIT and SLIT will contribute to optimizing these therapies for patients with food allergy.

## 24

### “There’s always that time and energy put into research”—a qualitative investigation of the underappreciated time costs of food allergy

#### Golding, Michael A.^1, 2^, Protudjer, Jennifer L.^1, 2^

##### ^1^Children’s Hospital Research Institute of Manitoba, Winnipeg, MB, ^2^University of Manitoba, Winnipeg, MB

*Allergy, Asthma & Clinical Immunology* 2026, **22(2)**: 24

**Background** Quantitative research suggests that households managing food allergy shoulder higher direct and indirect costs compared to those who do not manage food allergy. However, few studies have fully described what accounts for these excess costs or how they are perceived by those who incur them. To address this gap, the current study aimed to better understand how participants perceive the time costs of food allergy.

**Methods** Participants were recruited between December 2024 and March 2025 through online food allergy support groups and the research team’s social media. Adults responsible for managing a doctor-diagnosed, Immunoglobulin E-mediated food allergy—either their own or a dependent’s—were eligible. After consenting, participants completed a one-on-one interview focused on food allergy costs. Recordings from the interviews were transcribed and analyzed using thematic analysis.

**Results** The final sample included 18 female participants: 11 caregivers to a child with food allergy, 6 adults with food allergy, and 1 who reported managing both their own and a child’s food allergy. Two novel themes emerged regarding the time costs of managing food allergy. In the first theme, *Search costs: groceries, restaurants, and travel*, participants expressed frustration over the additional steps they had to take in order to identify safe food products and restaurants, both at home and while on vacation. In the second theme, *What’s in the cake?: planning for social events*, participants described how attending social events often demanded a considerable investment of time, and in some cases emotional effort, due to the need to determine whether safe food was available by default or through accommodation.

**Conclusions** Participants reported being burdened by time costs related to finding safe food options; however, these are not typically assessed when estimating the costs of food allergy. As such, current estimates may understate the true financial burden of food allergy.

## 25

### Lost in transition: understanding food allergy immunotherapy dropout in adolescents and young adults

#### Mah, Wesley^1^, Mak, Raymond^1^, Chan, Edmond^1^, Tong, Jennifer^1^, Protudjer, Jennifer^2^, Golding, Michael^2^, Dhaliwal, Ravinder^1^, Soller, Lianne^1^

##### ^1^University of British Columbia, Department of Pediatrics, Division of Allergy, Vancouver, BC, ^2^University of Manitoba, Department of Pediatrics and Child Health, Winnipeg, MB

*Allergy, Asthma & Clinical Immunology* 2026, **22(2)**: 25

**Background** The transition from adolescence to young adulthood is a critical period for maintaining continuity in allergy immunotherapy. BC Children’s Hospital has successfully supported many adolescents with food allergies through structured programs, like sublingual immunotherapy (SLIT) and oral immunotherapy (OIT), which have shown to reduce allergic reactivity and improve quality of life. However, a decline in adherence is commonly observed as patients transition to adult care. We sought to determine the dropout rate and reasons for dropout in a cohort of patients on SLIT or OIT during transition to Vancouver General Hospital.

**Methods** Patient chart notes from BC Children’s and Vancouver General Hospitals were qualitatively reviewed, and factors for treatment discontinuation were recorded and categorized to identify recurring trends in adherence during the transition to adult care. Adherence was defined as long-term participation, while discontinuation referred to treatment cessation.

**Results** Between Sept/2023 and June/2025, 30 patients were followed through transition, with 13 (aged 16–21 years) discontinuing treatment (43.3%). Patients were followed for 2 to 4 years, from the start of SLIT treatment to their first visit with the adult allergist, with a median age of 18. Without parental involvement in daily dosing, busy schedules and shifting priorities made it difficult for university students to prepare SLIT doses or source ingredients for OIT, accounting for 7 of 13 (53.8%) dropouts. Three of 13 (23.1%) who discontinued were lost to follow-up, reporting confusion about treatment duration, follow-up expectations, and responsibility for ongoing allergy management. Another 3 of 13 (23.1%) discontinued due to side effects like severe eczema, bradycardia, chest pain, and frequent colds.

**Conclusions** The transition to adult care is a critical period where decreased adherence in young adults can lead to dropouts. Empowering adolescents earlier in their treatment and producing more efficient dosing methods and better communication may help sustain adherence and preserve long-term outcomes.

## 26

### Efficacy of epinephrine nasal spray in children with anaphylaxis symptoms during oral food challenge

#### Ebisawa, Motohiro^3^, Takahashi, Kyohei^3^, North, Michelle^2^, Lowenthal, Richard^1^, Tanimoto, Sarina^1^

##### ^1^ARS Pharmaceuticals Operations, Inc., San Diego, CA, USA, ^2^ALK, Mississauga, ON, ^3^Clinical Research Center for Allergy and Rheumatology, NHO Sagamihara National Hospital, Sagamihara, Japan

*Allergy, Asthma & Clinical Immunology* 2026, **22(2)**: 26

**Background** Epinephrine nasal spray is indicated in the United States and other countries for the treatment of type I allergic reactions, including anaphylaxis. This open-label phase 3 study (jRCT2031230143) evaluated the efficacy and pharmacodynamics of epinephrine nasal spray in Japanese children with anaphylaxis symptoms induced by oral food challenge (OFC).

**Methods** Children with food allergy (6–17 years; N = 80) who participated in an OFC were screened. All participants were asked to use the nasal epinephrine placebo device before OFC so they were familiar with the device. The investigator administered epinephrine nasal spray 1 mg or 2 mg, according to the weight-based indication (1 mg for 15–30 kg and 2 mg for ≥ 30 kg) to children who experienced grade ≥ 2 gastrointestinal, respiratory, or cardiovascular symptoms per Japanese Anaphylaxis Guidelines.

**Results** Fifteen of the children undergoing OFC experienced 18 grade 2 symptoms and received epinephrine nasal spray, administered by the investigator. Six of the children weighed between 15 and 30 kg and received 1 mg, and 9 of the children weighed ≥ 30 kg and received 2 mg. The mean total grade of symptoms started decreasing within 5 min of nasal epinephrine administration and all patients (100.0%) responded with a clinically meaningful reduction of symptoms, with no patient requiring additional epinephrine for initial OFC-induced symptoms. One patient required additional epinephrine administration 2 h and 45 min after the initial dose. Pharmacodynamic effects were consistent with the pharmacological properties of epinephrine.

**Conclusions** Treatment with epinephrine nasal spray led to resolution of anaphylaxis symptoms induced by OFC, providing data supporting the efficacy of nasal epinephrine spray in treating anaphylaxis.

## 27

### Ease of use and successful administration of epinephrine nasal spray

#### Hernandez-Trujillo, Vivan^2^, Casale, Thomas B.^3^, Liu, Yun^5^, North, Michelle^1^, Lowenthal, Richard^4^, Tanimoto, Sarina^4^

##### ^1^ALK, Mississauga, ON, ^2^Nicklaus Children’s Hospital, Miami, FL, USA, ^3^Morsani College of Medicine, University of South Florida, Tampa, FL, USA, ^4^ARS Pharmaceuticals Operations, Inc., San Diego, CA, USA, ^5^Central Hospital of Xuhui District, Shanghai, China

*Allergy, Asthma & Clinical Immunology* 2026, **22(2)**: 27

**Background** In real-world anaphylaxis situations, epinephrine will likely need to be self-administered or administered by a caregiver. Epinephrine nasal spray 2 mg and 1 mg is indicated in the US and other countries for treatment of type I allergic reactions, including anaphylaxis. We present the results of ease of use and self-administration studies.

**Methods** A human factors study evaluated whether 16 untrained subjects (4 adult patients; 4 adult caregivers; 8 juveniles) with type 1 allergy could correctly administer an epinephrine nasal spray device in a simulated allergic emergency. In two additional randomized phase 1 crossover studies (one in US subjects with history of allergic rhinitis [N = 42] and one in healthy Chinese subjects [N = 42]), the pharmacokinetics and pharmacodynamics of self-administered epinephrine nasal spray 2 mg was compared with intramuscular epinephrine 0.3 mg administered by a healthcare provider (HCP).

**Results** In the human factors study, 100% of participants successfully loaded the carrying case with two devices, opened the case during a simulated allergy emergency, removed the devices, and administered epinephrine nasal spray twice. In the US study, 100% of subjects successfully self-administered the dose with no errors (mean dose time = 18 s). Mean peak plasma epinephrine concentrations (C_max_) were 421 pg/mL with self-administered nasal spray 2 mg and 322 pg/mL with HCP-administered intramuscular epinephrine 0.3 mg. Median time to C_max_ was 30 min with nasal spray and 45 min with intramuscular epinephrine. In the study of healthy Chinese participants, C_max_ was 755 pg/mL with self-administered nasal spray 2 mg and 617 pg/mL with HCP-administered intramuscular epinephrine 0.3 mg. Time to C_max_ was 30 min with both nasal and intramuscular epinephrine.

**Conclusions** Patients and caregivers are able to successfully administer epinephrine nasal spray. The pharmacokinetic profile after self-administered epinephrine nasal spray 2 mg was comparable to or better than HCP-administered intramuscular epinephrine 0.3 mg.

## 28

### Efficacy and safety of various routes of epinephrine administration for anaphylaxis: a systematic review and meta-analysis

#### Ologundudu, Leonardo, Chu, Alexandro W., Chu, Derek K.

##### McMaster University, Hamilton, ON

*Allergy, Asthma & Clinical Immunology* 2026, **22(2)**: 28

**Background** Epinephrine is the first-line therapy in treating anaphylaxis. Despite recent developments exploring alternative administration routes such as intranasal and sublingual, no studies directly compare the benefits and harms of all routes of epinephrine administration. Therefore, we will perform a systematic review and meta-analysis comparing the efficacy and safety of various routes of epinephrine administration for the management of anaphylaxis.

**Methods** We performed a preliminary systematic search of MEDLINE, EMBASE, and CENTRAL from inception to September 30, 2025, for randomized and non-randomized studies investigating various routes of epinephrine administration in patients experiencing anaphylaxis. Paired reviewers will independently screen records and extract data. Outcomes of interest will include relevant patient-important clinical outcomes and harms, and if data is sparse, relevant pharmacokinetic and pharmacodynamic outcomes from non-anaphylaxis populations. We will assess risk of bias independently and in duplicate for each study outcome. We will identify relevant subgroups a priori. We will use the GRADE approach to evaluate certainty of evidence.

**Results** Our preliminary search identified 5840 unique records, with additional studies to be identified through forward and backward citation analysis and examination of references from relevant systematic reviews. The relevant literature predominantly consists of non-randomized studies examining the management of anaphylaxis in emergency settings. There are currently at least 375 full-texts to examine in full-text screening.

**Conclusions** In this preliminary systematic search addressing various routes of epinephrine administration for anaphylaxis, there is a large body of literature to synthesize to inform future optimal management of anaphylaxis.

## 29

### Food allergy and stigma—engaging and collaborating with individuals and groups who experience stigma in research

#### Snarr, Phillip^1, 2^, Golding, Michael A.^1, 2^, Protudjer, Jennifer L.^1, 2, 3^

##### ^1^Department of Pediatrics and Child Health, University of Manitoba, Winnipeg, MB, ^2^Children’s Hospital Research Institute of Manitoba, Winnipeg, MB, ^3^Institute of Environmental Medicine, Karolinska Institutet, Stockholm, Sweden

*Allergy, Asthma & Clinical Immunology* 2026, **22(2)**: 29

**Background** Individuals and families living with food allergy, particularly those in lower socio-economic status (SES) groups and those experiencing food insecurity, may experience stigma. While the intersection between food allergy and food insecurity is increasingly being recognized as important, the stigma surrounding these topics can pose challenges in reaching research participants managing the dietary restrictions, inadequate food access, and poverty. In light of this, we sought to examine the literature to learn how to better engage with populations who experience stigma for the dual purposes of learning about their lived experiences and to better inform future research, and learning how to enhance the comfort of these individuals and groups to both collaborate and participate in food allergy research.

**Methods** We conducted a narrative review of the literature on conducting research on stigmatized populations and topics. Keywords used in our literature search were “food allergy”, “stigma”, “research”, and “recruitment”. From this review, we identified common themes that could be used to improve our collaboration with individuals with food allergy who experience a particularly high level of stigma.

**Results** From our literature review, we found three main themes. Theme 1 was recognizing and validating that the population is stigmatized. Theme 2 was creating partnerships with the stigmatized groups. Theme 3 centered on transparency and establishing trust in the current research project. We also identified additional questions to consider when collaborating on research with stigmatized populations. One additional question for us to consider is how can we create more opportunities throughout the research process to incorporate feedback from stigmatized individuals and groups?

**Conclusions** The sensitive nature of conducting research and collaborating on stigmatized topics, like food allergy and food insecurity, requires additional care and establishment of trust in order to engage with the populations that we seek to serve through our research.

## 30

### Autoclaving increases peanut tolerability in allergic subjects during oral food challenges

#### Toscano Rivero, Diana^1^, Cohen, Casey G.^1^, Ahmed, Eisha A.^1^, Al Ali, Adnan^2^, Alrafiaah, Abdulaziz S.^2^, Beaudette, Liane^2^, Lejtenyi, Duncan^2^, Chazbey, Hana^2^, McCusker, Christine^1, 2^, Jean-Claude, Bertrand J.^1^, Ben-Shoshan, Moshe^2, 1^, Mazer, Bruce D.^1, 2^

##### ^1^Research Institute of the McGill University Health Centre, Montreal, QC, ^2^Division of Allergy and Immunology, Department of Pediatrics, Montreal Children’s Hospital, Montreal, QC

*Allergy, Asthma & Clinical Immunology* 2026, **22(2)**: 30

**Background** Peanut oral immunotherapy (OIT) is a promising approach for treating peanut allergies but is often limited by frequent allergic reactions. Emerging data suggest that autoclaving peanuts under high temperature and pressure may significantly reduce IgE binding. We present group results and a representative case highlighting the impact of autoclaving on clinical reactivity in peanut-allergic individuals.

**Methods** Ten peanut-allergic individuals (median age 25 years; 3 females) were recruited to undergo double-blind oral food challenges with blanched or autoclaved (130 °C, 30 min) peanuts at the Montreal Children’s Hospital. Tolerated doses, skin prick test (SPT) results, and allergic reactions were recorded and compared. As an illustrative example, we highlight a 25-year-old male participant with high peanut sensitization (peanut sIgE: 1612.31 kU/L; sIgG4: 95.31 µg/ml; skin prick test: 24 mm). All participants provided written informed consent, and the study was approved by the institutional research ethics board.

**Results** All participants tolerated the maximum cumulative dose of 444 mg autoclaved peanut with only mild symptoms not requiring treatment. In contrast, the median tolerate dose for blanched peanuts was 9 mg (range: 1-44 mg), with all participants experiencing more severe reactions requiring epinephrine. SPT wheal diameters were significantly smaller for autoclaved peanut extract compared to standard extract (p < 0.05). In the highlighted case, the participant developed severe anaphylaxis (CoFAR 4) after having a cumulative tolerated dose of 144 mg blanched peanut, requiring epinephrine, antihistamines, and corticosteroids. In contrast, the same participant received a cumulative dose of 444 mg autoclaved peanut having only mild throat tingling (CoFAR 1) and no need for intervention.

**Conclusions** This study demonstrates that autoclaving peanuts may significantly reduce allergenicity, enabling peanut-allergic individuals to tolerate higher doses with milder reactions. The standout case exemplifies the difference in reactivity within a given patient, in line with the broader group findings. These results suggest autoclaving could improve the safety of peanut OIT, warranting further clinical trials.

*The illustrated participant gave their informed and explicit consent to publish their data in an online, open-access journal.*

## Immunology

## 31

### Clinically meaningful improvements with dupilumab in a range of symptoms and quality of life in adolescents and adults with eosinophilic esophagitis

#### Peterson, Kathryn^2^, Dellon, Evan S.^3^, Schoepfer, Alain^4^, Racca, Francesca^5^, AlKhouri, Rami^1^, Gonzalez, Carlos^6^, Zaghloul, Sherif^7^, Raphael, Bram P.^6^, Tilton, Sarette T.^8^, Thomas, Ryan B.^6^

##### ^1^Sanofi, North York, ON, ^2^Division of Gastroenterology, University of Utah, Salt Lake City, UT, USA, ^3^Center for Esophageal Diseases and Swallowing, University of North Carolina School of Medicine, Chapel Hill, NC, USA, ^4^Division of Gastroenterology and Hepatology, Department of Internal Medicine, Centre Hospitalier Universitaire Vaudois and University of Lausanne, Lausanne, Switzerland, ^5^Personalized Medicine, Asthma and Allergy Clinic, IRCCS Humanitas Research Hospital, Rozzano, Milan, Italy, ^6^Regeneron Pharmaceuticals Inc., Tarrytown, NY, USA, ^7^Sanofi, Morristown, NJ, USA, 8. Sanofi, Cambridge, MA, USA

*Allergy, Asthma & Clinical Immunology* 2026, **22(2)**: 31

**Background** Eosinophilic esophagitis (EoE) is a type 2 inflammatory disease in which interleukin (IL)-4 and IL-13 contribute to esophageal remodeling. Symptoms, including dysphagia, vomiting, chest pain, stomach pain, heartburn, and regurgitation, can reduce quality of life (QoL) in patients. In the phase 3 LIBERTY EoE TREET study (NCT03633617), dupilumab improved QoL, dysphagia, and non-dysphagia symptoms vs placebo. We report the proportion of patients with clinically meaningful improvements in symptoms of EoE and QoL with dupilumab vs placebo.

**Methods** This analysis used pooled data from Parts A and B of LIBERTY EoE TREET in patients aged ≥ 12 years receiving weekly dupilumab 300 mg or placebo. Symptoms were assessed using the Dysphagia Symptom Questionnaire (DSQ) and EoE Symptom Questionnaire (EoE-SQ). QoL was assessed using the EoE Impact Questionnaire (EoE-IQ). Proportions of patients achieving clinically meaningful thresholds, established using the Patient Global Impression of Severity (of dysphagia) as an anchor, were compared between dupilumab and placebo at Week 24; *P*-values are nominal.

**Results** 240 patients were included (placebo n = 118; dupilumab n = 122). At Week 24, DSQ scores were improved with dupilumab vs placebo (LS mean change [95% confidence interval]: –22.62 [–25.63, –19.62] vs –12.05 [–15.18, –8.92], respectively; *P* < 0.0001). A greater proportion of dupilumab-treated patients achieved clinically meaningful improvement (≥ 13-point reduction) in DSQ score vs placebo (72.1% vs 44.1%; *P* < 0.0001). Significantly more dupilumab-treated patients also had clinically meaningful improvements in EoE-SQ Frequency score (≥ 3.7-point reduction; 50.0% vs 30.5%; *P* = 0.002), EoE-IQ score (≥ 0.6-point reduction; 64.8% vs 41.5%; *P* = 0.0001), and a more stringent ≥ 24-point reduction in DSQ (48.4% vs 18.6%; *P* < 0.0001). A numerically greater proportion had clinically meaningful improvement in EoE-SQ Severity score (≥ 5.3-point reduction) with dupilumab vs placebo (43.4% vs 32.2%; *P* = 0.0746).

**Conclusions** Dupilumab resulted in a greater proportion of patients with EoE achieving clinically meaningful improvement in dysphagia, non-dysphagia symptoms, and QoL vs placebo at Week 24.

## 32

### Effect of dupilumab on weight in children with active EoE enrolled in the phase 3 EoE KIDS study

#### Chehade, Mirna^1^, Pesek, Robert D.^2^, Menard-Katcher, Calies^3^, Gutmark-Little, Iris^4^, AlKhouri, Rami^5^, Liu, Ruiqi^6^, Robinson, Lacey^7^, Maloney, Jennifer^6^, Louisias, Margee^7^, Radin, Allen^6^

##### ^1^Mount Sinai Center for Eosinophilic Disorders, Icahn School of Medicine at Mount Sinai, New York, NY, USA, ^2^Department of Pediatrics, Division of Allergy/Immunology, University of Arkansas for Medical Sciences and Arkansas Children’s Hospital, Little Rock, AR, USA, ^3^Digestive Health Institute, Children’s Hospital Colorado, and Gastrointestinal Eosinophilic Diseases Program, University of Colorado School of Medicine, Aurora, CO, USA, ^4^Division of Pediatric Endocrinology, Cincinnati Children’s Hospital Medical Center, Cincinnati, OH, USA, ^5^Sanofi, Toronto, ON, ^6^Regeneron Pharmaceuticals Inc., Tarrytown, NY, USA, ^7^Sanofi, Cambridge, MA, USA

*Allergy, Asthma & Clinical Immunology* 2026, **22(2)**: 32

**Background** Eosinophilic esophagitis (EoE) is a chronic, progressive, type 2 inflammatory disease of the esophagus that has a significant impact on quality of life. In children, complications include malnutrition and failure to thrive. Dupilumab is a fully human monoclonal antibody that inhibits interleukin (IL)-4 and IL-13 signaling and is approved for the treatment of EoE in patients aged ≥ 1 year, weighing ≥ 15 kg. This analysis assessed the effect of weight-tiered, higher-exposure dupilumab vs placebo on weight outcomes in patients aged 1–11 years with active EoE enrolled in the phase 3 EoE KIDS study (NCT04394351).

**Methods** EoE KIDS Part A was a 16-week, randomized, placebo-controlled, double-blind treatment period where patients received either weight-tiered dupilumab or placebo. Patients who completed Part A were eligible to enter Part B, a 36-week extended treatment period during which all patients received dupilumab. Full details of the EoE KIDS study design have been reported previously. Change from baseline in body weight-for-age percentile, body mass index (BMI)-for-age z-score for patients aged ≥ 2 years, weight-for-age z-score, and weight-for-length z-score were assessed at Weeks 16 and 52.

**Results** At Week 16, mean change (standard deviation) from baseline in body weight-for-age percentile was + 3.09 (9.25) vs + 0.29 (6.21) for dupilumab vs placebo; change in BMI-for-age z-score was + 0.10 (0.50) vs –0.14 (0.32), change in weight-for-age z-score was + 0.12 (0.30) vs –0.01 (0.22), and change in weight-for-length z-score was + 0.18 (0.72) vs –0.05 (0.26). At Week 52, improvements in body weight-for-age percentile and BMI-for-age and weight-for-age z-scores were maintained or increased with continued dupilumab; improvements were also observed in placebo patients who switched to dupilumab.

**Conclusions** Dupilumab was associated with a greater increase in body weight-for-age percentile at Weeks 16 and 52 vs placebo. Dupilumab was also associated with sustained trends for increased BMI-for-age and weight-for-age z-scores vs placebo.

## 33

### Clinical characterization of adult primary and secondary immunodeficiency disorders: a single-center experience from the MUHC Immune Deficiency Treatment Centre

#### Benharira, Noha^1^, AlYaqout, Fatemah^2^, Polito, Vanessa^2^, Tsoukas, Christos^2^

##### ^1^Faculty of Medicine and Health Sciences, McGill University, Montréal, QC, ^2^Department of Allergy and Clinical Immunology, McGill University Health Center, Montréal, QC

*Allergy, Asthma & Clinical Immunology* 2026, **22(2)**: 33

**Background** Primary immunodeficiencies (PIDs) are rare genetic disorders that predispose patients to recurrent infections, malignancy, and immune dysregulation. While often treatable, diagnosis is complex, and delays can lead to significant morbidity and mortality. In 2012, our group published the largest cohort of adult with suspected PIDs, highlighting their wide clinical spectrum and the urgent need for increased physician awareness.

Since then, secondary immunodeficiencies (SIDs), acquired due to malignancies, immunosuppressive therapies, chronic infection, or malnutrition, are being recognized with increasing frequency. Distinguishing between PIDs and SIDs is critical for accurate diagnosis and effective management.

This study expands on our earlier findings by reviewing 20 years of adult referrals for suspected immune deficiency to our center, characterizing primary and secondary immunodeficiencies, assessing diagnostic accuracy, and examining trends in their distribution and associated clinical features.

**Methods** We are conducting a retrospective chart review of all adults referred to the MUHC IDTC over a 20-year period. Eligible patients are ≥ 18 years at initial evaluation, with available referral details, clinical presentation, and final diagnosis. Cases will be classified as PID or SID using the 2024 International Union of Immunological Societies (IUIS) phenotypic classification. Extracted data include demographics, referral source, clinical features, laboratory results, and final diagnosis. Descriptive statistics will summarize cohort characteristics. Group comparisons will use the Mann–Whitney U test for non-normally distributed variables and Student’s t-test for normally distributed variables.

**Results** Though data collection is still ongoing, preliminary review indicates higher proportions of SIDs and of specific PIDs with genetic diagnoses.

**Conclusions** Higher proportions of SIDs and specific PIDs directly signifies innovation in genetics and immune modulating therapies. Our study will provide valuable insight into the spectrum of adult PIDs while reflecting on recent diagnostic advancements in the field of clinical immunology. We aim to enhance awareness and improve referral practices for suspected immune deficiencies.

## 34

### ENT perspectives and experience on diagnosing, treating, and managing patients with primary immunodeficiency disorders (PIDD): a national Canada survey

#### Damask, Cecelia^1^, Hsieh, Elena^2^, Calderón-Gómez, Elisabeth^3^, Burkovskiy, Ian^4^

##### ^1^Orlando ENT & Allergy, Orlando, FL, USA, ^2^Children’s Hospital Colorado, Aurora, CO, USA, ^3^Grifols, Barcelona, Spain, ^4^Grifols, Mississauga, ON

*Allergy, Asthma & Clinical Immunology* 2026, **22(2)**: 34

**Background** Primary immunodeficiencies disorders (PIDD) result from inherited defects in the immune system. Patients with PIDD often experience recurrent or chronic ear, nose, and throat infections. Early diagnosis is crucial to prevent complications, thereby improving morbidity and quality of life. The aim was to assess ear, nose, and throat specialists’ (ENT) awareness and experience in diagnosing and treating PIDDs.

**Methods** A blinded survey conducted in Canada, consisting of 34 questions, evaluated the experience of ENT, their collaboration with other specialists, the challenges they face, and their interest in educational opportunities related to PIDD.

**Results** 21 Canada-based ENT participated in the survey. 81% of them encountered patients with PIDD with frequent, severe, and/or unusual infections. However, only 20% adhered to specific guidelines, and just 5% were aware of recent studies. Additionally, 38% had never engaged with PIDD educational opportunities. This highlights a clear gap between the number of diagnosed patients with PIDD and guidelines awareness. 67% preferred IVIg therapy over SCIg, and 38% used prophylactic antibiotics for patients with PIDD. Ig efficacy was assessed by infection frequency (67%), symptom improvement (50%), severity of infections (44%), and Ig levels (11%). Most respondents (78%) tested IgG levels, but only 22% knew the target trough IgG levels, and 39% were aware of dosing schedules. ENT referred patients with PIDD to clinical immunologists rather than hematologists. The challenges faced by ENT specialists included access to immunodiagnostic, delays in seeing specialists and knowledge gaps. Notably, respondents expressed interest in case-based learning formats for PIDD education.

**Conclusions** Although this survey revealed that ENT frequently encountered patients with PIDD, a gap in guidelines awareness was identified. Early diagnosis and intervention by ENT and PIDD-targeted education may improve outcomes in these patients.

## 35

### Cytokine profiling of PBMCs to guide targeted therapy in patients with immune dysregulation

#### Marois, Louis^1, 2, 5^, Fortin, Christopher^2, 5^, Breton, Yann^2, 5^, Chetaille, Anne-Laure^3, 5^, Yeganeh, Mehdi^3^, Tessier, Philippe^4, 5^, Pelletier, Martin^2, 4, 5^

##### ^1^CHU de Québec—Université Laval, Québec, QC, ^2^Axe Maladies infectieuses et immunitaires, CRCHU de Québec-Université Laval, Québec, QC, ^3^Axe Reproduction, santé de la mère et de l’enfant, CRCHU de Québec-Université Laval, Québec, QC, ^4^Département de microbiologie-Infectiologie et immunologie, Faculté de Médecine, Université Laval, Québec, Canada, Québec, QC, ^5^Centre de recherche ARThrite, Université Laval, Québec, QC

*Allergy, Asthma & Clinical Immunology* 2026, **22(2)**: 35

**Background** Inborn errors of immunity (IEIs) are characterized by pathophysiological abnormalities of the immune system, leading to a wide spectrum of clinical manifestations. Due to the heterogeneity of presentations and the lack of specific diagnostic tools, therapeutic decisions are often made by trial and error, delaying appropriate treatment. There is an unmet need for functional diagnostic tools to guide biologic therapy in patients with immune dysregulation.

**Methods** We developed a functional diagnostic assay based on the cytokine profiling of peripheral blood mononuclear cells (PBMCs). PBMCs are isolated from blood samples and stimulated with various pro-inflammatory cytokines, lipopolysaccharide (LPS), and other immune activators. Cytokine secretion is quantified using Luminex multiplex technology. Patient cytokine profiles are then compared to those of healthy controls to identify abnormal immune signaling pathways.

**Results** This approach led to targeted therapies in two patients followed at the Immuno-Rheumatology-Genetics Clinic of CHU de Québec – Université Laval.

Patient 1: A 63-year-old man presented with unexplained recurrent fevers exceeding 40 °C. PBMC stimulation revealed excessive TNF secretion, undetectable in plasma. Anti-TNF therapy successfully prevented further febrile episodes.

Patient 2: A 28-year-old woman experienced arthralgia, myalgia with weakness, and cutaneous lesions. Her PBMC cytokine profile showed elevated IL-6, IL-12, and IL-21 levels—cytokines that signal via the JAK-STAT pathway. Despite normal plasma levels, this guided the initiation of JAK inhibitor therapy, resulting in clinical improvement.

**Conclusions** PBMC cytokine profiling provides insight into cellular immune dysregulation and can complement clinical and genetic data to guide targeted immunotherapy. As it better reflects tissue-level inflammation than plasma analysis alone, this assay represents a promising diagnostic tool for precision medicine in patients with immune dysregulation.

*Both patients gave their informed and explicit consent to have their data published in an online, open-access journal.*

## 36

### Newborn screening algorithms for severe combined immunodeficiency in Canada: a descriptive survey project

#### Martinez, Benjamin^1^, Antonishyn, Nick^2^, Berthier, Marie-Thérèse^3^, Brager, Rae^4^, Derfalvi, Beata^5^, Ellis, Anne K.^6^, Geerlinks, Ashley V.^7^, Giguère, Yves^3^, Hildebrand, Kyla J.^8^, Lacaria, Melanie^9^, Langlois, Alexandra^10^, Liu, Zaiping^11^, Marois, Louis^12^, Martin, Georgina^13^, Muranyi, Andrew^14^, Rubin, Tamar^15^, Sinclair, Graham^16^, Suresh, Sneha^17^, Touzot, Fabien^18^, Biggs, Catherine M.^19^, Wright, Nicola A. M.^20^, Pham-Huy, Anne^21^, Kim, Vy H. D.^22^

##### ^1^Department of Paediatrics, The Hospital for Sick Children, Toronto, ON, ^2^Roy Romanow Provincial Laboratory, Regina, SK, ^3^Quebec NBS program, Clinical Department of Laboratory Medicine, CHU de Québec-Université Laval, Québec, QC, ^4^Division of Immunology, Allergy, and Dermatology, Department of Pediatrics, McMaster University, Hamilton, ON, ^5^Department of Paediatrics, IWK Health, Halifax, NS, ^6^Division of Allergy & Immunology, Department of Medicine, Queen’s University, Kingston, ON, ^7^Department of Pediatrics, Western University, London, ON, ^8^Division of Immunology, Department of Pediatrics, Faculty of Medicine, University of British Columbia, Vancouver, BC, ^9^Newborn Screening Ontario, Children’s Hospital of Eastern Ontario, Ottawa, ON, ^10^Department of Paediatrics, Université de Sherbrooke CIUSSS de l’Estrie- CHUS, Sherbrooke, QC, ^11^Department of Pathology and Laboratory Medicine, IWK Health, Halifax, NS, ^12^Department of Specialized Medicine, Division of Immunology-Allergy, CHU de Québec-Université Laval, Québec, QC, ^13^Department of Pediatrics, Jim Pattinson Children’s Hospital, Saskatoon, SK, ^14^Genetics and Genomics, Alberta Precision Laboratories, Edmonton, AB, ^15^Department of Pediatric Clinical Immunology and Allergy, Children’s Hospital Winnipeg, Winnipeg, MB, ^16^Department of Pathology and Laboratory Medicine, BC Children’s Hospital/UBC, Vancouver, BC, ^17^Department of Pediatrics, University of Alberta, Edmonton, AB, ^18^Department of Paediatrics, CHU Sainte Justine, Université de Montréal, Montréal, QC, ^19^Department Pediatrics, BC Children’s Hospital Research Institute, University of British Columbia, Vancouver, BC, ^20^Division of Hematology/Immunology, Alberta Children’s Hospital, University of Calgary, Calgary, AB, ^21^Division of Infectious Diseases, Children’s Hospital of Eastern Ontario, University of Ottawa, Ottawa, ON, ^22^Division of Immunology and Allergy, Department of Pediatrics, The Hospital for Sick Children, Temerty School of Medicine, University of Toronto, Toronto, ON

*Allergy, Asthma & Clinical Immunology* 2026, **22(2)**: 36

**Background** Newborn screening (NBS) for severe combined immunodeficiency (SCID) was first implemented in Canada in 2013, with most provinces and territories now including SCID in their NBS programs. This study aimed to describe SCID screening approaches across Canada.

**Methods** A REDCap survey was distributed to representatives from Canadian SCID NBS programs, retrieval centres, and laboratories. The survey addressed processes following a positive screen, including retrieval, counselling, screening targets, definitions, and barriers. Categorical responses were summarized quantitatively and narrative responses were analyzed using inductive coding.

**Results** The response rate was 91% of 22 distributed surveys, with representation from all screening programs. All programs share comparable primary screening targets, encompassing the full spectrum of SCID phenotypes, though secondary and incidental targets differ. T-cell receptor excision circle (TREC) quantification universally serves as the initial screening modality, with select provinces incorporating supplementary assays including kappa-deleting recombination excision circles (33%), purine metabolite profiling (17%), and targeted molecular diagnostics (33%). Repeat TREC is mainly indicated for preterm (83%) and low-birth-weight neonates (67%). First retrieval investigations typically include a complete blood count with differential (100%), quantitative lymphocyte subset assay (100%), and naïve/memory T-cell immunophenotyping (71%), with further testing based on institutional access and targets. Core counselling themes are consistent, including infection control, live vaccine avoidance, and breastfeeding recommendations. Common systemic barriers to retrieval of confirmatory samples and in-person assessment include geographic remoteness, inter-facility coordination, and limited laboratory availability during off-hours.

**Conclusions** Programs across Canada share common primary screening targets, but differ in algorithms and resources, which may contribute to variation in diagnosis and early management. These differences highlight the need to analyze screening outcome data to evaluate current processes and collaboratively explore whether a more unified approach would be beneficial. The identification of shared barriers, particularly those affecting remote populations, presents an opportunity for further solution-focused research.

## 37

### Co-designing the Canadian inborn errors of immunity registry: embedding patient and caregiver partnership in development and knowledge translation

#### Salimi, Payam^1^, Suresh, Sneha^2^, Brager, Rae^3^, Hunt, Brianna^4^, Murguia-Favela, Luis^5^, Kalashnikova, Tatiana^5^, Morley, Madeleine^6^, Ayoub-Goulstone, Whitney^6^, Grunebaum, Eyal^7^, Ritchie, Bruce^8^, Cowan, Juthaporn^9^, Biggs, Catherine^10^, Chapdeleine, Hugo^11^, Geerlinks, Ashley^12^, Wright, Nicola^5^, Derfalvi, Beata^13^, Rubin, Tamar^14^

##### ^1^Department of Internal Medicine, University of Manitoba, Winnipeg, MB, ^2^Department of Pediatrics, University of Alberta, Edmonton, AB, ^3^Division of Immunology, Allergy, and Dermatology, Department of Pediatrics, McMaster University, Hamilton, ON, ^4^Children’s Hospital Research Institute of Manitoba, Winnipeg, MB, ^5^Dept of Pediatrics, Cumming School of Medicine, University of Calgary, Calgary, AB, ^6^Immunity Canada, Victoria, BC, ^7^The Division of Immunology and Allergy, The Hospital for Sick Children, Toronto, ON, ^8^Department of Medicine, Division of Hematology, University of Alberta, Edmonton, AB, ^9^Dependent of Medicine, University of Ottawa at the Ottawa Hospital and the Ottawa Hospital Research Institute, Ottawa, ON, ^10^BC Children’s Hospital, University of British Columbia, Vancouver, BC, ^11^Division of Clinical Immunology, Montreal Clinical Research Institute, Montreal, QC, ^12^Children’s Hospital of Western Ontario, Western University, London, ON, ^13^Division of Immunology, Department of Paediatrics, Dalhousie University, IWK Health Centre, Halifax, ON, ^14^Section of Clinical Immunology and Allergy, Department of Pediatrics & Child Health, University of Manitoba, Winnipeg, MB.

*Allergy, Asthma & Clinical Immunology* 2026, **22(2)**: 37

**Background** Inborn errors of immunity (IEI) are rare, often life-threatening disorders that compromise immune function and profoundly impact individuals and families across Canada. Despite an estimated 30,000 Canadians living with IEI, national data to guide clinical care, research, and health policy remain limited. The Canadian Inborn Errors of Immunity National Registry (CIEINR) was established to address these critical knowledge gaps and improve understanding of the burden and diversity of IEI nationally. Meaningful patient and caregiver partnership is essential to ensuring the CIEINR reflects the needs, priorities, and lived experiences of those it serves. To achieve this, we planned an integrated patient engagement initiative to guide CIEINR development and co-create a bilingual, public-facing website to support knowledge sharing.

**Methods** We are implementing a structured, multi-phase approach to patient and caregiver engagement throughout CIEINR development. With guidance from a patient engagement specialist, up to five patient and caregiver partners will be recruited, reflecting the diversity of the Canadian IEI community. These partners will receive orientation and coaching to contribute meaningfully to CIEINR governance, protocol development, knowledge translation activities, and the co-development of a patient-accessible website. Additional stakeholder focus groups will inform website content and ensure broad knowledge user perspectives are integrated.

**Results** Anticipated outcomes include the co-creation of a responsive, patient-informed CIEINR platform and strengthened partnerships between researchers, clinicians, patients, and families.

**Conclusions** Embedding patient and caregiver voices throughout CIEINR development and knowledge translation is essential to building a trusted, relevant, and sustainable platform. Through meaningful partnership, the CIEINR aims to generate high-quality data, support advocacy efforts, and advance health equity for individuals and families affected by IEI across Canada.

## CASE REPORTS

## 38

### DRESS secondary to Galcanezumab – a severe cutaneous adverse reaction to a migraine anti-CGRP monoclonal antibody therapy

#### Robinson, Madeline^1^, McKibbin, Lundy^1,2,3^

##### ^1^Department of Internal Medicine, University of Manitoba, Winnipeg, MB, ^2^Section of Adult Clinical Immunology and Allergy, Department of Internal Medicine, University of Manitoba, Winnipeg, MB, ^3^Section of Pediatric Clinical Immunology and Allergy, Department of Pediatrics and Child Health, University of Manitoba, Winnipeg, MB

*Allergy, Asthma & Clinical Immunology* 2026, **22(2)**: 38

**Background** Galcanezumab is a anti-calcitonin-gene-related-peptide (CGRP) monoclonal-antibody therapy used to treat migraines. Drug-reaction-with-eosinophilia-and-systemic-symptoms (DRESS) is a severe cutaneous adverse drug reaction that has never been reported from this class of drug.

**Case Presentation:** We report a case of DRESS secondary to Galcanezumab in a healthy 71-year-old male with no other medication use. The patient received 3 monthly Galcanezumab injections prior to presenting with pleuritic chest pain and a 2-day history of maculopapular rash with facial edema and cervical lymphadenopathy. He was febrile and hypoxic, with mucocutaneous ulcers, acute kidney injury, mixed liver enzyme elevation, and moderate peripheral eosinophilia. Imaging showed splenomegaly, abdominopelvic lymphadenopathy, and multifocal ground-glass opacities. Initial RegiSCAR of 5 suggested probable DRESS. Steroids were started, but work-up for alternative etiologies was recommended due to atypical features, timeline, and lack of supporting literature. Hematology and Respirology were consulted. Blood cultures, HIV, CMV, EBV, HHV-6, and viral hepatitis serologies were negative. JAK-2 & BCR-ABL were negative. Peripheral smear, flow cytometry, and bone marrow biopsy were unremarkable. ANA, anti-dsDNA, ANCAs, MPOand PR3, SPEP and FLCR were negative. Skin punch biopsy showed non-specific inflammatory changes. Bronchoalveolar lavage and endobronchial lymph node biopsy were normal.

The patient improved with steroids – his rash, hypoxia, eosinophilia, kidney and liver injuries resolved. RegiSCAR at discharge was 7; DRESS was thus the most likely diagnosis following extensive negative work-up for alternative causes, with Galcanezumab the only possible culprit. The patient has done well, with a brief episode of eosinophilia and kidney injury, prompting even slower prednisone taper over manymonths.

**Conclusions** This is the first reported case of a severe adverse cutaneous reaction to Galcanezumab specifically and anti-CGRP monoclonal antibody therapies in general.

*The patient gave their informed and explicit consent to publish their data in an online, open-access journal.*

## 39

### Donor acquired peanut allergy following a double lung transplant with negative sIgE and skin testing

#### Almanaseer, Ala^1^, Kanani, Amin^2^

##### ^1^Faculty of Medicine, University of British Columbia, Vancouver, BC, Canada., Vancouver, BC, ^2^Department of Medicine, Division of Allergy and Immunology, University of British Columbia, Vancouver, Canada., Vancouver, BC

*Allergy, Asthma & Clinical Immunology* 2026, **22(2)**: 39

**Background** Donor-acquired allergy (DAA) refers to allergic reactions that can be transferred to organ transplant recipients from donors with Immunoglobulin E (IgE)-mediated allergy. Despite the rare occurrence of DAA, previous reports have commonly involved pediatric recipients of liver and hematopoietic stem cell transplants. While the transmission of food allergies has previously been reported following lung transplant, DAA without concurrent positive specific IgE (sIgE) or skin prick testing (SPT) has not been previously reported.

**Case Presentation:** We report the case of a donor-acquired peanut allergy in a 62-year-old female following a bilateral lung transplant for sporadic lymphangioleiomyomatosis. Her immunosuppression consisted of tacrolimus, low-dose prednisone, and mycophenolate mofetil, which was later changed to sirolimus. She had no prior atopic history. However, the donor had an anaphylactic peanut allergy that prompted investigation in the recipient. She was assessed in the allergy clinic two months post-transplant, where she had negative peanut SPT and sIgE. On the first ingestion of peanuts following transplant, she reported dyspnea, audible inspiratory sounds, and self-administered intramuscular epinephrine. She was reassessed several times following this reaction and repeat SPT one-year post-transplant remained negative to peanut extract, peanut butter, and crushed peanuts. Repeat sIgE also remained negative. Given her reported reaction, she underwent an in-office graded oral challenge to fresh peanuts. Ten minutes after the ingestion of three fresh peanuts, she developed chest and throat tightness, as well as bilateral wheezing. Examination confirmed isolated expiratory and inspiratory wheezing without hemodynamic compromise or other systemic symptoms. She responded to Symbicort with complete resolution of symptoms within 40 min, and was instructed to avoid peanuts.

**Conclusions** Donor-acquired peanut allergy can occur following lung transplant despite negative SPT and sIgE testing, demonstrating possible testing limitations in the context of immunosuppression. This case emphasizes the importance of close allergy assessment post-transplant from an organ donor with known IgE-mediated allergies.

*The patient gave their informed and explicit consent to publish their data in an online, open-access journal.*

## 40

### Loss of tolerance after missed doses during food protein induced enterocolitis syndrome reintroduction therapy

#### Pawer, Samantha^1^, Ye, Linlei^2^, Jeimy, Samira^3, 4^, Erdle, Stephanie^5^, Cook, Victoria^5, 6^

##### ^1^Department of Pediatrics, University of Calgary, Calgary, AB, ^2^Division of Immunology and Allergy, The Hospital for Sick Children, Toronto, ON, ^3^Division of Clinical Immunology and Allergy, Department of Medicine, Western University, London, ON, ^4^Lawson Health Research Institute, London, ON, ^5^Division of Allergy, Department of Pediatrics, University of British Columbia, Vancouver, BC, ^6^Community Immunology & Allergy Clinic, Victoria, BC

*Allergy, Asthma & Clinical Immunology* 2026, **22(2)**: 40

**Background** Food reintroduction with structured dose advancement, or ladder therapy, is a proposed management strategy for food protein-induced enterocolitis syndrome (FPIES). Benefits include reduced duration of food avoidance and the associated risk of IgE-mediated allergy, and decreased need for hospital-based oral food challenges (OFCs). There are currently no studies evaluating whether this approach could impact desensitization or development of tolerance in patients with FPIES.

**Case Presentation:** Case 1: A 6-month-old female experienced recurrent vomiting, pallor and lethargy 4 h after eating ~ 3 tablespoons of avocado. At 15 months, she began daily avocado administration with 1/16th teaspoon, advancing to 1.5 teaspoons within 6 weeks. A one-month lapse in ingestion led to a reaction when restarting at 1 teaspoon, marked by 2 episodes of forceful emesis and pallor 4 h after ingestion. Avocado was successfully reintroduced at 1/16th teaspoon 2 weeks later, and serving sized amounts were tolerated within 7 months.

Case 2: An 8-month-old male experienced vomiting, diarrhea, pallor and lethargy 4 h after ingestion of ~ 1.5 g cooked chicken. At 10 months of age, reintroduction began with 0.05 g of chicken, advancing to 8 g daily over 8 weeks. After missing 3 consecutive weeks of dosing, restarting at 6 g resulted in a single episode of forceful emesis with onset 3 h after ingestion. Chicken was reintroduced at 1 g, with advancement to a serving size within 6 months.

**Conclusions** These cases suggest that interruptions in daily trigger food ingestion during ladder therapy for FPIES could result in recurrence of symptoms at previously tolerated amounts. They highlight the need for prospective studies to determine whether there is a role for reintroduction and ladder therapies beyond prevention of IgE-mediated food allergy and reduction in OFC requirements.

## The guardians of both patients gave their informed and explicit consent to publish their data in an online, open-access journal.

## 41

### Injectable corticosteroid-induced refractory anaphylactic shock

#### Wachtel, Amir^1^, Lee, Erika^3, 4^, Ip Fung Chun, Peter^2^

##### ^1^Department of Pediatrics, Queen’s University, Kingston, ON, ^2^Division of Allergy and Immunology, Department of Medicine, Queen’s University, Kingston, ON, ^3^Drug Allergy Clinic, Sunnybrook Health Sciences Centre, Toronto, ON, ^4^Division of Allergy & Immunology, Department of Medicine, University of Toronto, Toronto, ON

*Allergy, Asthma & Clinical Immunology* 2026, **22(2)**: 41

**Background** Corticosteroid-induced immediate drug hypersensitivity reaction (DHR) is rare with an estimated prevalence of 0.1–0.3% [1,2]. While immediate DHRs to corticosteroids have been described, their use resulting in an intensive care unit (ICU) admission for anaphylaxis is exceedingly rare. Amongst the limited number of reported cases available, none required an epinephrine infusion for refractory anaphylactic shock. Here we report such a case of severe anaphylaxis to intraarticular triamcinolone.

**Case Presentation:** A 55-year-old male underwent bilateral knee injections with 1% lidocaine and triamcinolone for osteoarthritis. The patient quickly developed light-headedness, flushing, warmth, lost consciousness and emergency medical services were called. Reports document diffuse erythema and hypotension (systolic 60 s, MAP 40 s). He remained hypotensive despite administration of IV fluids and two doses of 0.5 mg IM epinephrine. Subsequently an epinephrine infusion was initiated, for refractory anaphylactic shock. He was admitted to the ICU for a short period of monitoring and was seen in allergy clinic 2 years later for assessment. Intradermal skin testing (IDT) was done in the usual fashion with 1:1000 dilution of lidocaine 2% with and without preservative, bupivacaine 0.5%, and ropivacaine 5 mg/mL. A dilution of 1:10,000 for Triamcinolone 40 mg/1 ml was used. Triamcinolone IDT was positive with a wheal and flare reaction. An IDT to the same solution of Triamcinolone was done on a control subject which did not result in a wheal and flare reaction.

**Conclusions** This patient had a likely reaction to an intraarticular injection of Triamcinolone. Further testing will be arranged to rule out an excipient such as polyethylene glycol, polysorbate, or carboxymethylcellulose as the culprit agent. Here we present the initial allergy workup for a severe case of immediate hypersensitivity reaction to an injectable triamcinolone product.

*The patient gave their informed and explicit consent to publish their data in an online, open-access journal.*

## 42

### Effects of omalizumab in patients with mast cell disorders: the Halifax experience

#### Al-Qadi, Mohammad, Lacuesta, Gina

##### Dalhousie University, Halifax, NS

*Allergy, Asthma & Clinical Immunology* 2026, **22(2)**: 42

**Background** Mast cell disorders comprise a heterogeneous group of conditions, including mastocytosis and mast cell activation syndrome (MCAS). These disorders result from inappropriate mast cell activation and mediator release, leading to a broad range of manifestations that may affect the cardiovascular, respiratory, gastrointestinal and cutaneous systems. Pharmacological treatment typically includes H1 and H2 receptor antagonists, mast cell stabilizers, and leukotriene receptor antagonists. Given the refractory nature of some cases, omalizumab, a monoclonal anti-IgE antibody, has emerged as a promising therapeutic option.

**Case Presentation:** We describe the cases of six patients, four with MCAS and two with systemic mastocytosis. The most common symptoms included anaphylactoid reactions (5/6), flushing (5/6), urticaria (5/6), gastrointestinal symptoms (5/6) and angioedema (4/6). Three patients had a concurrent diagnosis of postural orthostatic tachycardia syndrome (POTS), and three patients had an asthma diagnosis. Omalizumab was started in all patients due to inadequate control on traditional therapies. Average age of initiation was 45 years of age. Five of six patients had noticeable benefit in symptoms on omalizumab, with one patient only experiencing slight improvement in symptoms.

**Conclusions** Omalizumab demonstrated clinical benefit in our patients with mast cell disorders, both MCAS and systemic mastocytosis, that were refractory to traditional therapeutic modalities. Further studies are needed to characterize its efficacy and identify patient characteristics associated with treatment response.

## 43

### Sézary syndrome treated with interferon-alpha: a diagnostic challenge in worsening erythroderma

#### Alnajjar, Nisreen^2^, Genest, Genevieve^1^

##### ^1^Mcgill University, Montréal, QC, 2. King AbdulAziz University, Jeddah, Saudi Arabia

*Allergy, Asthma & Clinical Immunology* 2026, **22(2)**: 43

**Background** Sézary syndrome (SS) is a leukemic variant of cutaneous T-cell lymphoma, characterized by erythroderma, peripheral lymphadenopathy, pruritus, and atypical lymphocytes in the blood. SS is associated with a shift toward a Th2-type immune response, resulting in impaired cell-mediated cytotoxicity and decreased natural killer (NK) cell activity. Interferon alpha (IFN α), has been identified as an effective therapy for SS. However, it has the potential to exacerbate chronic inflammatory disease and trigger autoimmune-like disorders.

**Case Presentation:** We report the case of 66-year-old female with SS treated with IFN-α who developed worsening pruritus and erythema after each infusion. This patient has a longstanding cutaneous symptoms manifesting as intense pruritus and erythroderma. She had been treated with multiple biologics until the diagnosis of SS was made upon skin biopsy. She was started on isotretinoin two months before presentation followed by extracorporeal photopheresis (ECP) and IFN-α infusion. After each IFN-α infusion, she would notice scalp pruritus and worsening erythema. She presented to the emergency room 24 h after her 4th dose of IFN-α with pancorporal erythroderma and worsening pruritus. A Skin biopsy showed lichenoid lymphoid infiltrate, comprised mainly of small CD3 + T-cell with increased CD4/CD8 ratio, a positive clonal T-cell receptor rearrangement with no large cell transformation, consistent with her previous diagnosis of SS. Given the temporal relationship between IFN-α administration and symptoms flares, interferon-induced immune exacerbation of SS was suspected. IFN-α was discontinued, leading to symptoms improvement. She was transitioned to Mogamulizumab® with gradual clinical improvement.

**Conclusions** This case highlight the potential for (IFN-α) to exacerbate chronic inflammatory disease or trigger autoimmune-like response. Mechanism may include increased activity of cytotoxic T cells and (NK) cells, increased antibody-dependent cell-mediated cytotoxicity, and suppression of regulatory (T-suppressor) function [6]. Clinicians should consider IFN-α–induced immune flares in patients experiencing symptoms worsening during (IFN-α) treatment.

*The patient gave their informed and explicit consent to publish their data in an online, open-access journal.*

## 44

### Idiopathic hypereosinophilic syndrome manifesting as atopy, eosinophilic gastrointestinal disease, intracranial and cardiac disease managed with Benralizumab

#### Aw, Michael J.^1^, Mir, Adhora^2^, Polito, Vanessa^2^, Cormier, Maxime^3^, Tardio, Natacha^2^

##### ^1^Faculty of Medicine, Department of Internal Medicine, McGill University, Montreal, QC, ^2^Division of Clinical Immunology and Allergy, Department of Medicine, McGill University Health Centre, Montreal, QC, ^3^Respiratory Division, Department of Medicine, McGill University Health Centre, Montreal, QC

*Allergy, Asthma & Clinical Immunology* 2026, **22(2)**: 44

**Background** Hypereosinophilic syndrome (HES) is characterized by elevated blood and tissue eosinophils and eosinophil-mediated organ dysfunction. Specifically, idiopathic HES (iHES) represents a rare variant of exclusion. Presently, we report on a female with iHES and multi-system disease who demonstrated significant clinical and biochemical response to Benralizumab.

**Case Presentation:** A 19-year-old female with longstanding atopy (eczema, allergic rhinitis, food/drug allergy, urticaria, uncontrolled asthma) and complex neonatal history (prematurity at 27 weeks, twin-twin transfusion, cerebral infarct, hydrocephalus) was followed for multisystem eosinophilic disease.

She had persistent hypereosinophilia (peak 4.49 × 10⁹/L), elevated tryptase (13 ng/mL), and IgE (5774 µg/L). Endoscopy confirmed severe eosinophilic gastrointestinal disease (EGID) with 60–100 eosinophils per high power field in the duodenum to esophagus. At age 20, she developed hemianopia with right occipital hyperdensity and non-ischemic cardiomyopathy on magnetic resonance imaging. Bone marrow biopsy showed mild eosinophilia without c-Kit mutation, blasts, FLT3 mutations, or FIP1L1-PDGFRα rearrangement. Autoimmune and infectious workup were negative. A heterozygous pathogenic *MALT1* variant was identified. Serum Antineutrophil Cytoplasmic Antibodies were negative and she had no clinical features suggestive of vasculitis.

Following pulse intravenous steroids, she transitioned to Benralizumab 30 mg SC every 8 weeks for her severe asthma (two years) achieving sustained eosinophil depletion, normalized tryptase, EGID remission, stable imaging, and cardiomyopathy resolution. Asthma has improved but limited by inhaler non-adherence and smoking.

**Conclusions** MALT1 regulates NF-κB signaling with homozygous deficiency linked to combined immunodeficiency or a severely atopic phenotype with elevated IgE and eosinophilia. This patient’s heterozygous genotype would be atypical, although functional testing is in process to rule out a dominant negative phenotype or haploinsufficiency.

This case highlights rare but notable multisystem involvement in iHES, specifically since neurological (~ 4%) and cardiac (5–15%) involvement remains uncommon. Clinicians should consider comprehensive systemic screening in iHES patients. Furthermore, this case supports the sustained benefit of anti-IL5R therapy in the management of iHES-associated complications.

*The patient gave their informed and explicit consent to publish their data in an online, open-access journal.*

## 45

### A case of progressive T-cell lymphopenia and autoimmunity following proctocolectomy

#### Aw, Michael J.^1^, AlYaqout, Fatemah^2^, Polito, Vanessa^2^, Tsoukas, Christos^2^

##### ^1^Faculty of Medicine, Department of Internal Medicine, McGill University, Montreal, QC, ^2^Division of Clinical Immunology and Allergy, Department of Medicine, McGill University Health Centre, Montreal, QC

*Allergy, Asthma & Clinical Immunology* 2026, **22(2)**: 45

**Background** Gut-associated lymphoid tissue (GALT) represents the body’s largest lymphatic reserve and plays a critical role for B-cell class recombination, peripheral tolerance and cellular homeostasis. Extensive bowel resection may cause lymphopenia through multiple mechanisms, particularly in susceptible individuals. It is important to consider secondary causes of lymphopenia peri-operatively.

**Case Presentation:** A 56-year-old male developed T cell lymphopenia and worsening autoimmunity following proctocolectomy for ulcerative colitis (UC) previously managed without immunomodulators. Following his proctocolectomy, he developed worsening vitiligo, pyoderma gangrenosum requiring hospitalization, and subclinical hypothyroidism. He developed progressive, persistent lymphopenia (2.79 × 10^9/L to 0.69 × 10^9/L) and was referred to clinical immunology to assess for primary immune deficiency. Investigations were notable for reduced T cell proportion (55%) specifically CD4 > CD8 (25% and 25% respectively), normal B cell percentage, Ig levels, and vaccine responses. There was no evidence of hepatosplenomegaly or intrabdominal lymphadenopathy. Genetic testing was non-contributory with five heterozygous variants of uncertain significance (VUS), affecting CARD11, RUNX1, STAT5B, ZCCHC8 and KDM1A. Notably, the patient did not experience any functional T cell deficiency or opportunistic infections.

**Conclusions** Mechanisms of post-colectomy lymphopenia include lymphatic dysregulation, active inflammation from underlying pathology, malnutrition, immunosuppressant medication, or peri-operative infections. Active colitis is associated with lymphocyte dysfunction and lymphopenia, however, post-operatively the patient had quiescent UC. Similarly, malnutrition is not known to cause isolated lymphopenia. Alternatively, we hypothesize immune dysregulation following significant GALT resection. Murine models and children with bowel resection show reduced circulating lymphocytes including age-dependent CD4 + T-cell decrease. Although homozygous STAT5B deficiency is associated with colitis and selective T cell deficiency and gain-of-function CARD11mutations are associated with lymphoproliferative disorders, T cell anergy, and autoimmunity, the patient’s variants were non-pathogenic and his presentation was not consistent with these disorders. This case underscores the need to consider secondary causes of lymphopenia and low lymphocyte subsets beyond primary immunodeficiencies in the peri-operative setting.

*The patient gave their informed and explicit consent to publish their data in an online, open-access journal.*

## 46

### Acacia gum hypersensitivity: an elusive cause of persistent multisystem complaints

#### Bou Rjeily, Marianne^1^, Abow-Mohamed, Ikram^1^, Denis-D’Ortun, Samuel^2^, Kwok, Michelle.^3^

##### ^1^Department of Internal Medicine, McGill University Health Center, Montreal, QC, ^2^Independent contributor, Gatineau, QC, 3. Division of Allergy and Immunology, McGill University Health Center, Montreal, QC

*Allergy, Asthma & Clinical Immunology* 2026, **22(2)**: 46

**Background** Acacia gum (E414), derived from Acacia senegal, is a water-soluble emulsifier used in food and pharmaceuticals for its stabilizing properties. Hypersensitivity reactions are mainly reported in occupational settings and are thought to be IgE-mediated, though the specific epitope is unknown No cases of allergic reactions due to ingestion have been documented in the literature.

**Case Presentation:** A 37-year-old man with hypothyroidism and migraines presented with a 10-year history of episodic headaches, weakness, nasal congestion, and presyncope. Extensive investigations, including sinus surgery, immunotherapy, migraine and seizure treatments, and dietary changes, were unsuccessful. His symptoms resolved after discontinuing Synthroid, raising suspicion of an excipient-related reaction. Acacia gum, an inactive ingredient in Synthroid, was identified as a potential trigger. Switching to compounded levothyroxine without acacia gum led to sustained symptom resolution.

A self-administered challenge to acacia gum induced throat tightness, dyspnea, tongue swelling, and near-syncope within 15 min. Skin prick testing was negative to Synthroid and pure levothyroxine. Intradermal testing was positive to Synthroid (6 mm wheal, 12 mm flare) and acacia gum (50 mm wheal), but negative to pure levothyroxine. A placebo-controlled oral challenge to Synthroid induced symptoms 10 min after ingestion of 7.5 mcg (10% of usual dose). Oral challenge to 0.5 g acacia gum reproduced similar symptoms. Serum IgE to acacia gum is pending.

**Conclusions** This case highlights the importance of evaluating excipients as potential allergens in patients with unexplained or multisystem symptoms. Ingestion of acacia gum may cause systemic allergic reactions, even without prior reported cases. Clinicians should be aware of formulation differences between commercial and compounded medications when assessing drug-related reactions.

*The patient gave their informed and explicit consent to publish their data in an online, open-access journal.*

## 47

### Cefazolin-induced anaphylaxis and delayed hypersensitivity in a single patient

#### Bruton Joe, Moss A., Ruiz, Juan

##### The University of British Columbia, Vancouver, BC

*Allergy, Asthma & Clinical Immunology* 2026, **22(2)**: 47

**Background** Hypersensitivity reactions are traditionally classified by the Gel and Coombs system into distinct immune mechanisms, such as Type I (IgE-mediated) and Type IV (T-cell–mediated) reactions. In practice, these categories are often considered mutually exclusive and overlapping immune responses to a single drug are rarely recognized. Cefazolin is the most common cause of perioperative anaphylaxis in North America and is typically associated with Type I reactions, though delayed-type responses have also been documented. Herein we describe, to our knowledge, the first concurrent Type I and Type IV hypersensitivity reactions to cefazolin in a single patient.

**Case Presentation:** A 69-year-old man received cefazolin for prophylaxis during elective total hip arthroplasty. Thirteen days later, he presented with a non-migratory, pruritic rash over his trunk and upper extremities. The distribution and evolution were most consistent with a morbilliform eruption, suggesting a delayed hypersensitivity reaction. Six weeks later, during a planned revision arthroplasty, he experienced intraoperative anaphylaxis within five minutes of receiving cefazolin. He developed facial flushing, angioedema of the lips and tongue, and bronchospasm with elevated airway pressures. He was treated with intravenous epinephrine, antihistamines, corticosteroids, and bronchodilators. The surgery was aborted and successfully completed the following day with an alternative antibiotic. Intradermal testing revealed immediate reactivity to cefazolin (13 mm and 24 mm wheals at 10 mg/mL and 33 mg/mL concentrations respectively), consistent with IgE-mediated sensitization. The following day, the patient developed an indurated, blistering eruption at the cefazolin test site, with a similar eruption appearing days later on the buttocks—suggesting a delayed-type hypersensitivity response to cefazolin.

**Conclusions** This case represents a rare example of both IgE-mediated and delayed T-cell–mediated hypersensitivity to cefazolin in a single patient, highlighting that multiple mechanisms can occasionally coexist. Though overlapping reactions are uncommon, they may explain atypical presentations and offer insight into the complexity of immune responses to perioperative drugs.

*The patient gave their informed and explicit consent to publish their data in an online, open-access journal.*

## 48

### 22q11.2 microduplication syndrome presenting as CVID in an adult patient: a case report

#### Chen, Vivian, Wong-Pack, Andrew, Garkaby, Jenny, Brager, Rae, Cyr, Michael

##### McMaster University, Hamilton, ON

*Allergy, Asthma & Clinical Immunology* 2026, **22(2)**: 48

**Background** Chromosome 22q11.2 is a structurally complex region consisting of low copy repeats (LCR) susceptible to nonallelic homologous recombination, resulting in frequent genomic rearrangements including deletions and duplications. While 22q11.2 deletion syndrome (22q11.2 DS) is well-characterized and commonly associated with immunodeficiency, 22q11.2 duplication syndrome is less frequently identified given its broad phenotypic variability. TBX1 is a gene located at the 22q11.2 locus with a central role in thymic development and contributes to immune dysfunction in 22q11.2 DS. However, immune defects in 22q11.2 duplication syndrome are less well defined. We report the first documented case of a common variable immunodeficiency (CVID) presentation associated with 22q11.2 duplication in an adult.

**Case Presentation:** The proband (P1) is a 49-year-old male with a history of recurrent sinopulmonary infections, gastrointestinal giardia infections, and chronic splenomegaly. He was initially diagnosed as CVID with agammaglobulinemia and treated with immunoglobulin replacement therapy. Due to a family history of hypogammaglobulinemia in his mother, genetic testing was pursued. He was found to have a heterozygous pathogenic 2.52 Mb duplication spanning the 22q11.2 proximal A-D region, including TBX1 associated with 22q11.2 microduplication syndrome. The patient was also found to have low switched memory B cells and normal baseline B and T cells. He has no other features typically associated with 22q11.2 microduplication syndrome. Parental segregation analysis is pending.

**Conclusions** This case expands the recognized clinical spectrum of immunodeficiency associated with 22q11.2 duplication syndrome including a CVID like presentation identified in an adult patient. Given its variable expressivity, incomplete penetrance, and diverse manifestations, 22q11.2 duplication may be underrecognized. Genetic testing should be considered in many cases of CVID, particularly when associated with a family history of immunodeficiency or immune dysregulation. A genetic diagnosis should be considered in these patients, even in the absence of overt physical or developmental features typically associated with syndromic conditions.

*The patient gave their informed and explicit consent to publish their data in an online, open-access journal.*

## 49

### Two cases of anaphylaxis to fenugreek in patients with peanut allergies

#### Chung, Jason^1^, Jeimy, Samira^1^, Cameron, Scott B.^2, 3^, Abrams, Elissa M.^2, 4^, Cook, Victoria E.^2, 3^

##### ^1^Division of Clinical Immunology and Allergy, Department of Medicine, Western University, London, ON, ^2^Division of Clinical Immunology and Allergy, Department of Pediatrics, University of British Columbia, Vancouver, BC, ^3^Community Immunology and Allergy Clinic, Victoria, BC, 4. Department of Pediatrics, Section of Allergy, University of Manitoba, Winnipeg, MB

*Allergy, Asthma & Clinical Immunology* 2026, **22(2)**: 49

**Background** Fenugreek is a legume ubiquitous in Indian and Middle Eastern cuisines, often used in curries, spice blends, and as complementary medicine. Despite its common use, fenugreek allergy is rarely recognized, partly because it’s listed only as a component of “spices” on food labels. This makes avoidance difficult and increases the risk of potentially life-threatening reactions, especially for patients with peanut allergy due to cross-reactivity. Informed consent was obtained from the parents of the patients in this study, following the principals of the Decleration of Helsinki.

**Case Presentation:** We present two pediatric patients with peanut anaphylaxis who experienced hypersensitivity to fenugreek-containing curries.

- **Case 1:** A 14-year-old boy with peanut allergy had two episodes of angioedema, throat constriction, vomiting, and abdominal pain after consuming curry. Fenugreek skin prick test (SPT) was positive (10 mm wheal). The family opted for strict avoidance.
- **Case 2:** A 13-year-old girl with atopic dermatitis and peanut allergy developed immediate throat symptoms, nausea, and heartburn after curry ingestion. Fenugreek SPT was 17 mm. She began oral immunotherapy (OIT) for peanut and fenugreek. Fenugreek’s flavor was very strong, which led to limiting top dose for OIT to 80 mg of protein. Anaphylaxis to OIT occurred at 80 mg of peanut and fenugreek occurring 90 min after the dose, with symptoms of tight chest, and abdominal pain which resolved with epinephrine. She subsequently discontinued OIT.

**Conclusions** These cases highlight fenugreek as a hidden but potentially life-threatening allergen in children with peanut allergy. Retrospective studies have identified up to 10% fenugreek allergy among peanut-allergic patients^1^, suggesting this condition is underrecognized. Clinicians should consider fenugreek allergy in unexplained food-related reactions in legume-sensitized patients, and improved labeling is urgently needed to safeguard allergic individuals.

*The guardians of both patients gave their informed and explicit consent to publish their data in an online, open-access journal.*

## 50

### Rambutan anaphylaxis: a case report

#### Du, Yue^1^, Elsayed, Salma^2^, Sussman, Gordon^3^

##### ^1^Internal Medicine Program, McMaster University, Hamilton, ON, ^2^Queens University, Kingston, ON, ^3^Clinical Immunology and Allergy, University of Toronto, Toronto, ON

*Allergy, Asthma & Clinical Immunology* 2026, **22(2)**: 50

**Background**: Anaphylaxis is a serious, systemic reaction that is potentially fatal. As Canada’s population becomes increasingly diverse, so too does the spectrum of foods available for consumption. This has expanded the range of potential food allergens encountered in clinical practice. However, current awareness of food allergy remains largely centered on a core group of common food allergens.

Rambutan, or Nephelium lappaceum, belongs to the Sapindaceae family, alongside other fruits such as lychee and longan. While popular in many cultural communities and increasingly available in grocery stores across Canada, rambutan is not widely recognized as an allergen.

**Case Presentation: W**e described a case of a 25 year-old female patient who experienced anaphylaxis to rambutan. The patient reported that within minutes of eating rambutan for the first time, she developed rhinorrhea, tightness of throat and angioedema of the face. Oral antihistamines were immediately taken and the reaction resolved within 4 h without emergency medical support. No cofactors were identified.

The patient consumed lychee once prior without reaction. She had no previous food allergies, asthma or eczema. She did have a history of allergic rhinoconjunctivitis in the spring to fall.

We performed skin prick testing on fresh rambutan which demonstrated a 10 mm response with appropriate controls. As her clinical history and testing was consistent with anaphylaxis to rambutan, we recommended avoidance of rambutan and other fruits in the same family including logan and lychee, and to carry an epinephrine autoinjector.

**Conclusions** Overall, we present the first reported case of rambutan anaphylaxis in Canada. Reports of allergic reactions to rambutan have been rarely described, which may contribute to diagnostic delay when reactions occur. As providers for a diversifying population, it is essential to incorporate culturally informed dietary histories into allergy assessments and to recognize that any food can trigger a life-threatening allergic reaction.

*The patient gave their informed and explicit consent to publish their data in an online, open-access journal.*

## 51

### Dupilumab dilemma in severe atopic dermatitis with T-Cell clonality: balancing therapeutic benefit against potential link to cutaneous lymphoma

#### Eldaba, Mariam, AlYaqout, Fatemah

##### McGill University, Montreal, QC

*Allergy, Asthma & Clinical Immunology* 2026, **22(2)**: 51

**Background** Recent evidence suggests dupilumab may unmask or accelerate cutaneous T-cell lymphoma (CTCL) in atopic dermatitis (AD) patients. We present a therapeutic dilemma in a patient with severe, refractory AD and concerning immunologic features.

**Case Presentation:** A 61-year-old woman with adult-onset AD (since age 31) presents with dramatic worsening of eczema since October 2024, now involving 80% BSA with extensive lichenification, excoriations, and secondary infection. Despite high-potency topical corticosteroids and emollients, her disease remains uncontrolled. IgE levels correlate with eczema severity, peaking at 16,985 µg/L and stabilizing around 7,000 µg/L.

In 2015, the patient developed acute necrotizing retinitis with VZV retinitis. Workup revealed normal IgG, IgA, and B cell profile, profound CD8 + lymphopenia (< 50 cells/µL), elevated CD4:CD8 ratio (> 100:1), progressive hypereosinophilia (1.97 → 3.79 × 10⁹/L over 3 months), and worsening EBV viremia (2,951 → 5,495 copies/mL). T-cell receptor clonality is detected in both peripheral blood and bone marrow, though repeated biopsies show no morphologic lymphoma, and PET imaging is negative.

While dupilumab is indicated for her severe, refractory AD, recent studies raise potential safety concerns. Hasan et al. (2024) demonstrated a 4.1-fold increased CTCL risk in dupilumab-treated AD patients. Li et al. (2025) reviewed 124 cases of dupilumab-associated lymphoproliferative disorders, with median onset at 5 months. Our patient exhibits multiple high-risk features: adult-onset AD, pre-existing T-cell clonality, and hypereosinophilia—all identified as risk factors for dupilumab-associated CTCL. It is hypothesized that dupilumab does not directly cause CTCL but may unmask pre-existing disease, or possibly lead to Th2 cytokine skewing, resulting in progression of existing abnormalities. Given the difficulties in diagnosing CTCL and the overlapping features with eczema, these studies likely uncover a correlation rather than causation, but mechanistic studies are ongoing.

**Conclusions** This case exemplifies the challenging risk–benefit analysis when severe AD significantly impairs quality of life, but concerning immunologic features suggest high lymphoma risk with dupilumab.

*The patient gave their informed and explicit consent to publish their data in an online, open-access journal.*

## 52

### Recognizing LTP allergy in Canada: a case series highlighting an underrecognized cause of food anaphylaxis

#### Elsayed, Salma^1^, Du, Jennifer^2^, Moskovic, Erin^1^, Sussman, Gordon^1^

##### ^1^Gordon Sussman Clinical Research Inc., Toronto, ON, ^2^McMaster University, Hamilton, ON

*Allergy, Asthma & Clinical Immunology* 2026, **22(2)**: 52

**Background** Lipid transfer protein (LTP) allergy is an increasingly prevalent cause of food-induced anaphylaxis worldwide. LTPs are heat-stable, digestion-resistant plant proteins found in a variety of fruits, vegetables, nuts, and legumes. Pru p 3, the major peach LTP, is a frequent trigger of severe reactions. While ubiquitous in Southern Europe, LTP allergy remains underdiagnosed in Canada, and often mistaken for oral allergy syndrome (OAS) which typically causes mild, localized symptoms. We present three Canadian adults with LTP allergy.

**Case Presentation: Patient 1**, a 63-year-old woman, experienced anaphylaxis after consuming Pad Thai, requiring epinephrine. Symptoms included urticaria, angioedema, difficulty swallowing, and dysphonia. No cofactors were identified. She also reported OAS to raw fruits. Laboratory results revealed elevated Pru p 3-specific IgE (26.10 kU/L).

**Patient 2,** a 30-year-old woman, experienced three anaphylactic reactions within one year. The most severe occurred after consuming a squash salad, with ibuprofen use and exercise as cofactors. Additional episodes followed with blueberries and Caesar salad. Symptoms included urticaria, angioedema, and hypotension, requiring epinephrine. Pru p 3-specific IgE was elevated (8.56 kU/L). She was started on omalizumab due to reaction severity.

**Patient 3,** a 40-year-old woman, experienced a systemic reaction after consuming salad dressing containing canola oil, followed by exercise as a cofactor. The reaction was confirmed by a food challenge. Symptoms included urticaria, angioedema, and lightheadedness. Pru p 3-specific IgE was positive (7.57 kU/L).

All patients were prescribed an epinephrine autoinjector and avoidance strategies were discussed.

**Conclusions** We present these cases to highlight the importance of recognizing LTP allergy in Canada as a cause of severe food-related anaphylaxis. Timely diagnosis is critical for appropriate management and prevention of potentially life-threatening reactions.

*All three patients gave their informed and explicit consent to publish their data in an online, open-access journal.*

## 53

### Dupilumab as a therapeutic option in omalizumab-refractory chronic spontaneous urticaria: a real-world case series

#### Elsayed, Salma, Moskovic, Erin, Sussman, Gordon

##### Gordon Sussman Clinical Research Inc., Toronto, ON

*Allergy, Asthma & Clinical Immunology* 2026, **22(2)**: 53

**Background** Chronic Spontaneous Urticaria (CSU) is characterized by persistent wheals, erythema, angioedema, and pruritus lasting longer than six weeks, thought to be autoimmune. Second-generation H1-antihistamines are first-line therapy with omalizumab for second-line treatment. However, some patients remain refractory to both treatments. Three phase 3 studies (CUPID) demonstrated dupilumab, an interleukin-4 receptor alpha (IL-4Rα) antagonist, to be effective in managing CSU in antihistamine treatment failures. The CUPID study B did not meet all its primary endpoints in the omalizumab failures. This case series evaluates the clinical outcomes of dupilumab in xolair-refractory CSU patients.

**Case Presentation:** Five patients (four females, one male) aged between 23 and 64 years, presented with recurrent wheals, angioedema, and erythema, characteristic of CSU. All patients were initially treated with omalizumab but showed limited improvement over time as symptoms persisted, and thus they were classified as treatment-refractory. Notably, all patients had low total serum IgE levels (< 30 IU/mL), a biomarker associated with poor omalizumab response. Following omalizumab failure, all patients were subsequently cleared and initiated with dupilumab therapy. Four out of the five patients who failed to respond to omalizumab showed symptom improvement with dupilumab.

**Conclusions** This real-world case series suggests that dupilumab may be an effective treatment option for CSU patients who are refractory to omalizumab. Further research is needed to confirm these findings and the optimal role of dupilumab in CSU management.

## 54

### Severe honeybee venom anaphylaxis in a Canadian beekeeper, with onset after overwintering bees and natural immune tolerance with subsequent stings throughout the summer

#### Hong, Jung Min (Julie)^1^, McKibbin, Lundy^1, 2^

##### ^1^University of Manitoba, Department of Internal Medicine, Section of Adult Allergy and Clinical Immunology, Winnipeg, MB, ^2^University of Manitoba, Department of Pediatrics and Child Health, Section of Pediatric Clinical Immunology and Allergy, Winnipeg, MB

*Allergy, Asthma & Clinical Immunology* 2026, **22(2)**: 54

**Background** We report natural immune tolerance observed in a professional beekeeper who experienced severe anaphylaxis with his first sting of the year–after prolonged overwintering of bees—and subsequent tolerance of stings—typically at least one daily—thereafter and throughout the remainder of the summer.

**Case Presentation:** A 24-year-old healthy male beekeeper presented to the emergency department after a single honeybee sting caused rapid-onset light-headedness, presyncope, nausea, vomiting, diarrhea, and diffuse urticaria. This was his first sting of the season. He had been stung multiple times in prior seasons without experiencing any allergic reactions. Intradermal testing demonstrated monosensitization to honeybee venom, with a 9 mm wheal and a 24 mm flare; testing was negative for other *Hymenoptera* venoms. Baseline tryptase level was normal. He was prescribed multiple EpiPens due to his remote location and the severity of his initial reaction. He went on to receive numerous stings in the following weeks without any severe reactions. Due to the nature of his work, he was offered venom immunotherapy.

**Conclusions** Overwintering of bees is a common practice in colder climates. Canadian Beekeepers may face many consecutive months without any exposure before their first sting of the season. Despite experiencing a severe reaction to the first sting of the year, the patient developed natural tolerance and did not need to use his EpiPens with subsequent stings during warmer months. The possibility of his tolerance waning during the off-season has led him to consider pursuing venom immunotherapy when he is not routinely exposed to bee stings. While beekeepers are known to develop tolerance due to frequent stings, this case highlights the potential implications of prolonged avoidance during overwintering in colder climates and raises awareness of and discussion regarding preventive management.

*The patient gave their informed and explicit consent to publish their data in an online, open-access journal.*

## 55

### Post-transplant outcomes in a pediatric patient with Chronic Granulomatous Disease, Duchenne muscular dystrophy, and McLeod syndrome identified by genetic testing

#### Jeganathan, Kristine, Pham-Huy, Anne

##### Children’s Hospital of Eastern Ontario, Ottawa, ON

*Allergy, Asthma & Clinical Immunology* 2026, **22(2)**: 55

**Background** Chronic granulomatous disease (CGD) is a rare primary immunodeficiency caused by mutations in the CYBB gene, which impair phagocyte production of reactive oxygen species (ROS). In rare cases, large deletions involving CYBB extend to adjacent genes at Xp21.1 to Xp11.3, including DMD, XK, and CFAP47, resulting in diagnoses such as Duchenne muscular dystrophy and McLeod syndrome.

**Case Presentation:** We previously reported a pre-transplant case of a two-year-old male who presented with a Serratia liver abscess and hepatomegaly. Immunologic workup confirmed CGD, and genetic analysis identified a pathogenic X-linked deletion encompassing nine OMIM genes and sixteen coding genes, including CYBB, DMD, XK, and CFAP47. Based on additional clinical features and gene involvement, he was also diagnosed with Duchenne muscular dystrophy and McLeod syndrome.

This report provides an update following allogeneic bone marrow transplant (BMT). The patient received a 10/10 HLA-matched unrelated donor transplant at age three, with conditioning using busulfan, fludarabine, and alemtuzumab. Post-transplant complications included EBV reactivation treated with rituximab and a single episode of cutaneous GVHD that resolved. At day 118 post-transplant, chimerism is currently at 75 percent, with continued immunosuppression on tacrolimus. He is clinically well, seizure-controlled, with good energy and performance status (Lansky score 100).

Genetic findings informed transfusion precautions due to loss of Kx antigen expression associated with McLeod syndrome, and arrangements were made with national blood services. Neurologic and endocrine follow-up continues for Duchenne muscular dystrophy and adrenal insufficiency, respectively.

**Conclusions** This case demonstrates how comprehensive genetic testing can clarify complex diagnoses and inform transplant planning, transfusion safety, and long-term care. Early identification of a syndromic deletion enabled timely coordination of subspecialty follow-up, risk assessment, and post-transplant monitoring. In pediatric immunology, genetic testing and multidisciplinary care play a key role in guiding clinical decisions and improving outcomes for children with rare disorders involving multiple organ systems.

*The patient’s guardian gave their informed and explicit consent to publish their data in an online, open-access journal.*

## 56

### Clinical reactivity to cooked but not raw chickpea: a case report study

#### Kim, Min Jung^1^, LeBlanc, Vicky^2, 3^

##### ^1^Department of Pediatrics, Faculty of Medicine, University of Western Ontario, London, ON, ^2^Division of Allergy, Department of Pediatrics, Faculty of Medicine, University of British Columbia, Vancouver, BC, ^3^British Columbia Children’s Hospital, Vancouver, BC

*Allergy, Asthma & Clinical Immunology* 2026, **22(2)**: 56

**Background** It has been previously reported in the literature that thermal processing can impact the allergenicity of specific foods. This phenomenon has been reported with other allergens including fish and legumes such as peas and soy. It is hypothesized that the heating process can create new allergenic epitopes as well as destroy existing epitopes, resulting in differing reactions to raw versus cooked versions of the same protein. However, the response can be variable depending on the food. Prior molecular studies have suggested that thermal processing decreases IgE binding for lentils and chickpeas specifically. Our case report describes a patient who clinically reacted to and had skin testing positive to cooked, but not raw chickpea.

**Case Presentation:** Our patient is a 2-year-old female with a history of peanut and almond allergy. Upon ingestion of cooked chickpea flour with no other ingredients, she developed oral pruritis, profuse emesis, urticaria and significant lethargy. She was taken to the emergency room but no interventions were required. She was previously tolerating fresh chickpeas, consuming hummus once weekly prior to the reaction, and passed an observed ingestion with hummus. Skin testing was positive to chickpea flour (max wheal diameter of 17 mm) but negative to fresh chickpea (3 mm) and fresh split chickpea (3 mm). Specific IgE was positive to chickpea (25.00 kU/L). She was provided an epinephrine autoinjector and continues to avoid chickpea. There were no other reported ingredients in the flour, but flour analysis is pending to rule out cross-contamination. At this time, she is also planned for initiation on oral immunotherapy, using the cooked chickpea flour.

**Conclusions** To our knowledge, this is the first case report documenting clinical reactivity to processed chickpea flour, but not fresh chickpeas. This further supports that legumes allergies may present differently based on the heating process due to creation of new allergenic epitopes.

*The patient’s guardian gave their informed and explicit consent to publish their data in an online, open-access journal.*

## 57

### New advances in the use of biologics for food allergy & practical limitations in clinical practice

#### Le Blanc, Vicky, Chan, Edmond S., Mak, Raymond

##### University of British Colombia, Vancouver, BC

*Allergy, Asthma & Clinical Immunology* 2026, **22(2)**: 57

**Background** In February 2024, omalizumab became the first medication FDA approved for the treatment of food allergy in the United States, marking a new era in the management of food allergy. This new advance gathered significant attention from the media and patient families. Although no biologic is currently approved for the treatment of food allergy in Canada, a subset of Canadian families is already demonstrating interest in this novel approach.

**Case Presentation:** We describe 2 cases of pediatric patients that demonstrated interest in accessing off label biologics for the treatment of food allergy. First patient was an 11-year-old male with peanut allergy who has a history of severe anaphylaxis during a peanut oral food challenge requiring epinephrine infusion. He was on peanut sublingual immunotherapy (SLIT) at maintenance dose of 4 mg protein for 18 months. Peanut ssIgE remained elevated at 94.9 kU/L. The second patient was a 13-year-old female with multiple food allergies with highly elevated serum specific IgE to egg, peanut and sunflower seeds (> 100 kU/L). Given severe phenotype, both patients desired access to omalizumab as an adjunctive therapy for OIT since FAD announcement. Both patients had elevated total IgE, necessitating omalizumab at 600 mg subcutaneously every 2 weeks based on the dosing chart approved by the FDA. The approved dose would require 8 injections per month for a prolonged period of time, which was unacceptable for those 2 pediatric patients. The out of pocket cost was another obstacle (approximately $75,000 CAD per year) as food allergy is currently an off label indication in Canada.

**Conclusions** Omalizumab for the treatment of food allergy often requires high doses. The availability of new formulations in Canada for children allowing for fewer injections per month would facilitate access for high risk patients whose families are willing to pay out of pocket.

*Both patients’ guardians gave their informed and explicit consent to publish their data in an online, open-access journal.*

## 58

### Late diagnoses of p47phox chronic granulomatous disease: Two inflammatory presentations with large NCF1 deletions

#### Marois, Louis^1, 6^, Dagher, Jason^2^, Fortin, Paul^4, 6^, Wagner, Éric^, 6^, Gauthier, Amélie^1^, Yeganeh, Mehdi^5, 6^, Allard-Chamard, Hugues^3^, Pelletier, Martin^6^, Chetaille, Anne-Laure^4, 6^

##### ^1^Department of immunology-allergy, CHU de Québec—Université Laval, Québec, QC, ^2^Department of Dermatology- CHU Sherbrooke, Sherbrooke, QC, ^3^Division of Rheumatology, CHUS, Sherbrooke, QC, ^4^Division of Rheumatology, CHU de Québec-Université Laval, Québec, QC, ^5^Division of Medical Genetics, CHU de Québec-Université Laval, Québec, QC, ^6^CHU de Québec Research Center—Université Laval, Québec, QC

*Allergy, Asthma & Clinical Immunology* 2026, **22(2)**: 58

**Background** Chronic granulomatous disease (CGD) is a primary immunodeficiency caused by genetic defects affecting NADPH oxidase in phagocytes, impairing the respiratory burst required to kill catalase-positive bacteria and fungi. It usually presents in early childhood with recurrent and severe infections, abscess formation, granulomas, and gastrointestinal inflammation. However, in some individuals, particularly those with p47phox deficiency due to mutations in the *NCF1* gene, symptoms may appear later in life, often dominated by autoimmune or inflammatory manifestations rather than overt infections.

**Case Presentation:** A 58-year-old French-Canadian woman was referred for evaluation of a long-standing inflammatory syndrome. Her medical history included childhood-onset discoid lupus, hidradenitis suppurativa, oral ulcers, and recurrent cutaneous abscesses. She had previously been diagnosed with undifferentiated connective tissue disease and was treated with azathioprine. Despite this, she experienced severe infections, including *Burkholderia cepacia* pneumonia, biopsy-proven inflammatory pachymeningitis, and refractory perianal fistulizing disease without colitis. Neutrophil oxidative burst testing by dihydrorhodamine (DHR) assay showed severely impaired activity, and genetic testing identified a homozygous large deletion in *NCF1*. Azathioprine was discontinued and replaced with ustekinumab to reduce infectious risk. Hematopoietic stem cell transplant (HSCT) evaluation is ongoing.

The second case involves a 48-year-old man with Crohn’s disease, recurrent oral ulcers, and a history of multiple cutaneous squamous cell carcinomas. He similarly exhibited a near-absent oxidative burst and was found to have the same large homozygous *NCF1* deletion.

**Conclusions** These cases illustrate that p47phox-deficient CGD can present in adulthood with atypical features such as chronic inflammation, autoimmunity, and dermatologic involvement. Late diagnosis may delay appropriate management. Immunodeficiencies such as CGD should be included in the differential diagnosis of autoimmune conditions complicated by unusual infections. Early interdisciplinary investigation is essential to guide safe immunomodulation and to prevent severe infectious complications.

*Both patients gave their informed and explicit consent to publish their data in an online, open-access journal.*

## 59

### Seizures and chronic neurodevelopmental outcomes associated with acute food protein-induced enterocolitis syndrome: a case report

#### Martinez, Benjamin^1^, Alhamdi, Shatha^2, 3, 4^, Upton, Julia E. M.^2^

##### ^1^Department of Paediatrics, The Hospital for Sick Children, Toronto, ON, Canada, Toronto, ON, ^2^Division of Immunology and Allergy, Department of Paediatrics, The Hospital for Sick Children, Temerty Faculty of Medicine, University of Toronto, Toronto, ON, ^3^Allergy and Immunology Division, Department of Pediatrics, King Abdulaziz Medical City, Riyadh, Saudi Arabia, ^4^King Abdullah International Medical Research Center (KAIMRC), King Saud Bin Abdulaziz University for Health Sciences, Ministry of National Guard Health Affairs (MNGH), Riyadh, Saudi Arabia

*Allergy, Asthma & Clinical Immunology* 2026, **22(2)**: 59

**Background** Food protein-induced enterocolitis (FPIES) is a non-IgE mediated food allergy that presents with acute episodes of delayed gastrointestinal symptoms after consuming a trigger food. Diagnosis is based on clinical criteria and can be challenging due to a broad differential and absence of a diagnostic test. Although severe consequences, including cardiac arrest, have been reported, there are no known cases with chronic consequences.

**Case Presentation:** We describe the case of an infant with a complicated cardiac history who upon reintroduction to partially hydrolyzed cow’s milk formula (pHF), had a severe acute FPIES episode complicated by shock and hypoxic ischemic brain injury. She was born at term with single-ventricle cardiac physiology requiring surgical correction to double-ventricle physiology, which was complicated by heart block requiring permanent pacemaker implantation. She had a history of feeding intolerance with pHF and extensively hydrolyzed formula supplementation resulting in an expressed breast milk diet via G-tube. At six months, she started a new pHF and developed emesis and diarrhea, resulting in severe dehydration and shock requiring intensive resuscitation with fluids, vasoactive agents, and intubation. Extensive infectious, metabolic, and genetic workup did not yield a diagnostic explanation. A presumptive FPIES diagnosis was made, and she was managed with avoidance of all non-human milk products and soy with gastrointestinal symptom improvement. Her hospitalization was complicated by persistent seizures and encephalopathy with neuroimaging suggestive of hypoxic ischemic injury. Although remaining seizure-free since discharge, she has ongoing neurological concerns for global developmental delay, hypotonia, and bilateral esotropia.

**Conclusions** This case highlights the potential for long-term neurological consequences following an acute FPIES episode complicated by profound shock, albeit in a child with cardiovascular risk. Early recognition of FPIES episodes is crucial in preventing severe consequences, affirming the importance of a dietary history and inclusion of FPIES in the differential diagnosis when appropriate.

*The patient’s guardian gave their informed and explicit consent to publish their data in an online, open-access journal.*

## 60

### Immediate hypersensitivity reaction to human serum albumin after therapeutic paracentesis: a case report

#### McNicholl, Jaime^1^, Salimi, Payam^2^, Mckibbin, Lundy^2, 3^

##### ^1^University of Manitoba, Max Rady College of Medicine, Winnipeg, MB, ^2^Department of Internal Medicine, Section of Adult Allergy and Clinical Immunology, Winnipeg, MB, ^3^Department of Pediatrics and Child Health, Section of Pediatric Allergy and Clinical Immunology, Winnipeg, MB

*Allergy, Asthma & Clinical Immunology* 2026, **22(2)**: 60

**Background** Human serum albumin (HSA) is the most abundant plasma protein and plays an essential role in maintaining oncotic pressure. Clinically, it is commonly administered after large-volume paracentesis in patients with decompensated cirrhosis to prevent circulatory dysfunction. Although hypersensitivity reactions to HSA are exceedingly rare, they warrant consideration in patients who develop acute manifestations during infusion, as such reactions may be underrecognized due to the endogenous origin of the protein.

**Case Presentation:** We describe the case of a 53-year-old man with decompensated cirrhosis and no history of atopy, who developed an immediate pruritic, evanescent and erythematous cutaneous reaction during HSA infusions following large-volume paracentesis. The suspected hypersensitivity was attributed to HSA, as no reaction occurred when paracentesis was repeated without albumin. Due to a lack of standardized protocols for HSA skin testing, we used a commercially accessible alternative: HSA from the sterile diluent used in venom immunotherapy (ALK). This preparation, containing 0.03% human serum albumin and lacking common stabilizers, produced a 10 mm wheal with flare on intradermal testing using appropriate positive and negative controls. Control testing on a healthy volunteer was negative. These findings were consistent with an immediate hypersensitivity reaction to HSA. Given the absence of systemic symptoms or anaphylaxis and the clinical need for continued albumin support, the patient tolerated several albumin infusions with antihistamine pre-treatments.

**Conclusions** Although HSA allergy is exceedingly rare, it should remain in the differential diagnosis for patients who experience adverse reactions during infusions. The endogenous nature of HSA may lead to under-recognition of its allergenic potential, especially as morphological changes introduced during manufacturing may alter immunogenicity. Diagnostic confirmation is feasible using intradermal testing with the sterile albumin-containing diluent used in venom immunotherapy, readily available in allergy clinics. In mild, non-anaphylactic reactions, antihistamine premedication may enble safe continuation of clinically indicated albumin infusions.

*The patient gave their informed and explicit consent to publish their data in an online, open-access journal.*

## 61

### Exacerbation of undifferentiated connective tissue disease with dupilumab therapy

#### Mir, Adhora, Tardio, Natacha

##### McGill University, Montreal, QC

*Allergy, Asthma & Clinical Immunology* 2026, **22(2)**: 61

**Background** Dupilumab, a monoclonal antibody targeting IL-4Rα, suppresses Th2-mediated inflammation in multiple atopic diseases including atopic dermatitis, allergic asthma, and eosinophilic esophagitis. Previous case reports demonstrate development of diseases such as psoriasis, pyoderma gangrenosum, and multiple sclerosis with dupilumab therapy, suggesting a role of dupilumab in immunologic shifting. We describe here the development of undifferentiated connective tissue disease with possible dermatomyositis following dupilumab treatment in a highly atopic patient.

**Case Presentation:** A young male with a history of allergic rhinitis, food allergies, atopic dermatitis, chronic rhinosinusitis with nasal polyposis, eosinophilic esophagitis, and one year of mild Raynaud’s like reaction was treated with dupilumab therapy for atopic dermatitis. The patient developed worsening of the Raynaud’s like reaction during dupilumab infusions. Following 8 months of dupilumab therapy, he developed significant polyarthralgia. Rheumatologic evaluation revealed an active scleroderma pattern and positive anti-MJ antibodies, leading to a diagnosis of undifferentiated connective tissue disease with possible dermatomyositis. Inflammatory symptoms improved but did not resolve following discontinuation of dupilumab, and he was subsequently treated with upadacitinib and hydroxychloroquine.

**Conclusions** This case describes the exacerbation of undifferentiated connective tissue disease, with dermatomyositis characteristics, during dupilumab therapy in a young, highly atopic male with prior mild Raynauds-like symptoms. Symptoms, including Raynaud’s-like reactions and arthralgias, improved after discontinuing dupilumab, suggesting its role in the inflammatory disease. This patients’ prior Raynauds-like symptoms suggest a genetic predisposition for autoimmune disease. Early research suggests that inhibition of IL-4 receptors can exacerbate Th17-driven conditions. It is hypothesized that suppression of Th2-mediated inflammation by dupilumab may promote Th1 and Th17 activity, potentially triggering autoimmune diseases. This case underscores the need for caution when prescribing dupilumab and highlights the need for further studies to understand its potential role in triggering connective tissue diseases.

*The patient or their guardian (where appropriate) gave their informed and explicit consent to publish their data in an online, open-access journal.*

## 62

### Unmasking mimics of anaphylaxis: lessons from a complex case

#### Nadarajah, Kishani, AlYaqout, Fatemah, Genest, Geneviève

##### McGill University, Montreal, QC

*Allergy, Asthma & Clinical Immunology* 2026, **22(2)**: 62

**Background** Anaphylaxis is as a life-threatening systemic allergic reaction with diverse presentations, affecting up to 5.1% of the population. The 2023 practice parameter update of the American Academy of Allergy, Asthma, and Immunology emphasizes that the absence of a universal, practical definition and overlap with mimicking conditions contribute to both underdiagnosis and overdiagnosis of anaphylaxis. In a retrospective review from three Australian pediatric emergency departments, 24 of 211 cases labeled as anaphylaxis failed to meet international criteria. Misdiagnosing anaphylaxis can delay identification of the true underlying condition and hinder appropriate management.

**Case Presentation:** We report a case of a 24-year-old female with systemic lupus erythematosus (SLE), eosinophilic asthma, chronic spontaneous urticaria (CSU), and allergic rhinitis presenting with recurrent episodes of hives, angioedema, dyspnea, and stridor. These episodes were treated repeatedly with intramuscular and intravenous epinephrine for presumed idiopathic anaphylaxis. Over 18 months, she experienced approximately 15 episodes, sometimes requiring supplemental oxygen, despite cessation of NSAIDs for suspected NSAID-exacerbated respiratory disease. Baseline and acute-phase tryptase levels were normal, ruling out systemic mastocytosis; normal C1 inhibitor levels and function excluded bradykinin-mediated hereditary angioedema. Hypocomplementemia (low C4) was attributed to underlying SLE activity. Her episodes were ultimately hypothesized to be asthma exacerbations with possible vocal cord dysfunction, triggering CSU and angioedema under stress. After prednisone and high dose cetirizine did not improve the frequency or severity of the episodes, Benralizumab was initiated to control her asthma. However, two months after initiating Benralizumab, she had another episode and Omalizumab was added to control her CSU. She remains exacerbation-free for over three months.

**Conclusions** This case underscores the importance of systematic differential diagnosis in episodes resembling anaphylaxis. While epinephrine is a critical acute therapy, comprehensive evaluation enables targeted therapeutic interventions, preventing unnecessary emergency treatments and achieving sustainable long-term control.

*The patient gave their informed and explicit consent to publish their data in an online, open-access journal.*

## 63

### Eosinophilic esophagitis presenting with pleural effusion: a rare complication

#### Nouraldeen, Alaa^1^, Tardio, Natacha^2^

##### ^1^Department of Medicine, McGill University Health Centre, Montreal, QC, ^2^Division of Clinical Immunology and Allergy, Department of Medicine, McGill University Health Centre, Montreal, QC

*Allergy, Asthma & Clinical Immunology* 2026, **22(2)**: 63

**Background** Eosinophilic esophagitis (EoE) is a chronic, immune-mediated disease of the esophagus characterized by esophageal dysfunction and eosinophilic histology. While often considered a localized disease, peripheral eosinophilia is noted in a subset of patients. However, pleural involvement, particularly eosinophilic pleural effusion (EPE), is extremely rare and not widely recognized as a disease manifestation and potential complication of EoE.

**Case Presentation:** A 36-year-old man presented with progressive dysphagia, dry cough, orthopnea, and pleuritic chest pain. He had recent mold exposure followed by a viral-like illness. Laboratory testing showed peripheral eosinophilia (AEC 3.2 × 10⁹/L). CT imaging revealed esophageal thickening and left pleural effusion. Thoracentesis confirmed an exudative pleural effusion with 8% eosinophils. Esophagogastroduodenoscopy revealed mucosal changes consistent with eosinophilic esophagitis, confirmed by biopsy with 35 eosinophils/hpf. Extensive workup for infectious, autoimmune, and neoplastic causes was negative. The patient was treated with swallowed budesonide and high dose proton pump inhibitors. Within one week, he experienced near-complete symptom resolution and repeat endoscopy and imaging at three weeks showed full histologic and radiologic remission.

**Conclusions** This case illustrates eosinophilic pleural effusion as a rare systemic manifestation of eosinophilic esophagitis potentially caused by transmural inflammation and eosinophil migration into the pleural space. The rapid response to eosinophilic esophagitis directed therapy further supports a localized Th2-mediated disease process, rather than systemic eosinophilic disease. Eosinophilic esophagitis should be considered as a differential diagnosis for exudative pleural effusions in the presence of esophageal symptoms and peripheral eosinophilia.

*The patient gave their informed and explicit consent to publish their data in an online, open-access journal.*

## 64

### Gluten-free and symptom-free: rethinking chronic spontaneous urticaria in an adult patient with undiagnosed celiac disease

#### Nouraldeen, Alaa^1^, Nadarajah, Kishani^1^, Abow-Mohamed, Ikram^2^, Ben-Shoshan, Moshe^3^, Netchiporouk, Elena^4, 5^, Fein, Michael^6^

##### ^1^Department of Medicine, McGill University Health Centre, Montreal, QC, ^2^Division of Gastroenterology and Hepatology, Department of Medicine, McGill University Health Centre, Montreal, QC, ^3^Division of Pediatric Allergy and Clinical Immunology, McGill University Health Centre, Montreal, QC, ^4^Division of Dermatology, McGill University Health Centre, Montreal, QC, ^5^Centre for Outcomes Research and Evaluation, McGill University Health Centre Research Institute, Montreal, QC, ^6^Division of Clinical Immunology and Allergy, Department of Medicine, McGill University Health Centre, Montreal, QC

*Allergy, Asthma & Clinical Immunology* 2026, **22(2)**: 64

**Background** Celiac disease is increasingly prevalent in patients with chronic spontaneous urticaria (CSU). There is a limited amount of evidence from case reports and series in pediatric populations that instituting a gluten-free diet (GFD) in patients with celiac disease and chronic urticaria may improve the course of chronic urticaria. However, there is insufficient evidence that treating celiac disease alters or improves chronic urticaria in adults.

**Case Presentation:** We report a case of a 34-year-old woman with hypothyroidism referred for recurrent significant post-prandial facial and oral angioedema, and diffuse urticaria without identifiable triggers. She initially reported three episodes requiring in-hospital epinephrine over 2 months, with increasing frequency thereafter. Prick-to-prick skin testing to potential food allergens identified on history (pesto, balsamic vinegar) was negative. Baseline complete blood count, tryptase, C3, and C4 levels were normal. Total IgE was 201 mg/L and anti-TPO was negative. Empiric treatment for CSU with cetirizine 20 to 40 mg daily improved symptoms, but post-prandial abdominal pain and diarrhea emerged. Adding famotidine and montelukast was effective, but tapering led to recurrent urticaria/angioedema episodes.

She later reported an unintentional 40-pound weight loss over 18 months. Serology showed significantly elevated anti-TTG (> 200RU/ml), HLA typing was positive for HLA-DQ2, and duodenal biopsy revealed mild intraepithelial lymphocytosis, confirming celiac disease. Specific-IgE to wheat, rye, and barley were negative.

All symptoms completely resolved on a GFD (including urticaria and angioedema), and all medications were discontinued. Occasional urticaria and oral symptoms recurred with accidental gluten re-exposure.

**Conclusions** To our knowledge, this is the first reported case of confirmed new-onset celiac disease in an adult with resolution of chronic urticaria on a GFD. Although routine testing for celiac disease or empiric GFD is not recommended in chronic urticaria, screening for it based on history in pediatric and adult patients with chronic urticaria is important, as management may improve both conditions.

*The patient gave their informed and explicit consent to publish their data in an online, open-access journal.*

## 65

### Acute Generalized Exanthematous Pustulosis induced by ceftriaxone with tolerance to meropenem: A severe cutaneous adverse reaction case series in critical care

#### Sharma, Monica, Ghebrial, Monica, McKibbin, Lundy

##### University of Manitoba, Department of Internal Medicine, Winnipeg, MB

*Allergy, Asthma & Clinical Immunology* 2026, **22(2)**: 65

**Background** Acute generalized exanthematous pustulosis is a severe cutaneous adverse reaction that carries a mortality risk and often presents due to a drug exposure. Practice parameters advise against use of carbapenems with penicillin or cephalosporin induced severe cutaneous adverse reactions. This creates a diagnostic dilemma regarding appropriate treatment when a critically ill patient reacts to a class of lifesaving agents.

**Case Presentation:** We report two patients with allergist-confirmed ceftriaxone-induced acute generalized exanthematous pustulosis who subsequently tolerated meropenem without adverse reaction while monitored in the intensive care unit.

Case 1: A 37-year-old male with past medical history of obesity hypoventilation syndrome, heart failure, recurrent presentations of hypoxemic respiratory failure, tuberculosis and syphilis in the setting of polysubstance use. This patient had two episodes of acute generalized exanthematous pustulosis in response to ceftriaxone. The first occurred during an admission for hypoxemic respiratory failure and pneumonia. The second occurred after a single day of ceftriaxone for a respiratory infection. In both instances, the reactions resolved with supportive care. Subsequently, the patient tolerated five-day courses of meropenem on two successive admissions for pneumosepsis and cellulitis.

Case 2: A 63 -year-old male with no past medical history transferred from a rural hospital to a tertiary hospital intensive care unit for multi-focal ischemic strokes, seizures and bilateral pulmonary emboli. He developed pneumonia while admitted to critical care, was treated with antimicrobials including ceftriaxone, and developed acute generalized exanthematous pustulosis which resolved with cessation of ceftriaxone. There was no recurrence upon transition to meropenem.

**Conclusions** These cases suggest that patients with ceftriaxone-induced acute generalized exanthematous pustulosis may tolerate carbapenem antimicrobials, which differs from current guidelines. Application is most appropriate when alternative treatment options are limited and when risk of mortality is high from otherwise untreated illness. We warn against extrapolation to other forms of severe cutaneous adverse reaction.

*Both patients gave their informed and explicit consent to publish their data in an online, open-access journal.*

## 66

### Case Report: Insights in rasburicase induced anaphylaxis: potential mediation by Saccharomyces cerevisiae sensitization and a novel rasburicase desensitization protocol

#### Sharma, Monica^1^, Geirnaert, Marc^2^, McKibbin, Lundy^1^

##### ^1^University of Manitoba, Department of Internal Medicine, Winnipeg, MB, ^2^CancerCare Manitoba, Winnipeg, MB

*Allergy, Asthma & Clinical Immunology* 2026, **22(2)**: 66

**Background** Rasburicase is a recombinant urate oxidase enzyme—typically derived from *Saccharomyces cerevisiae* and cloned from *Aspergillus flavus*—used for the prevention and treatment of tumor lysis syndrome in patients with hematologic malignancies and high tumor burden. Rare cases of anaphylaxis have been reported. We hypothesized sensitization to yeast or fungal remnants as the etiology, which—to our knowledge—is a novel proposal. *Saccharomyces cerevisiae*sensitization was demonstrated. The rasburicase manufacturer confirmed that the final rasburicase product does contain *Saccharomyces cerevisiae* protein. We also provide the first proposed rasburicase desensitization protocol for future use.

**Case Presentation:** A 58-year-old male with negative G6PD testing and a history of progressive stage IV diffuse large B-cell lymphoma was admitted to the inpatient oncology ward for chimeric antigen receptor T-cell infusion and given rasburicase prophylactically for his high tumour burden. Twenty minutes following his fourth dose of rasburicase, an acute reaction involving facial angioedema, diffuse skin erythematous patches, nausea, emesis, light-headedness, and rigors occurred. This was favoured to be an anaphylactic reaction as it occurred on the fourth dose. Skin prick testing to commercial extracts was positive to *Saccharomyces cerevisiae* and negative to *Aspergillus fumigatus*. *Aspergillus flavus* was not available for testing. A desensitization protocol was created for future rasburicase hypersensitivity reactions after a literature review revealed no available protocol.

**Conclusions** This case demonstrates the risk of anaphylaxis to rasburicase. We propose *Saccharomyces Cerevisiae* as a potential sensitizing agent in rasburicase anaphylaxis. We also provide a desensitization protocol for future application. We highly recommend larger scale investigation evaluating feasibility of the rasburicase desensitization protocol as well as yeast and fungal excipient sensitization testing in patients who have experience rasburicase induced anaphylaxis.

*The patient gave their informed and explicit consent to publish their data in an online, open-access journal.*

## 67

### Successful desensitization protocol with oral tinidazole in a 34-year-old woman with severe drug allergy to metronidazole and persistent trichomonas vaginalis infection

#### Sheikh, Muhammad H.^1^, Song, Christine^2^

##### ^1^University of British Columbia, Vancouver, BC, ^2^Division of Clinical Allergy and Immunology, Department of Medicine, St. Michael’s Hospital, Toronto, ON, Toronto, ON

*Allergy, Asthma & Clinical Immunology* 2026, **22(2)**: 67

**Background** *Trichomonas vaginalis* infection is a common sexually transmitted infection (STI) that requires treatment with nitroimidazole antibiotics such as metronidazole. However, hypersensitivity reactions to metronidazole may complicate treatment in some patients. The alternative nitroimidazole antibiotic, tinidazole, is similar in structure to metronidazole and cross-reactivity is expected to be high. As such, alternative treatments or drug desensitization may be necessary.

**Case Presentation:** A 34-year-old woman with history of persistent *Trichomonas vaginalis* infection presented with generalized non-bullous fixed drug eruption (FDE) to metronidazole. Attempts to desensitize her to metronidazole were unsuccessful with recurrence and worsening FDE. The patient was then treated with intravaginal boric acid, which failed to clear the infection. Due to the persistence of infection and inability to use metronidazole, she was then successfully desensitized to oral tinidazole in a 12-step protocol, reaching a final dose of 2 g (standard treatment dose). The desensitization was performed under medical supervision, and no adverse reactions were noted during the procedure. After completing the desensitization and full treatment with tinidazole, the patient’s infection was successfully cleared. She did not develop any delayed hypersensitivity reactions.

**Conclusions** This case demonstrates that desensitization to oral tinidazole can be an effective alternative treatment for patients with severe non-IgE mediated drug allergy to metronidazole, particularly in cases of persistent *Trichomonas vaginalis* infection where metronidazole desensitization has failed.

*The patient gave their informed and explicit consent to publish their data in an online, open-access journal.*

## 68

### When breakfast bites back: a case report of bird-egg syndrome

#### Tang, Xinxin, Zhu, Rongbo

##### Western University, London, ON

*Allergy, Asthma & Clinical Immunology* 2026, **22(2)**: 68

**Background** Egg allergy typically arises in childhood with sensitization to egg white proteins such as ovomucoid (Gal d 1) and ovalbumin (Gal d 2). A rarer, adult-onset form termed “bird-egg syndrome**”** involves sensitization to alpha-livetin (Gal d 5) found in egg yolk. The proposed mechanism consists of chronic inhalational exposure to household pet bird antigen, leading to respiratory symptoms. This is followed by oral symptoms from egg yolk consumption.

**Case Presentation:** A 22-year-old female presented for evaluation of egg allergy. She has owned a pet conure for 8 years and had developed a habit of sniffing the bird for comfort over the past year. During this time, she experienced immediate oropharyngeal itching after eating undercooked egg yolk while tolerating egg white. She reported no respiratory symptoms. Skin prick testing was negative for egg white and feathers. Serum-specific IgE was positive to egg yolk (1.00 kU/L) and budgie (0.63 kU/L), but negative to egg white. She was advised to avoid raw egg yolk. Ongoing monitoring [XT1] was offered to assess her symptom trajectory over time.

**Conclusions** This case highlights an atypical presentation of bird-egg syndrome characterized by the absence of preceding respiratory involvement. Unlike previously reported cases, this patient had isolated oropharyngeal pruritus following ingestion without allergic rhinitis or asthma symptoms. These symptoms were temporally linked to routine bird sniffing, suggesting that increased exposure to aerosolized bird dander may have expedited the sensitization process. Although exceedingly rare, bird-egg syndrome should be considered in adults with new-onset egg allergy even in the absence of respiratory symptoms. More research is needed to elucidate the risk factors and natural history of bird-egg syndrome.

*The patient gave their informed and explicit consent to publish their data in an online, open-access journal.*

## 69

### Suspected R1 side chain cross-reactivity following serum-sickness-like reaction to amoxicillin: a case report

#### Unninayar, Dana^1^, Shoshan, Moshe B.^3^, Lanoue, Derek^2^

##### ^1^Department of Internal Medicine, The Ottawa Hospital, Ottawa, ON, ^2^Clinical Immunology and Allergy, The University of Ottawa Faculty of Medicine, Ottawa, ON, ^3^Division of Pediatric Allergy and Clinical Immunology, Department of Pediatrics, McGill University Health Centre, Montreal, QC

*Allergy, Asthma & Clinical Immunology* 2026, **22(2)**: 69

**Background** Serum sickness–like reactions (SSLRs) are delayed hypersensitivity reactions that occur days to weeks after exposure to culprit non-protein antigens. SSLRs are characterized by morbilliform or urticarial rashes, fevers, and arthralgias, without the hypocomplementemia and end-organ involvement seen in serum true sickness.

While cross-reactivity with other beta-lactams after a type I hypersensitivity reaction to amoxicillin is thought to be related to similar R1 side chains (R1SC), cross-reactivity for SSLRs has not been well-described. Herein, we present a case of an SSLR to amoxicillin, a subsequent reaction to an oral challenge with a beta-lactam antibiotic with a similar R1SC, and tolerance of a beta-lactam with a dissimilar R1SC.

**Case Presentation:** A healthy 6-year-old female was treated with oral amoxicillin 250 mg every 8-h for a dental abscess. On Day 7 of treatment, she developed a full-body urticarial eruption with arthralgias and fevers. Symptoms resolved with drug cessation and two doses of dexamethasone. Her bloodwork was unremarkable, with normal CBC, complement levels, and renal function. She was diagnosed with an SSLR, and advised to avoid beta-lactams.

Approximately 3-months after the index reaction, due to parental refusal of an amoxicillin challenge, the patient underwent an oral challenge with cephalexin 250 mg. She developed a delayed-onset urticarial eruption over her elbows and knees 10-h post-challenge. Symptoms resolved within 5-h of receiving diphenhydramine 25 mg. She completed a cefuroxime 250 mg oral challenge 1-month later with no reaction.

**Conclusions** This patient developed an SSLR to amoxicillin and, 3-months later, a delayed reaction to an oral challenge with cephalexin, which shares a similar R1SC with amoxicillin. However, she had no reaction to a challenge with cefuroxime, which has a dissimilar R1SC. Although the exact mechanism of SSLRs remains unclear, this case suggests a potential role for the beta-lactam R1SC in mediating such reactions. Further challenge-based cohort studies are needed to validate this hypothesis.

*The patient’s guardian gave their informed and explicit consent to publish their data in an online, open-access journal.*

## 70

### Common variable immune deficiency and presumed autoimmune encephalitis in a patient Aith novel genetic change in NFKB2

#### Wang, Joanne, Kanani, Amin

##### University of British Columbia, Vancouver, BC

*Allergy, Asthma & Clinical Immunology* 2026, **22(2)**: 70

**Background** NF-κB-pathway is a key regulator of immune responses, activated via canonical and non-canonical pathways. The canonical pathway is stimulated by cytokines and microbial signals to promote inflammatory gene expression, and the non-canonical pathway relies on NIK-mediated processing critical for B-cell maturation^1–3^. *NKFB2* mutations disrupt p100 processing leading to defective B-cell differentiation and T-cell mediated autoimmunity^4^. Heterozygous *NFKB2* mutations are recognized causes for common variable immunodeficiency (CVID) and Deficient Anterior Pituitary with Variable Immune Deficiency (DAVID) syndrome.^4^ A cohort analysis of 35 published cases revealed early-onset primary immunodeficiency (PID), with autoimmunity present in 80% of patients.

**Case Presentation:** A 34-year-old female with presumed autoimmune encephalitis and refractory epilepsy now presents with recurrent sinopulmonary infections. Her neurologic symptoms began in August 2018, requiring previous rituximab, and she now remains on three anti-epileptics. Since September 2023, she has developed recurrent sinusitis confirmed by CT, requiring eight courses of antibiotics. She also reports three episodes of radiographically confirmed pneumonias within the past year. Additional infections include recurrent streptococcal pharyngitis and bacterial conjunctivitis. Investigations revealed pan-hypogammaglobulinemia, non-reactive tetanus and diphtheria vaccine titers, and reduced switched memory B cells. T cell memory subsets were within normal limits. Genetics testing revealed heterozygous *NFKB2* variant: c.2072G > A, p(Gly691Asp), classified as a variant of uncertain significance (VUS).

**Conclusions** *NKFB2* variants are well established causes of PID with several studies analyzing clinical phenotypes on published NFKB2 cases^4,5,5,6^. While one study of 138 *NFKB2* cases found encephalitis in 5% of individuals with C-terminal defects variants, it has not been associated with central truncation genotype – the location of the variant in our patient. Although, direct evidence linking *NFKB2* variants to autoimmune encephalitis is limited, disruption of immune regulation by NFKB2 mutations may predisposition to such autoimmunity. To our knowledge, the above specific VUS and its phenotypic presentation has not been reported in existing literature.

*The patient gave their informed and explicit consent to publish their data in an online, open-access journal.*

## 71

### SCARy Pustules: an atypical and fatal case of acute generalized exanthematous pustulosis rapidly progressing to toxic epidermal necrolysis

#### Yang, Zi Fan^1^, Naert, Karen^2^

##### ^1^Division of Clinical Immunology and Allergy, University of Western Ontario, London, ON, ^2^Department of Pathology and Laboratory Medicine, University of Calgary, Calgary, AB

*Allergy, Asthma & Clinical Immunology* 2026, **22(2)**: 14

**Background** Acute Generalized Exanthematous Pustulosis (AGEP) is a severe cutaneous adverse reaction (SCAR) usually distinguished both clinically and histopathologically from Toxic Epidermal Necrolysis (TEN). There are rare cases in the literature of overlap syndromes that show histopathologic consistency with AGEP, but display clinical features of TEN. In most cases prognosis is good with drug discontinuation and mortality is exceptionally rare.

**Case Presentation:** We detail an unfortunately fatal case of AGEP with progressive systemic TEN like features in an 80 year old male after multiple exposures to beta-lactams first as prophylaxis for a dental cleaning and then as treatment for presumed sepsis from the procedure. The eruption of pustules was followed rapidly by geometric erosions, mucosal involvement, refractory atrial fibrillation, and renal failure. Despite high dose steroids and etanercept, he ultimately developed pneumonia with overwhelming septic shock and passed 10 days post admission, 11 days following the dose of amoxicillin. Despite multiple biopsies consistent with AGEP, his clinical course was more consistent with TEN. We illustrate the challenges in the diagnosis and management with photos and histology and discuss the rationale and evidence behind key clinical decisions in this case.

**Conclusions** Despite repeat histopathology consistent with AGEP, the clinical evolution was more consistent with TEN. Such cases are already rare, with mortality being exceedingly so. Beyond drug discontinuation, both the diagnosis and management of these cases remain challenging. Studying the preceding events, presentation, and response to treatment can better aid in the management and prevention of these severe and rarely lif- threatening reactions.

*The patient’s family/executor gave their informed and explicit consent to publish their data in an online, open-access journal.*

## OTHER ALLERGY/IMMUNOLOGY

## 72

### Implementing telemedicine clinics in northern Quebec to reduce health disparities in access to specialist allergy care

#### Aw, Michael J.^1^, Zhu Shi, Meng^1^, Park, Megan^2^, Fein, Michael^3^, Kwok, Michelle^3^

##### ^1^Faculty of Medicine, Department of Internal Medicine, McGill University, Montreal, QC, ^2^Temerty Faculty of Medicine, University of Toronto, Toronto, ON, ^3^Division of Clinical Immunology and Allergy, Department of Medicine, McGill University Health Center, Montreal, QC

*Allergy, Asthma & Clinical Immunology* 2026, **22(2)**: 72

**Background** Indigenous communities in northern Quebec face significant barriers to accessing specialized healthcare, particularly allergy care. These remote regions, accessible only by air, struggle with high rates of atopic diseases and limited healthcare infrastructure. In Nunavik, residents often travel over 1,400 km to Montreal for advanced care, leading to long wait times, missed appointments, and cultural disconnection. In 2021, Nunavik’s medical travel budget was $124.6 million, highlighting the financial strain of accessing healthcare.^1,2,3^

Telemedicine offers a promising solution, enabling cost-effective and convenient treatment and monitoring, especially for remote populations. Telemedicine has been shown to reduce travel-related costs, improve patient access, and support the management of chronic conditions like allergies. This approach has proven particularly effective for managing allergies remotely, offering both synchronous and asynchronous care options.^4^

**Methods** The Connexion Nordique project, in collaboration with Northern community clinics, piloted a monthly telemedicine clinic at McGill University Health Centre to improve access to allergy care.

**Results** Between 2020 and 2023, Cree and Nunavik patient no-show rates for Allergy appointments were 30% and 39% respectively versus 8.3% for local Montreal patients. Between June 2024 and May 2025, 103 patients from Cree and 34 from Inuit communities were seen for dedicated telemedicine consultations (94%) and follow-up. Attendance rates were 76%, with 94% of consultations completed virtually. 79% of patients required follow-up, and 26% of follow-up care was provided in-person.

**Conclusions** Telemedicine has proven to be a cost-effective solution to reduce wait times and improve specialist access for remote areas. While asthma-related consultations were notably absent, food and drug allergies were the primary concerns, suggesting either increased awareness or a higher underlying prevalence. This initiative addresses critical gaps in allergy care through telemedicine and strong community partnerships. Preliminary data indicate a need for further studies to assess disease prevalence and healthcare access more comprehensively in Northern Indigenous communities.

**References**

1. Nunavik Regional Board of Health and Social Services. Annual Report 2023–2024.
2. Ministère de la Santé et des Services sociaux du Québec. Rapport sur les dépenses en santé au Québec, 2022–2023.
3. Canadian Institute for Health Information. Transportation and Health Access in Remote Communities, 2021.
4. EAACI Telemedicine Task Force. Telemedicine with special focus on allergic diseases and asthma: EAACI position paper. *Allergy*. 2023 Apr;78(4):865–76. 10.1111/all.15964

## 73

### Patient and caregiver perspectives on atopic dermatitis treatments and practical considerations: a qualitative focus group study

#### Chen, Lina^1^, Zhao, Irene^1^, Chu, Derek^2^

##### ^1^McMaster University, Hamilton, ON, ^2^Evidence in Allergy Group, Department of Medicine and Department of Health Research Methods, Evidence, and Impact, McMaster University, Hamilton, ON

*Allergy, Asthma & Clinical Immunology* 2026, **22(2)**: 73

**Background** While the lived experience of atopic dermatitis (AD) is well described, less is known about patients’ and caregivers’ perspectives on the growing range of available treatments. This study explores these views to inform how clinicians can better tailor treatment discussions to patients’ practical needs.

**Methods** As part of developing updated AD treatment guidelines, we recruited individuals with AD or their caregivers from Canada and the United States for virtual focus groups. Ethics approval was obtained, and recruitment emphasized diversity and inclusion. Participants reflected on their experiences using treatments including topical steroids, calcineurin inhibitors, PDE4 inhibitors, biologics, immunosuppressants, allergen immunotherapy, antihistamines, wet wraps, and bleach baths. Discussions focused on practical considerations such as side effects, accessibility, time burden, and how treatments affect daily activities. Transcripts were analyzed using reflexive thematic analysis.

**Results** Ten participants (eight adults and two caregivers of children) took part in three focus groups. Three themes emerged: (1) Trial and error in finding tolerable regimens – Participants described a frustrating and prolonged process of identifying treatments that were both effective and manageable. When treatment fatigue arises, reassessing burden and adapting regimens may support better adherence. (2) Evolving treatment needs – Management decisions shifted with perceived symptom changes, life events, and access to therapies such as biologics. Clinicians may routinely revisit treatment goals to align with patients’ changing needs. (3) Psychosocial burden of treatment – Visible symptoms and time-intensive routines contributed to stigma, disrupted routines, and emotional strain. Participants appreciated when clinicians acknowledged these challenges and explored ways to reduce them.

**Conclusions** These findings highlight that patient-centred AD care involves more than prescribing effective therapies. A meaningful collaborative approach requires understanding patients’ lived experiences and adapting care plans to support real-world treatment use.

## 74

### Investigating Canadian allergist and immunologist burnout and clinical practice during the COVID-19 pandemic

#### Housefather, Max^1, 3^, Burrows, Alyssa G.^1^, Hossenbaccus, Lubnaa^1, 2^, Garvey, Sarah^1^, Botting, Hannah^1^, Steacy, Lisa M.^1^, Ellis, Anne K.^1, 2, 3^

##### ^1^Allergy Research Unit, Kingston Health Sciences Centre—KGH Site, Kingston, ON, ^2^Department of Medicine, Queen’s University, Kingston, ON, ^3^Department of Biomedical and Molecular Sciences, Queen’s University, Kingston, ON

*Allergy, Asthma & Clinical Immunology* 2026, **22(2)**: 74

**Background** The COVID-19 pandemic exacerbated physician burnout across Canada and globally, with suspected negative implications for physician stress, health system costs, and patient care [1]. However, the impact of the COVID-19 pandemic on burnout specifically amongst allergists/immunologists has not been described.

**Methods** A cross-sectional survey including questionnaires to capture demographics, practice characteristics, and burnout was electronically distributed to members of the Canadian Society of Allergy and Clinical Immunology between June and October 2022 using Qualtrics XM. Burnout was assessed using the validated Maslach Burnout Inventory Human Services Survey for Medical Personal (MBI-HSS (MP)), which measures Emotional Exhaustion, Depersonalization, and Personal Accomplishment. Demographics and clinical practice data were assessed descriptively. Intergroup differences in burnout metrics were analyzed through Kruskal–Wallis or Mann–Whitney tests with a significance threshold of 0.05.

**Results** One hundred eighty-three allergists/immunologists practicing in Canada responded to the survey. High Emotional Exhaustion (≥ 27/54) and Depersonalization (≥ 10/30) scores were reported by 47% of respondents. There were no significant sex or gender differences in MBI-HSS (MP) scores (p > 0.05). MBI-HSS (MP) scores were highest among respondents 35–44 years of age, 3–5 years into practice, and working at administrative/non-academic centres. Subgroup differences in MBI-HSS (MP) scores were also identified based on racial origin, province of practice, and primary payment method. Challenges practicing during the pandemic, including disruption of in-person assessments, limitations of telehealth, and high patient volumes resulted in increased stress among 65% of respondents. Increased referrals (≥ 61%) and burdensome communications (62%) regarding drug/vaccine allergy were also reported.

**Conclusions** Through our survey, which captured over 80% of allergists and immunologists nationally, we report that Canadian allergists and immunologists experienced unique stressors resulting in substantial rates of burnout during the COVID-19 pandemic. By identifying specialty-specific factors contributing to burnout and population subgroups at increased risk, this study can inform targeted interventions to promote physician wellness in allergy and immunology.

**References**

1. Macaron MM, Segun-Omosehin OA, Matar RH, Beran A, Nakanishi H, Than CA, et al. **A systematic review and meta analysis on burnout in physicians during the COVID-19 pandemic: A hidden healthcare crisis.** *Front Psychiatry*. 2023;**13**:1071397.

## 75

### Penicillin allergy delabelling in pregnancy in a community allergy clinic

#### Jeganathan, Kristine^1^, Power, Alicia^2^, Jagdis, Amanda^3^, Cook, Victoria^4^

##### ^1^Children’s Hospital of Eastern Ontario, Ottawa, ON, ^2^Grow Health Maternity Clinic, Victoria, BC, ^3^Community Allergy Clinic, Island Health Authority, Victoria, BC, ^4^Division of Allergy, Department of Pediatrics, University of British Columbia, Vancouver, BC

*Allergy, Asthma & Clinical Immunology* 2026, **22(2)**: 75

**Background** Penicillin allergy is one of the most frequently reported drug allergies, yet over 90% of individuals with this label are not truly allergic. In pregnancy, penicillin allergy labels can result in increased use of broad-spectrum antibiotics, prolonged hospital stays, and worsened neonatal outcomes in the context of Group B Streptococcus prophylaxis. While growing evidence supports the safety of penicillin allergy delabeling during pregnancy in low-risk patients, most existing protocols are hospital-based, relying solely on the availability of fetal monitoring. Consequently, uptake in community settings remains limited despite the low risk of adverse reactions. Here we describe outcomes of penicillin allergy delabeling in pregnancy in a community allergy clinic in the Island Health Authority of BC. This was completed as part of a registered Quality Improvement (QI) project within the Island Health Authority.

**Methods** From May 2022—June 2025 pregnant people referred to a community allergy clinic underwent assessment for penicillin allergy, including application of the PEN-FAST risk stratification tool and intradermal testing. Patients with PEN-FAST scores of 0–3 and negative intradermal testing were offered direct oral challenge (DOC) with amoxicillin 250 mg followed by a 1-h period of observation. Descriptive analyses were performed using Microsoft Excel.

**Results** A total of 53 patients underwent assessment. One patient had tolerated amoxicillin since the initial reaction and was delabeled without further assessment. 46 (86.8%) had PEN-FAST scores of 0–3. All 46 had negative intradermal testing and underwent DOC without any acute reaction. A single patient (2.2%) reported delayed symptoms, with loose stool that had onset 24 h following the challenge. No patients required epinephrine or transfer to hospital.

**Conclusions** Penicillin allergy delabeling in the third trimester can be safely and effectively conducted in a community clinic setting. Broader implementation of this approach may improve maternal and neonatal outcomes by increasing access to first-line antibiotic therapy.

**References**

1. Atkinson A, Poliquin V, Elwood C. Penicillin allergy delabelling in pregnancy. CMAJ. 2023 Mar 27;195(12):E452–E453
2. Desai SH, Kaplan MS, Chen Q, Macy EM. Morbidity in pregnant women associated with unverified penicillin allergies, antibiotic use, and group B Streptococcus infections. Perm J. 2017;21:16–080.
3. Furness A, Kalicinsky C, Rosenfield L, Punekar YS, Warrington R. Penicillin skin testing, challenge, and desensitization in pregnancy: a systematic review. J Obstet Gynaecol Can. 2020;42(11):1254–61.e3.
4. Wolfson AR, Mancini CM, Banerji A, Larenas-Linnemann D, Picard M, Hong K, et al. Penicillin allergy assessment in pregnancy: safety and impact on antibiotic use. J Allergy Clin Immunol Pract. 2021;9(3):1338–46.
5. Matteson KA, Lievense SP, Catanzaro B, Phipps MG. Intrapartum group B streptococci prophylaxis in patients reporting a penicillin allergy. Obstet Gynecol. 2008;111(2 Pt 1):356–64.
6. Kirven J, Beddow D, Patel L, Carroll C, Kinrade E, Al Wattar BH. Outcomes in reported penicillin allergic mothers and neonates requiring Group B streptococcal prophylaxis: a retrospective observational cohort study. BMC Pediatr. 2021;21(1):327.
7. Mak R, Zhang BY, Paquette V, Kim H. Safety of direct oral challenge to amoxicillin in pregnant patients at a Canadian tertiary hospital. J Allergy Clin Immunol Pract. 2022;10(6):1919–21.e1.

## 76

### Medication allergy testing hub (MATH)—creating an online database for drug allergy testing

#### Knox, Christopher^1^, Krishnan, Sinthiha^2^, Song, Christine^3^, Vadas, Peter^3^, Betschel, Stephen^3^, Phillips, Elizabeth^4^, Lee, Erika^3^

##### ^1^Internal Medicine Program, University of Toronto, Toronto, ON, ^2^Faculty of Medicine, University of Toronto, Toronto, ON, ^3^Division of Clinical Immunology and Allergy, St. Michael’s Hospital, Toronto, ON, ^4^Pharmacology & Pathology, Microbiology & Immunology, Vanderbilt University Medical Center, Nashville, TN, USA

*Allergy, Asthma & Clinical Immunology* 2026, **22(2)**: 76

**Background** Drug allergy skin testing, including skin prick testing (SPT), intradermal testing (IDT), and patch testing (PT), is a valuable clinical tool to assess immediate and delayed drug hypersensitivity reactions.[1] Currently there is no central database with published non-irritating concentrations of drugs available for skin testing.

**Methods** Since 2021, we began creating a database using non-irritating concentrations recommended in “Practical Guidance for the Evaluation and Management of Drug Hypersensitivity: Specific Drugs.”[2] We expanded the database by performing a comprehensive literature search annually in Medline and EMBASE from inception to December 2024 to collect any newly published drug allergy testing protocols. To allow easy database access, we created an online website [https://www.drugallergy.ca/], which also enables feedback from users. Finally, we conducted a survey locally to collect anonymous feedback regarding website functionality.

**Results** To date, our database has over 600 peer-reviewed articles that includes drug allergy testing information, and over 500 drugs with testing concentrations for SPT, IDT and PT. We also provided references to published desensitization protocols for many of these drugs and information on cross-reactivity if available. The website is fully functional and acts as a dynamic resource allowing allergists to submit newly published non-irritating concentrations to the website. Our survey included eight respondents of which 87.5% found the website easy to navigate and a helpful tool in drug allergy management. Our database is updated annually and each year approximately 50 new articles are included.

**Conclusions** We have developed a website that provides drug allergy testing information sourced from peer-reviewed articles, with the goal of disseminating it on a larger scale to enhance accessibility and utility for allergists and pharmacists. The website is designed to be a dynamic resource embracing quality improvement, regularly updated with emerging data to support drug allergy management, and we are planning a mobile version to facilitate convenient access.

**References**

1. Wood RA, Camargo CA, Lieberman P, Sampson HA, Schwartz LB, Zitt M, et al. Anaphylaxis in America: the prevalence and characteristics of anaphylaxis in the United States. *J Allergy Clin Immunol*. 2014;133(2):461–7. 10.1016/j.jaci.2013.08.016
2. Broyles AD, Banerji A, Barmettler S, et al. Practical Guidance for the Evaluation and Management of Drug Hypersensitivity: Specific Drugs. *J Allergy Clin Immunol Pract*. 2020;8(9S):S16-S116. 10.1016/j.jaip.2020.08.006

## 77

### Exploring intramuscular drug challenge in patients with suspected parenteral cephalosporin allergy

#### Zhang, Kevin K.^1^, Wang, Chloe^2^, Yang, Sabrina^3^, Campbell, Jackie^4^, Fu, Lisa^4^, Moolani-Merchant, Yasmin^4^, Song, Christine^5^, Lee, Erika^4, 5^

##### ^1^Temerty Faculty of Medicine, Toronto, ON, ^2^Schulich School of Medicine & Dentistry, London, ON, ^3^Faculty of Medicine, University of Ottawa, Ottawa, ON, ^4^Drug Allergy Clinic, Sunnybrook Health Sciences Centre, Toronto, ON, ^5^Division of Allergy & Immunology, Department of Medicine, University of Toronto, Toronto, ON

*Allergy, Asthma & Clinical Immunology* 2026, **22(2)**: 77

**Background** Drug challenge (DC) is the gold standard for drug allergy evaluation. Conventionally, intravenous (IV) DC is reserved for drugs available only in the parenteral form, as it requires more time and nursing support compared to oral DC. Since certain parenteral antibiotics can be given via an intramuscular (IM) route, we investigated the safety and efficacy of IM challenge for cefazolin and ceftriaxone.

**Methods** We conducted a retrospective chart review of patients who underwent IM DC to cephalosporins at an academic drug allergy clinic between January 2023 and December 2024. Data on demographics, index reactions, and testing outcomes were extracted using REDCap.

**Results** The study included 59 patients (mean age = 51.7, SD 19.4; 61.0% female) with suspected cefazolin (n = 27, 45.8%), ceftriaxone (n = 22, 37.3%), or unknown IV cephalosporin allergy (n = 10, 16.9%). The index reactions were immediate (n = 35, 59.3%), delayed (n = 15, 25.4%), or unknown (n = 9, 15.3%). All patients had negative intradermal skin tests and thus underwent IM challenge to either the culprit or alternative cephalosporin. Most patients (n = 53, 89.8%) passed and had their allergy label removed. Of the remaining patients, 4 (6.8%) had inconclusive results, and 2 (3.4%) had immediate reactions, with one patient having local urticaria and another having severe nausea, akin to the index reactions. These two patients did not proceed to the second step of the DC due to the concern for more severe reactions with a higher dose. Symptoms resolved without complication in under 4 h.

**Conclusions** Our study demonstrated that IM antibiotic challenge is a safe and effective alternative to IV challenge after negative IDT in patients with suspected cefazolin or ceftriaxone allergy. These findings support the use of IM challenges as a viable option for cephalosporin allergy delabelling in appropriate clinical settings.

## 78

### Effectiveness of a provincial policy to improve epinephrine use for anaphylaxis in schools in Alberta, Canada: a pre-post study

#### Najem, Joseph^1^, Ton That, Alexandre^1^, Prosty, Connor^1^, Clarke, Ann E.^9^, Morris, Judy^2^, Gravel, Jocelyn^3^, Lim, Rodrick^4^, Chan, Edmond S.^5^, Goldman, Ran D.^5^, O’Keefe, Andrew^6^, Gerdts, Jennifer^10^, Chu, Derek K.^7^, Upton, Julia^8^, Hochstadter, Elana^8^, Moisan, Jocelyn^11^, Bretholz, Adam^12^, McCusker, Christine^13^, Zhang, Xun^15^, Protudjer, Jennifer L.^14^, Abrams, Elissa M.^16^, Simons, Elinor^16^, Dixon, Andrew^17^, Ben-Shoshan, Moshe^18^

##### ^1^McGill University, Montreal, QC, ^2^Sacré-Coeur Hôpital, Montreal, QC, ^3^Centre Hospitalier Universitaire Sainte-Justine, Montreal, QC, ^4^Children’s Hospital at London Health Science Centre, London, ON, ^5^British Columbia Children’s Hospital, Vancouver, BC, ^6^Memorial University, St. John’s, NL, ^7^McMaster University, Hamilton, ON, ^8^University of Toronto, Toronto, ON, ^9^Cumming School of Medicine, Calgary, AB, ^10^Food Allergy Canada, Toronto, ON, ^11^Emergency Medical Services of Outaouais, Outaouais, QC, ^12^Department of Pediatrics, Montreal Children’s Hospital, Montreal, QC, ^13^Division of Allergy and Clinical Immunology, Department of Pediatrics, Montreal Children’s Hospital, Montreal, QC, ^14^Dept Pediatrics and Child Health, University of Manitoba; and Children’s Hospital Research Institute of Manitoba, Winnipeg, MB, ^15^Centre for Outcomes Research and Evaluation, Research Institute of McGill University Health Centre, Montreal, QC, ^16^Department of Pediatrics and Child Health, Section of Allergy and Clinical Immunology, Children’s Hospital Research Institute of Manitoba, Winnipeg, MB, ^17^Department of Pediatrics, University of Alberta, Edmonton, AB, ^18^Division of Allergy and Clinical Immunology, Department of Pediatrics, Montreal Children’s Hospital, McGill University Health Centre, Montreal, QC

*Allergy, Asthma & Clinical Immunology* 2026, **22(2)**: 78

**Background** The 2020 Protection of Students with Life-Threatening Allergies Act, obligates schools across Alberta, Canada to hold a common use stock of at least one epinephrine autoinjector (EAI) and to maintain a protocol for the prevention and management of anaphylaxis Herein, we assessed the effectiveness of this policy by comparing pre-hospital EAI use before and after implementation of the act.

**Methods** The Cross-Canada Anaphylaxis Registry (C-CARE) enrolls patients presenting to a participating emergency department with anaphylaxis defined as the involvement of ≥ 2 systems and/or hypotension. Specific to this project, we extracted data on pediatric patients recruited from the Stollery Children’s Hospital in Edmonton, Alberta from 2016 to 2024. Data were collected on demographics, comorbidities, symptomatology, pre- and intra-hospital management, and outcome of anaphylaxis by standardized chart review. Multivariable logistic regression was used to assess pre-hospital EAI use for cases occurring at school before versus after the enactment of the Act on January 1, 2020. Evaluating pre-hospital EAI use in Quebec and Ontario and antihistamine use pre versus post act in Alberta were used as negative controls.

**Results** Of the 59 cases of in-school anaphylaxis identified over the study period, 52.5% occurred post-Act. The median age was 13.0 years (IQR = 9.5–15.8) and 40.7% were male. EAI use in schools was greater post-act versus pre-act (64.5% versus 35.7%, aOR = 3.82, 95%CI = 1.24–11.7). There was no difference in pre-hospital EAI use pre versus post 2020 in Quebec and Ontario, that did not use the act (aOR = 0.72, 95%CI = 0.31–1.70). There was no difference in pre-hospital antihistamine use pre versus post act (aOR = 0.31, 95%CI = 0.07–1.41).

**Conclusions** Implementation of the provincial policy was associated with an increase in pre-hospital EAI use in cases of pediatric anaphylaxis occurring at school. These preliminary results support the effectiveness of policy interventions for anaphylaxis management.

## 79

### Understanding the atopy-cancer connection: a systematic review and meta-analysis of the relationship of atopy and cancer outcomes

#### Rosenfeld, Aaron^1^, Zhang, Steven X.^1^, Nathan, Paul C.^2^, Upton, Julia E.^3^

##### ^1^Department of Paediatrics, University of Toronto, Toronto, ON, ^2^Division of Haematology/Oncology, University of Toronto, Toronto, ON, ^3^Division of Immunology and Allergy, University of Toronto,, Toronto, ON

*Allergy, Asthma & Clinical Immunology* 2026, **22(2)**: 79

**Background** Prior studies have systematically examined the association between atopy and cancer incidence. However, fewer studies have explored atopy and cancer outcomes, and no systematic reviews exist. Our objective was to systematically evaluate cancer outcomes in patients with atopy.

**Methods** This review was prospectively registered on PROSPERO (CRD420251000280). We searched MEDLINE, EMBASE, and Web of Science (1946 to April 7, 2025). We included observational studies (cohort and case–control) involving children and/or adults that compared cancer outcomes (e.g. survival, progression, metastases) by atopy status. Two independent reviewers screened titles/abstracts and full texts. Risk of bias was assessed with the Newcastle–Ottawa Quality Assessment Scale. Survival data was pooled using random-effects models and expressed as relative risk with 95% CIs. Heterogeneity was assessed using the I^2^ statistic.

**Results** Preliminary results are described below. Of 7,621 studies screened, 4,259 await second reviewer screening. Among 55 full texts reviewed by the first reviewer, 41 met inclusion/exclusion criteria, and 28 cohort studies (n = 7,753,034) had sufficient data (reporting of RR and CIs/p-values) to be included in meta-analysis. The overall relative risk for cancer mortality was not significantly different between atopic and non-atopic individuals (RR 0.94 [0.87–1.02]). Substantial heterogeneity (I^2^ of 95.8%) between studies prompted subgroup analysis. Atopy was found to be protective for prostate cancer (8 studies; RR 0.89 [0.82–0.96]), head and neck, and HPV-related cancers survival (1 study each). Inferior survival was observed for liver, kidney, bladder and connective tissue cancers (1 study each). Having any atopic feature (20 studies; RR 0.90 [0.83–0.97]), allergic rhinitis (25 studies; RR 0.93 [0.90–0.97]) or allergic conjunctivitis (1 study only) was protective for all-cancer pooled mortality. Asthma was associated with worse survival (59 studies; RR 1.15 [1.07–1.23]).

**Conclusions** The relationship between atopy and cancer outcomes appears complex, varying by cancer site and atopy. There is a further need for robust prospective cohort studies.

## 80

### When equal work doesn’t pay: gender-based remuneration gaps among allergists and immunologists in Ontario

#### Tang, Xinxin^1^, Nazarali, Samina^1^, Abdurrahman, Zainab^2, 3, 4^, Kim, Chungah^2^, Mishra, Sujata^2^, Wong, Tiffany^5^, Cook, Victoria E.^5, 6^, Sibley, Lyn M.^2, 7^, Jeimy, Samira^1, 8^

##### ^1^Division of Clinical Immunology and Allergy, Department of Medicine, Western University, London, ON, ^2^Ontario Medical Association, Toronto, ON, ^3^Department of Pediatrics, McMaster University, Hamilton, ON, ^4^School of Medicine, Toronto Metropolitan University, Toronto, ON, ^5^Division of Allergy, Department of Pediatrics, University of British Columbia, Vancouver, BC, ^6^Community Immunology & Allergy Clinic, Island Health Authority, Victoria, BC, ^7^Institute for Health Policy, Management, and Evaluation, University of Toronto, Toronto, ON, ^8^Lawson Health Research Institute, London, ON

*Allergy, Asthma & Clinical Immunology* 2026, **22(2)**: 80

**Background** Despite the growing representation of women in medicine, a persistent gender pay gap remains. In Ontario, women make up over half of Clinical Immunology and Allergy (CIA) physicians, making the field well-suited for examining income disparities given its distinct combination of payment models, high patient volumes, and varied clinical services. This study uses Ministry of Health data obtained by the Ontario Medical Association to quantify the gender pay gap in CIA and identify potential contributing factors.

**Methods** We conducted a cross-sectional analysis to investigate the gender pay gap among physicians in CIA in Ontario, Canada from fiscal year 2018 to 2023. Administrative health data, derived from Ontario Health Insurance Plan billing records, were analyzed to determine differences in clinical payments by gender. To control for potential confounders, multivariable regression models were implemented with outcome variables of log-transformed daily and total payments and explanatory variables including demographic factors, practice characteristics, and work patterns.

**Results** The unadjusted analysis showed male allergists consistently earned significantly higher annual gross payments ($476,694 vs. $290,663, p < 0.0001) and daily gross payments ($2,328 vs. $1,778, p < 0.0001) than their female counterparts. The year-by-year comparisons revealed consistent trends. The results from the regression analysis show that female physicians earned significantly less in total pay compared to their male counterparts, even after adjusting for key confounders. Specifically, females had 17% lower total pay than males (β = -0.17, 95% CI: -0.27, -0.08, p < 0.001).

**Conclusions** This is the first study to quantify the gender pay gap in CIA using population-level billing data in Ontario. Our analysis reveals that women physicians earn significantly less than their male counterparts, even after adjusting for demographic variables, practice characteristics, and work patterns. These findings point to systemic inequities and emphasize the need to address gender-based disparities to support physician retention, satisfaction, and quality of care.

## 81

### Allergyiq: an interactive virtual educational platform in allergy and immunology

#### Aw, Michael J.^1^, AlYaqout, Fatemah^2^, Feteih, Abeer^3^, Tardio, Natacha^2^, Fein, Michael^2^

##### ^1^Faculty of Medicine, Department of Internal Medicine, McGill University, Montreal, QC, ^2^Division of Clinical Immunology and Allergy, Department of Medicine, McGill University Health Center, Montreal, QC, ^3^Department of Medicine, Faculty of Medicine in Rabigh, King Abdulaziz University, Jeddah, Saudi Arabia

*Allergy, Asthma & Clinical Immunology* 2026, **22(2)**: 80.

**Background** Allergy and Clinical Immunology (A/I) remains underrepresented in Canadian medical education despite increasing prevalence of allergic diseases. A/I training opportunities are limited, non-standardized, and vary institutionally. Resident surveys at McGill University Health Centre (MUHC) (n = 68) have demonstrated low confidence in A/I competencies, particularly for immune deficiencies (confidence < 6.1% across domains versus 11.8–29.4% for allergic conditions).

We propose *AllergyIQ,* a pilot, innovative self-guided virtual course designed to provide comprehensive, accessible learning experiences.

**Methods** *AllergyIQ* addresses key A/I concepts aligned with national Internal Medicine training standards. *AllergyIQ* features a modular design, enabling self-paced learning. The course incorporates a range of pedagogical methods such as case-based learning, interactive infographics, and multimedia content. By blending these modern learning tools with traditional educational principles, the platform maximizes learner engagement and retention.

In this pilot project, study participants include medical trainees completing an A/I elective in Adult A/I at MUHC from March 2025–2026. Learners will complete up to 4 online modules (e.g. anaphylaxis, drug/food allergy, urticaria/angioedema) to supplement their clinical learning experience. Participant satisfaction and knowledge acquisition is measured with pre-post program competency, satisfaction, and knowledge questionnaires. Data will be analyzed using Wilcoxon signed-rank test.

**Results** Given the increasing prevalence of allergic diseases and regional shortages of allergists and allergy educators, the need for accessible A/I education is increasingly important. Moreover, poor understanding and limited exposure may negatively influence career planning in an already underserviced subspecialty.

*AllergyIQ* implements evidence-based learning pedagogical frameworks to optimize the learner experience. It is a free, online learning resource that can be accessed nationwide, therefore extending the reach of high-quality educational resources to all health care professionals. Piloting of this initiative is actively underway with elective trainees at the MUHC and initial feedback has been positive.

**Conclusions** Long-term, *AllergyIQ* aims to improve the A/I education for medical trainees, ultimately leading to improved patient care.

## 82

### Occupational skin symptoms from cleaning products in healthcare workers

#### Chan, Felix^1^, Holness, Linn^2, 3, 4^, Tarlo, Susan M.^2, 3, 5^

##### ^1^Division of Clinical Immunology and Allergy, Department of Medicine, McMaster University, Hamilton, ON, ^2^Department of Medicine, University of Toronto, Toronto, ON, ^3^Dalla Lana School of Public Health, University of Toronto, Toronto, ON, ^4^MAP Centre for Urban Health Solutions, St Michael’s Hospital, Unity Health Toronto, Toronto, ON, ^5^Department of Medicine, University Health Network, Toronto, ON

*Allergy, Asthma & Clinical Immunology* 2026, **22(2)**: 82

**Background** Healthcare workers are at an elevated risk of developing occupational contact dermatitis, and this may relate to frequent exposure to cleaning products. The purpose of our study was to better understand exposures to cleaning products in healthcare workers and if workers in particular roles and settings are more susceptible to adverse skin conditions.

**Methods** Healthcare workers and administrative controls were recruited from the University Health Network, Toronto, and administered a survey capturing information on demographics, occupational exposures, skin symptoms, and effects on quality of life and employment between 2019 to 2023.

**Results** We recruited 333 participants (99 nurses, 79 cleaners, 39 medical device reprocessing technicians, 60 other staff, and 53 administrative workers). The mean age was 43 years. Females represented 77% of participants (94% of nurses and 44% of MDR technicians; p < 0.001). The most common cleaning product exposures were isopropanol (77%), hypochlorite bleach (59%), quaternary ammonium compounds (45%), and hydrogen peroxide (41%). 26% of overall participants reported a work-related hand rash in the past 12 months (35% of nurses and 17% of administrative workers). Factors associated with a work-related hand rash in the preceding year included: direct patient care, frequent glove use, frequent handwashing, frequent hand sanitizer use, instrument cleaning, and exposure to accidental spills. 65% and 32% of participants identified their current hand rash severity as normal and mild, respectively. The most impacted quality of life measure was occupation (26% and 9% reporting slight and moderate effects).

**Conclusions** We report a high prevalence of skin rashes and symptoms among healthcare workers, particularly in nurses. Our study reaffirms known risk factors for occupational contact dermatitis, including exposure to wet work, cleaning agents, and frequent hand cleansing. Most participants identified their current hand rash severity as mild, and there were slight effects on quality of life associated with skin symptoms.

## 83

### AI-assisted versus text-based patient-led penicillin allergy risk assessment: a randomized trial assessing patient motivation

#### Horwitz, Simonne L.^2^, Jacob, John^3, 2^, Wong, Tiffany^1, 2^

##### ^1^Division of Allergy, University of British Columbia, Vancouver, BC, ^2^Department of Pediatrics, University of British Columbia, Vancouver, BC, ^3^Digital Lab, Vancouver, BC

*Allergy, Asthma & Clinical Immunology* 2026, **22(2)**: 83

**Background** We previously developed a validated patient-led penicillin allergy risk assessment tool at the BC Children’s and Women’s Hospital to educate patients.^1^ The primary outcome of this project is to determine whether patient motivation to have their allergy label assessed differs after use of the text-based tool versus an AI physician-assisted video adaptation of the tool.

**Methods** In this prospective trial, patients over six months old with a penicillin allergy were invited to the study and randomized into either a “text” or “video” tool (both available in English, Mandarin, and Punjabi). The text group read questions related to suspected allergy while the video group had questions asked by an AI-generated physician. Participants responded to three text statements on a scale from 1 (strongly disagree) to 7 (strongly agree) before and after using the tool: (1) “I expect that I have a penicillin allergy”, (2) “I want to be tested for my penicillin allergy”, and (3) “I intend to make an appointment to be tested for my penicillin allergy”. Responses were compared and numerical and proportional data presented.

**Results** 30 patients have completed the intervention, with 17 randomized to the video group and 13 to the text group. 11/17 (65%) participants in the video group changed their expectation of having a penicillin allergy after using the AI-assisted tool, compared to 6/13 (46%) of those in the text group. In contrast, only 7/17 (41%) participants in the video group changed their desire for penicillin allergy testing, compared to 9/13 (69%) of participants in the text group.

**Conclusions** Interim results suggest that there is no clear difference in patient motivation when using a video versus text tool. Providing patients with the option to choose different languages and versions of the tool will allow for increased autonomy and flexibility depending on personal preference and comfort.

**References**

1. Penicillin allergy de-labeling: Adaptation of risk stratification tool for patients and families. Horwitz, Simonne L. et al. World Allergy Organization Journal, Volume 17, Issue 8, 100939.

## 84

### Characterizing allergy-related misinformation on social media: a cross-platform analysis of themes, engagement, and public response

#### Khadija, Udain^1^, Vohra-Miller, Sabina^3^, Jeimy, Samira^2^

##### ^1^Schulich School of Medicine and Dentistry, Western University, London, ON, ^2^Division of Clinical Immunology & Allergy, Department of Medicine, Western University, London, ON, ^3^Dana Lana School of Public Health, University of Toronto, Toronto, ON

*Allergy, Asthma & Clinical Immunology* 2026, **22(2)**: 84

**Background** Allergic diseases affect approximately one in five Canadians and are among the most common chronic health conditions in both children and adults [1]. Despite their prevalence, allergy misinformation remains underexplored in the digital landscape. Social media platforms are increasingly used for health guidance in Canada, but they are also major sources of misinformation [2]. This study aimed to characterize the spread and engagement of allergy-related misinformation across major platforms and assess public sentiment.

**Methods** We conducted a qualitative content analysis of allergy-related misinformation posts across Twitter/X, TikTok, Instagram, and Facebook from March–May 2025. Posts were identified using systematic keyword searches and manual navigation of wellness influencer accounts. Inclusion criteria required publicly accessible, English-language content promoting claims contradicting established allergy guidelines from AAAAI, CSACI, or EAACI. A structured codebook categorized seven primary misinformation themes. Two independent reviewers coded posts with high inter-rater reliability (Cohen’s Kappa = 0.84). Engagement metrics and comment sentiment were analyzed using a three-point scale.

**Results** We analyzed 347 unique misinformation posts distributed evenly across platforms. The most prevalent themes were natural cures (41%), IgG food sensitivity testing promotion (21%), and medication fear narratives (19%). Natural remedy posts achieved highest engagement (median 13,400 likes, 1,250 shares) compared to fear-based content. Sentiment analysis of 50 high-engagement posts revealed 62% supportive comments, 24% neutral responses, and only 14% corrective or skeptical comments. TikTok dominated natural cure content, while Twitter showed higher proportions of IgG testing and medication fear posts.

**Conclusions** Allergy misinformation circulates widely on social media with minimal correction, potentially delaying evidence-based care. Proactive digital communication strategies by clinicians and medical societies are urgently needed to combat misleading narratives and protect public health.

**References**

1. Government of Canada. Common chronic diseases in Canada. Ottawa: Public Health Agency of Canada; 2020. Available from: https://www.canada.ca/en/public-health/services/publications/diseases-conditions/common-chronic-diseases.html
2. Pew Research Center. Social media use continues to rise in Canada. Washington: Pew Research Center; 2023. Available from: https://www.pewresearch.org/internet/2023/04/12/social-media-use-in-2023/
3. Sicherer SH, Sampson HA. Food allergy: A review and update on epidemiology, pathogenesis, diagnosis, prevention, and management. J Allergy Clin Immunol. 2018;141(1):41-58.
4. Canadian Society of Allergy and Clinical Immunology. CSACI Position Statement on IgG Testing. Ottawa: CSACI; 2022. Available from: https://csaci.ca/position-statements/igg-testing/
5. Vosoughi S, Roy D, Aral S. The spread of true and false news online. Science. 2018;359(6380):1146–1151.

## 85

### Characterizing drug allergy management among allergists in Canada: a national survey study

#### Lee, Brian^1^, Lee, Erika^2, 3^, Krishnan, Sinthiha^5^, Jeimy, Samira^1, 4^, Picard, Matthieu^6^, Rosenfield, Lana^7^, Ruiz, Juan^8^, Song, Christine^2, 3, 9^

##### ^1^Division of Clinical Immunology & Allergy, Department of Medicine, Western University, London, ON, ^2^Division of Allergy & Immunology, Department of Medicine, University of Toronto, Toronto, ON, ^3^Drug Allergy Clinic, Sunnybrook Health Sciences Centre, Toronto, ON, ^4^Lawson Research Institute, St. Joseph’s Health Care London, London, ON, ^5^. Faculty of Medicine, University of Toronto, Toronto, ON, ^6^Division of Allergy and Clinical Immunology, Hôpital Maisonneuve-Rosemont; Department of Medicine, Université de Montréal; Centre de Recherche de l’Hôpital Maisonneuve-Rosemont, Montreal, QC, ^7^Section of Allergy and Clinical Immunology, University of Manitoba, Winnipeg, MB, ^8^Division of Allergy & Immunology, University of British Columbia, Vancouver, BC, ^9^Division of Allergy & Immunology, St. Michael’s Hospital, Toronto, ON

*Allergy, Asthma & Clinical Immunology* 2026, **22(2)**: 85

**Background** Mislabelling of drug allergy is associated with potential harms to the patient and costs to the healthcare system, and affects 10–15% of Canadians. Clinical infrastructure to assess and verify these labels are variable and understudied in the Canadian context.

**Methods** We developed a 40-item survey which was distributed to members of the Canadian Society of Allergy and Clinical Immunology from January 2024 to August 2024 with the goal of assessing practice patterns, testing modalities, and barriers to drug allergy testing perceived by allergists.

**Results** From a total of 66 respondents, most were based in Ontario (40.9%, n = 27) and Quebec (39.4%, n = 26). 48.4% (n = 31) practised solely in the community setting compared to 21.9% (n = 14) in the academic setting, with 29.7% (n = 19) in a mixed setting. Referrals for drug allergy assessment were common, with 96.9% (n = 62) seeing at least one patient per week for drug allergy. Most respondents (87.9%, n = 51) performed drug allergy testing in their clinic though advanced diagnostic procedures such as intradermal testing (81.1% vs 48.7%, p = 0.004), patch testing (38.2% vs 8.8%, p = 0.009), and non-oral drug challenges (63.6% vs 20.0%, p = 0.0005) were more commonly performed by hospital-based allergists compared to community-based allergists. Perceived barriers to drug allergy testing included lack of support personnel, access to emergency infrastructure, financial costs, and lack of access to the culprit drugs.

**Conclusions** This survey demonstrates significant variation in the practice of drug allergy management among Canadian allergists driven largely by practice setting, with implications for future advocacy regarding resource allocation to ensure equitable access to drug allergy care across Canada.

## 86

### Stability of skin-testing concentrations of penicillin G 10,000 units/mL in glass vials under refrigeration and at room temperature for 15 days

#### Lo, Stephanie^1^, Law, Shirley^1^, Perks, William^1^, Moolani Merchant, Yasmin^2, 3^, Lee, Erika^2, 3^, Fu, Lisa^2, 3^, Song, Christine^2, 3^, Ramjohn, Linda^3^, Campbell, Jackie^1^, Ma, Nathan H^1^

##### ^1^Department of Pharmacy, Sunnybrook Health Sciences Centre, Ontario, ON, ^2^. Department of Medicine, University of Toronto, Toronto, ON, ^3^Integrated Community Program, Sunnybrook Health Sciences Centre, Toronto, ON

*Allergy, Asthma & Clinical Immunology* 2026, **22(2)**: 86

**Background** Penicillin skin testing is an important clinical tool to risk stratify suspected penicillin allergy before drug provocation testing, but there are no validated stability data for dilute penicillin G at skin testing concentrations.

**Methods** Our objective is to evaluate the chemical stability of penicillin G 10,000 units/mL in 0.9% sodium chloride (NS) stored in glass vials at room temperature (25 °C) and refrigerated (4 °C) for 15 days.

On study day 0, six penicillin G vials were reconstituted then diluted with NS to 10,000 units/mL and stored in glass vials. Three vials of each were stored under refrigeration and at room temperature. Concentrations were determined on study days 0,1,2,3,7,9,11,15 using a validated, stability-indicating liquid chromatographic method with UV detection. The chemical stability was determined by calculating the intersection of the lower limit of the 95% confidence interval of the observed degradation rate and the time to achieve 90% of the initial concentration.

**Results** The analytical method separated penicillin G from its degradation products such that the concentration of penicillin G was measured specifically, accurately (deviations from known averaged 0.79%), and reproducibly (within day variation averaged 0.33% and between day variation averaged 1.12%).

Penicillin G stored at 4 °C retained 41.06% of its initial concentration at 15 days and when stored at room temperature, it degraded to 43.71% of its initial concentration within 1 day. The calculated time to achieve 90% of the initial concentration with 95% confidence was 3 days when stored in the refrigerator and less than 1 day when stored at room temperature.

**Conclusions** Penicillin G 10,000 units/mL in NS stored in glass vials is stable for 3 days under refrigeration and for less than 1 day at room temperature. This finding suggests that the same diluted vial can be used for up to 3 days, thus saving time, money and resources.

## 87

### Familial aquagenic pruritus without urticaria: a case series suggesting a heritable pruritic disorder

#### Luo, Candice, Salimi, Payam, McKibbin, Lundy

##### University of Manitoba, Winnipeg, MB

*Allergy, Asthma & Clinical Immunology* 2026, **22(2)**: 87

**Background** Aquagenic pruritus (AP) is a rare condition characterized by intense itching upon contact with water, typically without visible skin changes. While frequently associated with myeloproliferative neoplasms, idiopathic forms remain poorly understood, and no familial cases have been documented to date. This case series describes the first reported familial cluster of idiopathic aquagenic pruritus, suggesting a potential heritable component to the condition.

**Methods** Three biologically related individuals from two generations of a non-consanguineous Caucasian family were evaluated at our Adult Allergy and Immunology Clinic between March 2024 and February 2025 in Winnipeg, Manitoba. Clinical histories, laboratory data, and standardized provocation tests (wet towel, ice cube, and dermatographism) were used to characterize symptoms and rule out other inducible urticarias.

**Results** Case 1 (59-year-old female) had severe, lifelong symptoms with partial response to desloratadine and skin barriers. Laboratory workup was notable for a low JAK2 V617F allele burden without clinical MPN. Case 2 (62-year-old male) reported adolescent-onset pruritus that improved over time; his workup was unremarkable. Case 3 (25-year-old female) presented with early-onset, persistent symptoms and moderate life quality impact. All three patients experienced water-induced pruritus without urticaria or rash, with positive responses to the wet towel test. All had negative ice cube and dermatographism tests.

**Conclusions** This case series represents the first formally documented familial cluster of aquagenic pruritus, suggesting a potential genetic predisposition. These findings highlight the need for further studies to elucidate the underlying mechanisms of idiopathic AP and explore possible hereditary patterns.

*All three patients gave their informed and explicit consent to publish their data in an online, open-access journal.*

## 88

### Second-line fluoroquinolone prescriptions in patients with beta-lactam allergies and the resultant implementation of a penicillin reaction clinic in a community hospital

#### Morris, Emily G.^1^, Setia, Abhishiek^2, 3^, Haffner, Thomas^2, 3^, Huan, Peter W.^1^, Sadyora, Jasraj^4^, Narayan, Yasmin^5^, Kim, Harold^6, 7^

##### ^1^Western University, Department of Medicine, London, ON, ^2^Huron Perth Healthcare Alliance, Stratford, ON, ^3^Western University, Schulich School of Medicine & Dentistry, London, ON, ^4^Michigan State University College of Human Medicine, Grand Rapids, MI, USA, ^5^Queen’s University, Faculty of Arts and Science, Kingston, ON, ^6^Division Clinical Immunology and Allergy, Department of Medicine, Western University, London, ON, ^7^Faculty of Health Sciences, McMaster University, Hamilton, ON

*Allergy, Asthma & Clinical Immunology* 2026, **22(2)**: 88

**Background** Patients labeled with penicillin allergies often receive broad spectrum and second-line antibiotics, despite a history of non severe symptoms. This two-phase study examines prescriptions of second-line fluoroquinolones in patients labeled with beta-lactam allergies, and the impact of a community Penicillin Reaction Clinic in the Huron Perth Healthcare Alliance (HPHA) that followed phase one findings.

**Methods** Phase one involved a retrospective chart review of 339 patients prescribed fluoroquinolones between May 2017 and May 2018 from four community hospitals in the HPHA. The review assessed whether patients had beta-lactam allergies and if beta-lactams were the first-line treatment for their diagnosis. Based on phase one findings, the HPHA launched a community Penicillin Reaction Clinic run by non-allergist physicians. The clinic administered oral amoxicillin challenges to patients with non-severe penicillin allergies. Phase two involved a retrospective chart review of 529 patients challenged at the clinic from 2019 to 2024, examining reactions versus de-labeling of the allergy.

**Results** Phase one found that of the 339 patients prescribed fluoroquinolones, 30% (103/339) had a reported beta-lactam allergy. The majority (81.6%) had non-severe or unknown symptoms, and 70.2% were being treated for diagnoses where beta-lactams were first-line. In phase two, 529 patients were challenged at the clinic; 493 (93.2%) were deemed non-allergic. Two (0.4%) were too high risk to proceed. Thirty-four (6.4%) patents reacted to the challenge. Most reactions were non severe, with 32 (94.1%) either grade 1 or 2 per World Allergy Association 2024 grading system for systemic allergic reactions.

**Conclusions** Many patients received second-line antibiotics when beta-lactams were recommended first-line due to reported penicillin allergy. By implementing a community allergy testing clinic for low risk patients, run by non-allergist physicians, the HPHA was able to reduce inaccurate penicillin allergy labeling. This clinic provides a framework for other community sites.

## 89

### Optimizing use of cefazolin surgical prophylaxis in patients with beta-lactam allergy: preliminary report on a quality improvement initiative

#### Nazarali, Samina^1^, Aasen, Amy^2^, Bondy, Lise^3^, Jeimy, Samira^1^

##### ^1^Division of Clinical Immunology and Allergy, Department of Medicine, Western University, London, ON, ^2^Pharmacy Services, St Joseph’s Health Care London, London, ON, ^3^Division of Infectious Diseases, Department of Medicine, Western University, London, ON

*Allergy, Asthma & Clinical Immunology* 2026, **22(2)**: 89

**Background** Approximately 10% of the Canadian population reports a beta-lactam allergy, though over 90% are mislabeled. Perioperatively, this often leads to second-line antibiotic use, increasing the risk of surgical site infections, prolonged hospital stays, and ICU admissions. The objective of this quality improvement (QI) initiative is to increase the evidence-based use of cefazolin prophylaxis in patients with reported beta-lactam allergy.

**Methods** An adapted version of the Allergy Clarification for Cefazolin Evidence-based Prescribing Tool (ACCEPT) [1] was recently approved for implementation at St. Joseph’s Health Care London, Ontario. This decision-support tool uses three screening questions to assess if a patient can safely receive cefazolin. Implementation will follow a QI framework with ongoing monitoring and iterative adaptation through Plan-Do-Study-Act cycles. A chart review of cases from a 14-day period in November 2024, was conducted to evaluate baseline cefazolin use in patients with listed beta-lactam allergies prior to implementation of the tool.

**Results** 332 surgical cases were reviewed, and 12% (n = 41) of patients had a documented beta-lactam allergy. Among that subgroup, 22% received prophylaxis not in line with current recommendations. Unnecessary avoidance of cefazolin based on documented allergy history was a main theme in this group. In 10% of cases, the rationale for selecting a non-cefazolin regimen was unclear.

**Conclusions** Chart review findings highlight the opportunity to increase cefazolin prophylaxis usage in patients with reported beta-lactam allergies. Implementation of the decision support tool through targeted education sessions, stakeholder engagement, and user feedback, will be essential in enhancing uptake. These efforts aim to improve surgical care in London, Ontario, with potential for broader application to other centres across the country.

**References**

1. Lam PW, Tarighi P, Elligsen M, Nathens AB, Riegert D, Tarshis J, et al. Impact of the allergy clarification for cefazolin evidence-based prescribing tool on receipt of preferred perioperative prophylaxis: an interrupted time series study. Clin Infect Dis. 2020 Dec 1;71(11):2955–7.

## 90

### Dupilumab-associated symptomatic hypereosinophilia: a systematic review

#### Rayner, Daniel G.^1^, Sun, Wayne^1^, Ologundudu, Leonardo^2^, Fernandes, Athena^3^, Kim, Harold L.^1, 4, 5^

##### ^1^Schulich School of Medicine and Dentistry, Western University, London, ON, ^2^Michael G. DeGroote School of Medicine, McMaster University, Hamilton, ON, ^3^Faculty of Health Sciences, McMaster University, Hamilton, ON, ^4^Division of Clinical Immunology and Allergy, Department of Medicine, Western University, London, ON, ^5^Department of Medicine, McMaster University, Hamilton, ON

*Allergy, Asthma & Clinical Immunology* 2026, **22(2)**: 90

**Background** Dupilumab, an IL-4/11 inhibitor, is a biologic agent approved for the treatment of moderate-to-severe atopic dermatitis, severe asthma, and chronic rhinosinusitis with nasal polyps (CRSwNP). In rare cases, patients taking dupilumab can experience symptomatic hypereosinophilia (absolute blood eosinophil count ≥ 1500 cells/μL), however, their clinical presentations are poorly understood. We conducted a systematic review to summarize the presentation, management, and prognosis of symptomatic hypereosinophilia following dupilumab administration.

**Methods** We searched MEDLINE, Embase, and CENTRAL from database inception to May 22nd, 2025, for studies reporting on the management of one or more patients with hypereosinophilia following dupilumab administration. We excluded studies reporting on patients with baseline hypereosinophilia prior to the initiation of dupilumab. Paired reviewers independently screened citations and extracted data.

**Results** We included 39 studies reporting on 80 patients with symptomatic hypereosinophilia following dupilumab administration. Twenty studies (51%) were case reports, 9 (23%) were case series, 8 (21%) were cohort studies, and 2 (5%) were randomized trials. The included patients had a mean age of 54.7 years and 49/74 (66%) were female. No study reported on symptomatic hypereosinophilia post-dupilumab administration in pediatric patients (≤ 18 years). The most common type 2 inflammatory conditions were asthma (67/78 [87%]), CRSwNP (45/75 [60%]), and allergic rhinitis (9/72 [13%]). The most frequently reported adverse events included eosinophilic granulomatosis with polyangiitis (21/80 [26%]), eosinophilic pneumonia (21/80 [26%]), asthma worsening (14/80 [18%]), and arthralgia or myalgia (10/80 [13%]). Commonly reported management approaches included the discontinuation of dupilumab, starting systemic steroids, and beginning biologic therapies targeting eosinophils, including mepolizumab, rituximab, and benralizumab. No deaths were reported across the included studies.

**Conclusions** Dupilumab-induced symptomatic hypereosinophilia remains a significant clinical challenge for clinicians managing patients on dupilumab. Further research is needed to determine patient-related risk factors for dupilumab-associated hypereosinophilia, and to develop evidence-based recommendations for its management.

## 91

### An 18-month review of chemotherapy and biologic desensitization outcomes in canada’s first oncology-allergy clinic

#### Salimi, Payam^1^, Geirnaert, Marc^2^, Zaborniak, Karver^3^

##### ^1^Department of Internal Medicine, University of Manitoba, Winnipeg, MB, ^2^Provincial Oncology Drug Program, CancerCare Manitoba, Winnipeg, MB, ^3^Section of Clinical Immunology and Allergy, Department of Internal Medicine, University of Manitoba, Winnipeg, MB

*Allergy, Asthma & Clinical Immunology* 2026, **22(2)**: 91

**Background** In September 2022, Canada’s first dedicated oncology-allergy clinic was established at CancerCare Manitoba to support patients with hypersensitivity reactions to chemotherapy and biologic agents. This specialized clinic provides allergy assessment and desensitization recommendations for individuals requiring continued access to indicated therapies. In collaboration with the provincial oncology drug program, standardized outpatient desensitization protocols were implemented to facilitate the safe re-challenge of therapeutic agents. This initiative enables patients to continue essential treatments while minimizing delays and interruptions in cancer care.

**Methods** A retrospective chart review was performed for all patients who underwent chemotherapy or biologic desensitization between October 1, 2022, and March 31, 2024. Demographic data, hypersensitivity reactions, agents, desensitization protocols, and clinical outcomes were collected. Agents were grouped by type for analysis. All desensitization protocols were standardized and available through CancerCare Manitoba’s Regimen Reference Order system, a publicly accessible resource developed with multidisciplinary input. Protocols were individualized based on patient history and allergy assessment, including skin testing where applicable.

**Results** Preliminary analysis identified 64 patients who underwent desensitization during the study period, accounting for 440 desensitization doses. The most frequently managed agents were paclitaxel (111 doses), rituximab (97), nab-paclitaxel (56), and trastuzumab (51). Desensitization success, defined as completion of the planned infusion, was achieved in the vast majority of doses. Mild breakthrough reactions occurred during a small proportion of doses, while the need for epinephrine administration or emergency department transfer was rare.

**Conclusions** Early experience from Canada’s first oncology-allergy clinic demonstrates that chemotherapy and biologic desensitization can be safely delivered using standardized outpatient protocols. The majority of breakthrough reactions were mild and manageable, enabling patients to continue first-line cancer therapies. These findings underscore the importance of oncology-allergy collaboration and structured desensitization pathways in maintaining treatment access with their prescribed treatment protocol.

## 92

### Dupilumab improves outcomes in children with eosinophilic esophagitis weighing ≥ 15 kg independent of individual atopic comorbidity history

#### Cianferoni, Antonella^1^, Chehade, Mirna^2^, Gold, Benjamin D.^3^, Aceves, Seema^4^, Vozza, Nicolas^5^, Gonzalez, Carlos^6^, Zaghloul, Sherif^7^, Raphael, Bram P.^6^, Angello, James T.^7^, Radwan, Amr^6^

##### ^1^Division of Allergy and Immunology, Department of Pediatrics, The Children’s Hospital of Philadelphia, University of Pennsylvania School of Medicine, Philadelphia, PA, USA, ^2^Mount Sinai Center for Eosinophilic Disorders, Icahn School of Medicine at Mount Sinai, New York, NY, USA, ^3^Children’s Center for Digestive Healthcare, LLC, Children’s Healthcare of Atlanta, Atlanta, GA, USA, ^4^Division of Allergy Immunology, Departments of Pediatrics and Medicine, University of California and Rady Children’s Hospital, San Diego, CA, USA, ^5^Sanofi, Calgary, AB, ^6^Regeneron Pharmaceuticals Inc., Tarrytown, NY, USA, ^7^Sanofi, Morristown, NJ, USA

*Allergy, Asthma & Clinical Immunology* 2026, **22(2)**: 92

**Background** Eosinophilic esophagitis (EoE) is a chronic type 2 inflammatory disease of the esophagus. Many patients with EoE are affected by atopic comorbidities due to shared etiologic mechanisms. Dupilumab is approved for the treatment of EoE in patients aged ≥ 1 year, weighing ≥ 15 kg. This analysis assessed the efficacy of weight-tiered higher-exposure dupilumab vs placebo in children aged 1–11 years weighing ≥ 15 kg with EoE from the phase 3 EoE KIDS trial (NCT04394351), according to baseline history of individual atopic comorbidities: atopic dermatitis (AD), asthma, allergic rhinitis (AR), and food allergy (FA).

**Methods** Endpoints (Week 16) included proportions of patients achieving peak esophageal intraepithelial eosinophil count (PEC) ≤ 6 and < 15 eosinophils per high-power field (eos/hpf); and mean change in Endoscopic Reference Score (EREFS) and Histology Scoring System (HSS) grade and stage scores.

**Results** At baseline, 84/87 (96.6%) patients in the ≥ 15 kg subgroup had ≥ 1 atopic comorbidity. At Week 16, dupilumab treatment led to greater proportions of patients achieving PEC ≤ 6 eos/hpf regardless of individual atopic comorbidities when analyzed separately (rate difference vs placebo [95% CI]: AD: 73.7% [53.9–93.5], no AD 45.5% [14.2–76.8]; asthma: 53.7% [29.7–77.8], no asthma 77.8% [50.6–100.0]; AR: 60.1% [39.3–81.0], no AR 66.7% [29.0–100.0]; FA: 61.0% [40.9–81.1], no FA 66.7% [29.0–100.0]). Dupilumab treatment also led to greater proportions of patients achieving PEC < 15 eos/hpf regardless of individual atopic comorbidities (rate difference vs placebo [95% CI]: AD: 94.7% [84.7–100.0], no AD 53.2% [22.5–83.9]; asthma: 66.8% [44.3–89.2], no asthma 100.0% [100.0–100.0]; AR: 75.5% [57.3–93.7], no AR 83.3% [53.5–100.0]; FA: 76.4% [59.1–93.7], no FA 83.3% [53.5–100.0]). EREFS and HSS grade and stage scores were also improved vs placebo in all subgroups.

**Conclusions** Dupilumab improved features of EoE vs placebo in children aged 1–11 years weighing ≥ 15 kg, regardless of baseline history of individual atopic comorbidities when analyzed separately.

## 93

### Outcomes of single-day challenge to macrolide antibiotics in adults

#### Wang, Chloe^1^, Campbell, Jackie^2^, Fu, Lisa^2^, Moolani-Merchant, Yasmin^2^, Song, Christine^2^, Lee, Erika^2^

##### ^1^Western University, Toronto, ON, ^2^Sunnybrook Health Sciences Centre, Toronto, ON

*Allergy, Asthma & Clinical Immunology* 2026, **22(2)**: 93.

**Background** Up to 10% of people carry an antibiotic allergy label, and unverified labels negatively impact patient care and health outcomes. It is therefore essential to improve the diagnostic accuracy of allergy testing, which typically involves skin tests and/or drug challenges. Evidence supports direct penicillin challenges in low-risk patients without prior skin testing. However, there is limited data to support similar results for macrolides, underscoring the need for a new adult study on direct macrolide challenges.

**Methods** We conducted a retrospective chart review (January 2022 to December 2024) of adults assessed for suspected macrolide allergy at a drug allergy clinic. Data was stored on REDCap and descriptive statistics were used to characterize demographics, index reactions, and testing outcomes. The study received institutional REB approval.

**Results** A total of 189 unique patient visits were included (mean age = 52 [39, 64]; 76.7% female). Baseline health was assessed using the Charlson Comorbidity Index (mean score = 1 [0, 3]). Index reactions were immediate (n = 33, 17.5%), delayed (n = 88, 46.6%), or unknown (n = 68, 36.0%), with 2 SCARs. Skin testing was performed in 3 patients (1.6%), all negative. Oral challenges (n = 188, 99.5%) were performed on the culprit macrolide (n = 125, 66.1%) or an alternative (n = 63, 33.3%), and the type of macrolide included azithromycin (n = 111, 58.7%), clarithromycin (n = 56, 29.6%), and erythromycin (n = 21, 11.1%; discontinued in Canada after November 2023). Most patients passed (n = 180, 95.2%), with few having positive immediate (n = 4, 2.1%), delayed (n = 2, 1.1%), or inconclusive (n = 2, 1.1%) reactions. Of the positive reactions, 2 were to the culprit macrolide, and 4 to an alternative.

**Conclusions** In a low-risk patient population, skin testing for macrolides is unsupported, and direct oral challenges are generally safe and effective in diagnosing and removing labels. This study provides up-to-date information on macrolide allergies in adults and expands the literature surrounding macrolide testing.

## 94

### Efficacy of therapies in acute hereditary angioedema attacks: a systematic review and meta analysis

#### Chung, Jason^1^, Yu, Andrew^2^, Chung, Zinnia^3^, Lee, Sangmin^4^, Zhu, Rongbo^1^

##### ^1^Division of Clinical Immunology and Allergy, Department of Medicine, Western University, London, ON, ^2^University of Alberta Faculty of Medicine & Dentistry, Edmonton, AB, ^3^Michael G. DeGroote School of Medicine, McMaster University, Hamilton, ON, ^4^Department of Medicine, Western University, London, ON

*Allergy, Asthma & Clinical Immunology* 2026, **22(2)**: 94

**Background** Hereditary angioedema (HAE) is a rare but potentially life-threatening condition characterized by acute and unpredictable cutaneous or submucosal swelling. Untreated, an episode of acute HAE is associated with significant risk of morbidity and mortality. Over the past two decades, there has been an emergence of therapies approved for the acute treatment of HAE attacks. The objective of this review is to determine the efficacy of various HAE therapies, as determined by time to onset of symptom relief and time to complete resolution of all symptoms.

**Methods** A systematic literature search was conducted on five databases from 1974–2025 for English publication. Randomized and non-randomized studies of patients with HAE receiving acute treatment therapies (Recombinant C1 Esterase Inhibitor (R-C1-INH, Plasma Derived C1 Inhibitor (P-C1-INH), Icatibant, Ecallantide, Sebetralstat) were included.

**Results** Seventy-three studies were eligible for meta-analysis. The mean time to onset of symptom relief was 422 min (95%CI: 282,563) for the placebo group, 82 min (95%CI: 65,99) for the Icatibant group, 77 min (95%CI: 63,91) for the P-C1-INH group, 70 min (95%CI: 60,80) for the R-C1-INH group and 125 min (95%CI: 98,151) for the Sebetralstat group. The mean time to complete resolution of all symptoms was 2195 min (95%CI: 1458,2933) for the placebo group, 586 min (95%CI: 434,764) for the Icatibant group, 1218 min (95%CI: 1015,1420) for the P-C1-INH group, 407 min (95%CI: 301,512) for the R-C1-INH group and 428 min (95%CI: 386,470) for the Sebetralstat group.

**Conclusions** In this review, we demonstrate the efficacy of various therapies for acute HAE attacks. Considerations before choosing a therapy may include route of administration, availability, side effect profile and cost. Further research, such as head-to-head comparisons of therapies are required to develop evidence-based guidelines for choosing between treatments and prescribing personalized treatment plans for patients living with HAE.

## 95

### Long-term safety, efficacy, and quality of life (QoL) of navenibart in hereditary angioedema (HAE): Initial results from ALPHA-SOLAR

#### Adatia, Adil^1^, Yang, William H.^2^, Riedl, Marc A.^3^, Craig, Timothy J.^4^, Jacobs, Joshua S.^5^, Tachdjian, Raffi^6^, Lumry, William R.^7^, Sitz, Karl V.^8^, Li, H. Henry^9^, Soong, Weily^10^, Manning, Michael E.^11^, Wedner, James^12^, Morabito, Christopher^13^, Cohen, Theodora^13^, Bernard, Kristine^13^, VanEenwyk, Claire^13^, Best, Jessica^13^, Banerji, Aleena^14^

##### ^1^University of Alberta, Edmonton, AB, ^2^Ottawa Allergy Research Corporation, Ottawa, ON, ^3^University of California-San Diego, San Diego, CA, USA, ^4^Penn State Health Allergy, Asthma and Immunology, Hershey, PA, USA, ^5^Allergy and Asthma Clinical Research, Inc., Walnut Creek, CA, USA, ^6^UCLA School of Medicine, Los Angeles, Los Angeles, CA, USA, ^7^AARA Research Center, Dallas, TX, USA, ^8^Little Rock Allergy and Asthma Clinic, Little Rock, AR, USA, ^9^Institute for Asthma & Allergy, Chevy Chase, MD, USA, ^10^AllerVie Health, Birmingham, AL, USA, ^11^The University of Arizona College of Medicine, Phoenix, AZ, USA, ^12^Washington University Physicians, Creve Coeur, MO, USA, ^13^Astria Therapeutics, Boston, MA, USA, 14. Massachusetts General Hospital, Boston, MA, USA

*Allergy, Asthma & Clinical Immunology* 2026, **22(2)**: 95

**Background** Navenibart is a novel investigational therapeutic monoclonal antibody plasma kallikrein inhibitor. We report initial results on safety, efficacy, and QoL from ALPHA-SOLAR, a Phase 2 long-term open-label extension trial (NCT06007677), for participants with HAE-C1INH who completed ALPHA-STAR, a Phase 1b/2 trial (NCT05695248).

**Methods** Participants (n = 16) entering ALPHA-SOLAR were assigned to one of two treatment Arms (Arm A: 600 mg on day 1 and 300 mg every 3 months; Arm B: 600 mg on day 1 and 600 mg on Day 28, followed by 600 mg every 6 months). The primary endpoint was incidence of treatment-emergent adverse events (TEAEs). Secondary endpoints included efficacy. The AE-QoL (Angioedema Quality of Life) Questionnaire, where a lower total score indicates better QoL, was used to assess QoL.

**Results** All 16 target enrollment participants of ALPHA-STAR enrolled in ALPHA-SOLAR. The overall mean (SD) age was 46 (20) years, 9 (56%) were female, and 88% had HAE Type 1. During the mean (median) duration of follow-up of 17.4 (17.1) months across ALPHA-STAR and ALPHA-SOLAR, the most common TEAEs related to navenibart were injection site reactions, occurring in 3 participants. No treatment-related serious adverse events or discontinuations were reported. Overall, in ALPHA-SOLAR [(mean (median) duration of follow-up of 10.1 (9.1) months)], there was a 92% (97%) reduction in mean (median) rate of time-normalized monthly HAE attacks, from 2.22 (2.02) at baseline to 0.17 (0.06) during treatment in ALPHA-SOLAR. Navenibart demonstrated a mean change in AE-QoL total score from baseline of 37.5 in ALPHA-STAR to 13.2 after six months of treatment in ALPHA-SOLAR. Improvement in AE-QoL was demonstrated across all domains.

**Conclusions** These results demonstrate navenibart’s favorable safety profile, durable prevention of HAE attacks, and meaningful improvements in QoL. Navenibart, every 3 and 6 months, is being evaluated in the ongoing global Phase 3 pivotal trial, ALPHA-ORBIT (NCT06842823).

## 96

### Treatment access and outcome gaps in the use of omalizumab (Xolair) for chronic spontaneous urticaria: a retrospective chart revie

#### Almaini, Mustafa, Alkabra, Luna, La, Kevin, George, Candy

##### ACIR, Mississauga, ON

*Allergy, Asthma & Clinical Immunology* 2026, **22(2)**: 96

**Background** Chronic spontaneous urticaria (CSU) significantly aƯects quality of life, often persisting for one to five years. Omalizumab (Xolair) is an approved biologic therapy in Canada for CSU, yet many eligible patients face barriers to initiation and continuity of care. This study explores the real-world gap between eligibility, access, and outcomes for CSU patients oƯered Xolair in a Canadian clinical setting.

**Methods** We conducted a retrospective chart review of adult patients diagnosed with CSU, identified by ICD-10 codes, and oƯered Xolair between 2022 and 2024. Patients were not excluded for discontinuing follow-up due to non-clinical reasons such as relocation, pregnancy, or perceived ineƯicacy, but were classified as “non-completers” for subgroup analysis. Data sources included electronic medical records (CPPs, encounter notes), pharmacy authorization forms, internal insurance logs, UAS7 scores, and documentation of treatment delays or refusal. Outcome measures included initiation rates, time to initiation, denial rates, symptom improvement (UAS7), and physician-documented outcomes.

**Results** Preliminary analysis suggests a substantial gap between clinical eligibility and treatment initiation. A significant proportion of patients meeting CSU criteria were unable to start therapy due to insurance denial or administrative delays. Many non-completers disengaged due to non-medical factors. Among patients who initiated Xolair, UAS7 scores and physician notes indicate clinical improvement. Comparison across subgroups reveals systemic challenges that aƯect equitable access and continuity of care.

**Conclusions** This review highlights real-world barriers that limit eƯective use of biologic therapy in CSU. Administrative and systemic delays, coverage issues, and patient attrition contribute to suboptimal outcomes. Addressing these issues is critical to improving timely access to treatment and consistent follow-up, with the goal of enhancing quality of life for patients with CSU.

**References**

1. Chronic spontaneous urticaria. DermNet. July 24, 2024. Accessed June 26, 2025. https://dermnetnz.org/topics/chronic-spontaneous-urticaria

## 97

### Upadacitinib as a therapeutic option for chronic spontaneous urticaria refractory to antihistamines and omalizumab: a case series

#### Bendayan, Ethan^1^, Zhu, Catherine^4^, Ben-Shoshan, Moshe^2^, Fein, Michael^3^, Netchiporouk, Elena.^4^

##### ^1^Faculty of Medicine, McGill University, Montreal, QC, ^2^Division of Pediatric Allergy and Clinical Immunology, Department of Pediatrics, McGill University Health Centre, Montreal, QC, ^3^Department of Allergy and Clinical Immunology, McGill University Health Centre, Montreal, QC, ^4^Department of Dermatology, McGill University Health Centre, Montreal, QC

*Allergy, Asthma & Clinical Immunology* 2026, **22(2)**: 97

**Background** Chronic spontaneous urticaria (CSU), defined by the occurrence of wheals and/or angioedema without an identifiable trigger for ≥ 6 weeks, is the most common subtype of chronic urticaria, affecting 1–3% of the global population. Type 2B CSU is an autoimmune endotype characterized by IgG autoantibodies against IgE or its high-affinity receptor FcεRI. While second-generation H1-antihistamines (sgAHs) and omalizumab are standard treatments, up to 30% fail to respond adequately to omalizumab, particularly those with Type 2B disease.

**Methods** We recruited patients from a tertiary care center with CSU refractory to sgAHs and omalizumab, who were initiated on upadacitinib, a selective Janus kinase (JAK) inhibitor (15 mg orally daily). Urticaria Activity Score over 7 days (UAS7) and Urticaria Control Test (UCT) scores were recorded for patients before initiation of upadacitinib and 1–4 months post-initiation. Adverse effects were evaluated at follow-up.

**Results** We present a case series of four patients with longstanding refractory CSU (median duration: 3.0 years, IQR: 1.2–4.5), including three women and one man (median age: 37 years, IQR: 28.0–40.0). Three patients had failed treatment with omalizumab, and one with sgAHs alone. All presented with severe baseline symptoms, as indicated by a median UAS7 of 35.5 (IQR: 35.0–39.0), and poor disease control (median UCT score: 2.0, IQR: 1.0–2.5). Given inadequate response to sgAHs (4/4 patients) and omalizumab (3/4), all were initiated on upadacitinib. At follow-up, all patients reported symptomatic improvement and better disease control, with a reduced median UAS7 of 5.5 (IQR: 0–16.0) and increased UCT score of 14.5 (IQR: 9.5–16.0). Upadacitinib was well tolerated in most cases; one patient experienced transient weakness and bone pain.

**Conclusions** This case series highlights the potential role of upadacitinib as an effective and well-tolerated option for patients with CSU refractory to sgAHs and omalizumab. Further studies are needed to evaluate its safety and efficacy in this population.

## 98

### ALPHA-ORBIT—A Phase 3 clinical trial to evaluate the efficacy and safety of navenibart in participants with hereditary angioedema (HAE)

#### Betschel, Stephen D.^1^, Lumry, William R.^2^, Craig, Timothy J.^3^, Zanichelli, Andrea^4^, Aygören-Pürsün, Emel^5^, Anderson, John T.^6^, Magerl, Markus^7^, Tachdjian, Raffi^8^, Banerji, Aleena^9^, VanEenwyk, Claire^10^, Cohen, Theodora^10^, Morabito, Christopher^10^, Hide, Michihiro^11^, Riedl, Marc^12^

##### ^1^University of Toronto, Toronto, ON, ^2^AARA Research Center, Dallas, TX, USA, ^3^Penn State Health Allergy, Asthma and Immunology, Hershey, PA, USA, ^4^Dipartimento di Scienze Biomediche per la Salute, Universitá degli Studi di Milano, Milano, Italy & Unitá Operativa di Medicina, Centro Angioedema, I.R.C.C.S. Policlinico San Donato, San Donato Milanese, Milano, Italy, ^5^Goethe-Universitat, Frankfurt am Main, Germany, ^6^AllerVie Health, Birmingham, AL, USA, ^7^Universitatsmedizin, Berlin, Germany, ^8^UCLA School of Medicine, Los Angeles, Los Angeles, CA, USA, ^9^Massachusetts General Hospital, Boston, MA, USA, ^10^Astria Therapeutics, Boston, MA, USA, ^11^Hiroshima City Hiroshima Citizens Hospital, Hiroshima, Japan, ^12^University of California-San Diego, San Diego, CA, USA

*Allergy, Asthma & Clinical Immunology* 2026, **22(2)**: 98

**Background** In a proof-of-concept trial in HAE patients, navenibart, an investigational novel, long-acting monoclonal antibody against plasma kallikrein, was well tolerated and demonstrated reduced attack frequency, severity, and utilization of on-demand treatment for at least 6 months after 1 or 2 doses. Navenibart has the potential to become an efficacious and safe preventative treatment for HAE, administered 2 or 4 times a year.

**Methods** ALPHA-ORBIT(NCT06842823), a Phase 3, global clinical trial to evaluate the efficacy and safety of navenibart in preventing HAE attacks in adolescent and adult participants with HAE-C1INH, will enroll approximately 145 adults and adolescents. The trial consists of a washout period (≤ 90 days) for adult participants on long-term prophylaxis, a run-in period (≥ 1 month and ≤ 2 months), treatment period (6 months), and a follow-up period (6 months). At treatment initiation, adult participants are randomized to receive either navenibart [(600 mg every 3 months (Q3M), 600 mg then 300 mg Q3M, 600 mg every 6 months (Q6M)] or placebo subcutaneously (double-blinded). Adolescent participants receive navenibart (600 mg then 300 mg Q3M) subcutaneously (unblinded); they do not have a washout period and do not receive placebo. The primary endpoint is the number of time-normalized investigator-confirmed HAE attacks during the 6-month treatment period. Secondary endpoints evaluate the impact of navenibart on burden of disease. The safety endpoint is incidence of treatment-emergent adverse events. After completing the treatment period, participants have the option to roll into the long-term trial (ORBIT-EXPANSE) or complete the safety follow-up through 9 months after the last dose.

**Results** Enrollment began in March 2025 and is ongoing. Results are anticipated in early 2027.

**Conclusions** Efficacy and safety of navenibart, administered every 3 and 6 months, in the long-term prevention of HAE attacks are being investigated in the ongoing global Phase 3 trial, ALPHA-ORBIT.

## 99

### Chronic urticaria with and without angioedema: A cohort study in pediatric and adult populations

#### Moshutz, Angie^1^, Allan, Sienna^1^, Ton That, Alexandre^1^, Netchiporouk, Elena^2, 3^, Fein, Michael^4^, Miedzybrodzki, Barbara^5^, Ben-Shoshan, Moshe^6^

##### ^1^McGill University Faculty of Medicine, Montreal, QC, ^2^Division of Dermatology, McGill University Health Centre, Montreal, QC, ^3^Division of Experimental Medicine, McGill University, Montreal, QC, ^4^Department of Allergy and Immunology, McGill University Health Centre, Montreal, QC, ^5^Division of Pediatric Dermatology, McGill University Health Centre, Montreal, QC, ^6^Division of Pediatric Allergy and Clinical Immunology, McGill University Health Centre, Montreal, QC

*Allergy, Asthma & Clinical Immunology* 2026, **22(2)**: 99.

**Background** Chronic urticaria (CU) is defined by the presence of wheals and/or angioedema lasting six weeks or more. Angioedema may be a marker of more severe CU, though pediatric and adult differences in its presentation and management remain unclear. We aimed to assess clinical patterns and biomarkers in pediatric and adult CU patients, stratified by the presence/absence of angioedema.

**Methods** Data were collected annually on patients with CU followed at the Pediatric Allergy Clinic of Montreal Children’s Hospital and an affiliated external clinic. Patients were divided into pediatric (< 18 years) and adult (≥ 18 years) populations, and further stratified based on the presence or absence of angioedema. Data included demographics, clinical scores (UAS7, UCT), treatment (antihistamines, omalizumab), and biomarkers (IgG anti-TPO). Descriptive statistics were conducted using R program.

**Results** Of the 25 pediatric patients, 28% (n = 7) had angioedema (AE +). AE + children vs. AE- children were predominantly female (86% vs. 69%) with a median diagnosis age of 11.0 vs. 9.0 years, respectively. AE + vs. AE- children reported higher use of high-dose antihistamines (≥ 4 daily: 14% vs. 0%) and omalizumab (14% vs. 6%). Anti-TPO was elevated in 50% of AE + patients and 0% of AE- patients. Angioedema primarily affected the lips, eyelids, and hands (67%).

Among 30 adult patients, 37% (n = 11) had angioedema. AE + vs. AE- adults were predominantly female (82% vs. 53%), with a median age at diagnosis of 38.0 vs. 41.0 years, respectively. AE + adults had higher rates of high-dose antihistamine use (≥ 4 daily: 22% vs. 18%) and omalizumab treatment (30.0% vs. 16%). Anti-TPO was not elevated in AE + adults (0% elevation vs. 50% in AE-). Angioedema mainly involved the eyelids and hands (50%).

**Conclusions** Angioedema was associated with greater disease burden and escalated treatment need in both pediatric and adult CU patients. Differences in biomarker profiles and disease presentation suggest distinct phenotypes.

## 100

### Hereditary alpha tryptasemia among children with chronic urticaria

#### Yu, Xin Xin^1^, Zhu, Catherine^2^, Netchiporouk, Elena^2^, Miedzybrodzki, Barbara^2^, Fein, Michael^3^, Ben-Shoshan, Moshe^3^

##### ^1^Faculty of Medicine and Health Sciences, McGill University, Montreal, QC, ^2^Division of Dermatology, McGill University, Montreal, QC, ^3^Division of Allergy and Clinical Immunology, McGill University, Montreal, QC

*Allergy, Asthma & Clinical Immunology* 2026, **22(2)**: 100

**Background** Hereditary alpha tryptasemia (HαT) is an autosomal dominant trait affecting ~ 5% of the general population and > 90% of those with elevated basal serum tryptase (BST) (≥ 8 ng/mL) due to increased TPSAB1 copies.

**Methods** Pediatric CU patients were prospectively recruited from 2013 to 2025 into the Montreal Urticaria Registry. Participants completed questionnaires on demographics, clinical presentation, CU subtype and management, family history, and weekly urticaria activity score (UAS7) at study entry. Entry blood tests included complete blood counts, C-reactive protein, IgG anti-thyroid peroxidase antibodies, total IgE, and BST levels. Genetic testing for HαT was performed in patients with elevated BST and/or systemic symptoms.

**Results** Of 363 recruited pediatric CU patients, 274 (75.5%) had BST measured; of these, 16 (5.8%) had elevated BST levels. Four of those 16 had consistently elevated levels and were sent for genetic testing. Median age of patients with HαT was 9 years [IQR 3–15]; female:male ratio 3:1. Three had cold-induced urticaria, one severe chronic spontaneous urticaria. Median BST of HαT patients was 14.6 μg/L [IQR 14.1–15.1]. Genetic testing confirmed extra TPSAB1 copies consistent with HαT in three patients (75%). One patient with initially elevated BST had negative repeat tryptase testing and genetic evaluation. All genetically confirmed HαT patients had family members with elevated tryptase. Two patients achieved good control on daily second-generation antihistamines, one resolved without treatment, and one severe case (baseline UAS7 = 28) was well controlled on omalizumab (UAS7 = 0).

**Conclusions** Our findings suggest that HαT is less common among children with CU than in the general population. BST measurement should be considered mainly for cold-induced and severe CU (UAS7 ≥ 16). In patients with persistently elevated BST, genetic testing should be strongly considered. Further analysis of our full CU registry will identify predictors of HαT, including BST levels, subtype (CIndU), and UAS7 scores.

